# Primary tumor associated macrophages activate programs of invasion and dormancy in disseminating tumor cells

**DOI:** 10.1038/s41467-022-28076-3

**Published:** 2022-02-02

**Authors:** Lucia Borriello, Anouchka Coste, Brian Traub, Ved P. Sharma, George S. Karagiannis, Yu Lin, Yarong Wang, Xianjun Ye, Camille L. Duran, Xiaoming Chen, Madeline Friedman, Maria Soledad Sosa, Dan Sun, Erica Dalla, Deepak K. Singh, Maja H. Oktay, Julio A. Aguirre-Ghiso, John S. Condeelis, David Entenberg

**Affiliations:** 1 Department of Anatomy and Structural Biology, Albert Einstein College of Medicine/Montefiore Medical Center, Bronx, NY USA; 2 Gruss-Lipper Biophotonics Center, Albert Einstein College of Medicine/Montefiore Medical Center, Bronx, NY USA; 3 Department of Surgery, Albert Einstein College of Medicine/Montefiore Medical Center, Bronx, NY USA; 4 Integrated Imaging Program, Albert Einstein College of Medicine/Montefiore Medical Center, Bronx, NY USA; 5 Department of Microbiology and Immunology, Albert Einstein College of Medicine/Montefiore Medical Center, Bronx, NY USA; 6 Cancer Dormancy and Tumor Microenvironment Institute and, Einstein Cancer Center, Albert Einstein College of Medicine/Montefiore Medical Center, 1300 Morris Park Avenue, Bronx, NY 10461 USA; 7 Department of Pharmacological Sciences, Icahn School of Medicine at Mount Sinai, New York, NY USA; 8 Department of Cell Biology, Albert Einstein College of Medicine/Montefiore Medical Center, 1300 Morris Park Avenue, Bronx, NY 10461 USA; 9 Division of Hematology and Oncology, Tisch Cancer Institute, Icahn School of Medicine at Mount Sinai, New York, NY USA; 10 Department of Pathology, Einstein College of Medicine/Montefiore Medical Center, Bronx, NY USA

**Keywords:** Breast cancer, Cancer stem cells, Cancer microenvironment

## Abstract

Metastases are initiated by disseminated tumor cells (DTCs) that colonize distant organs. Growing evidence suggests that the microenvironment of the primary tumor primes DTCs for dormant or proliferative fates. However, the manner in which this occurs remains poorly understood. Here, using the Window for High-Resolution Intravital Imaging of the Lung (WHRIL), we study the live lung longitudinally and follow the fate of individual DTCs that spontaneously disseminate from orthotopic breast tumors. We find that spontaneously DTCs have increased levels of retention, increased speed of extravasation, and greater survival after extravasation, compared to experimentally metastasized tumor cells. Detailed analysis reveals that a subset of macrophages within the primary tumor induces a pro-dissemination and pro-dormancy DTC phenotype. Our work provides insight into how specific primary tumor microenvironments prime a subpopulation of cells for expression of proteins associated with dissemination and dormancy.

## Abstract (web summary)

The understanding of the mechanisms underlying the ability of disseminated tumor cells (DTCs) to form metastasis is incomplete. Here, by using high-resolution intravital imaging of the murine lung to track the fate of breast-derived DTCs, the authors show that macrophages within the primary tumor induce a pro-dissemination and pro-dormancy phenotype in tumor cells, favouring their extravasation in the lung.

## Introduction

Metastasis causes approximately 90% of cancer-related mortality^1^. It is the endpoint of a complex and dynamic cascade of steps in which tumor cells migrate within the primary tumor, intravasate, disseminate via the circulatory system, arrest at a secondary site, extravasate, survive, and finally re-initiate growth to form secondary tumors^2^. Most therapeutic interventions targeting metastasis are designed only to attack actively dividing tumor cells (i.e., those in the last step of this cascade; active growth) and do not block any of the intermediate steps. Understanding the mechanisms that regulate the ability of disseminated tumor cells (DTCs) to complete all steps of metastasis will reveal additional targets for novel anti-metastatic therapeutics^3^.

Since 1900, a number of studies have attempted to measure the fate of DTCs during the metastatic cascade using a variety of techniques. These include histopathology of secondary sites such as the lung^4,5^; radioactive labeling and fate-mapping of DTCs in mice^6,7^; ex vivo visualization of DTCs in tissue explants^8^; and in vivo visualization of DTCs in the Chick Chorioallantoic Membrane (CAM)^9,10^ and in secondary sites in mice such as the liver^11^, brain^12^, and lung^13^. However, the results of these studies remain contradictory, alternatively identifying tumor cell survival in the circulation^7,14^, extravasation^15^, and re-initiation of growth/initiation of dormancy^16,17^ as rate-limiting steps.

Furthermore, the vast majority of these studies have relied on a technique called “experimental metastasis” (EM). EM is a process in which tumor cells are injected directly into the vasculature of mice^4^ and is used in place of spontaneous metastasis (SM), where tumor cells in an orthotopic primary tumor stochastically progress through all of the steps of the metastatic cascade^4^. EM assays assume that tumor cells injected as a bolus directly into the vasculature will have the same fate (and provide the same information on metastasis initiation) as do tumor cells which originate from within a primary tumor.

However, a growing literature shows that the presence of a primary tumor can have a profound influence on the metastatic outcome. For example, it has been determined that gene expression signatures in the primary tumor can provide information on the potential for metastatic relapse years after the primary tumor has been removed^18–20^. Furthermore, specific primary tumor microenvironments, such as hypoxia, can prime those primary tumor cells that are destined to become DTCs to have divergent fates in target organs^21^. Despite these advances, it is not known what influence the primary tumor has on the early steps of the metastatic cascade in the secondary site.

In the past, we, and others, have used high-resolution intravital imaging (IVI) to investigate the process of intravasation and dissemination in the primary tumor^22,23^. To understand the influence the primary tumor has on spontaneously disseminating tumor cells in the secondary site, we recently developed the implantable Window for High-Resolution Imaging of the Lung (WHRIL)^24^. Unlike prior techniques, the WHRIL is capable of providing a longitudinal view of the living lung at subcellular resolution over days to weeks, making it possible to follow, quantitatively, the fate of individual disseminated tumor cells throughout the process of colonization.

To our knowledge, spontaneously disseminating tumor cells have never been quantitatively measured, in real-time, during the process of metastasis to the lung. Thus, we here employed this tool to directly and longitudinally visualize the secondary site with single-cell resolution IVI throughout the process of spontaneous metastasis in order to directly measure the primary tumor’s influence on the initial steps of the metastatic cascade in the secondary site. We find that, compared to intravascularly injected tumor cells, spontaneously DTCs have increased levels of retention, extravasate from the lung vasculature more quickly, and have a greater rates of survival after extravasation. Our investigation into the mechanism underlying these phenotypes reveals that a subset of macrophages within the primary tumor activates programs of dissemination and dormancy in DTCs as they approach intravasation sites.

## Results

### Real-time observation of tumor cell arrival to the lung

**Fig. 1:**
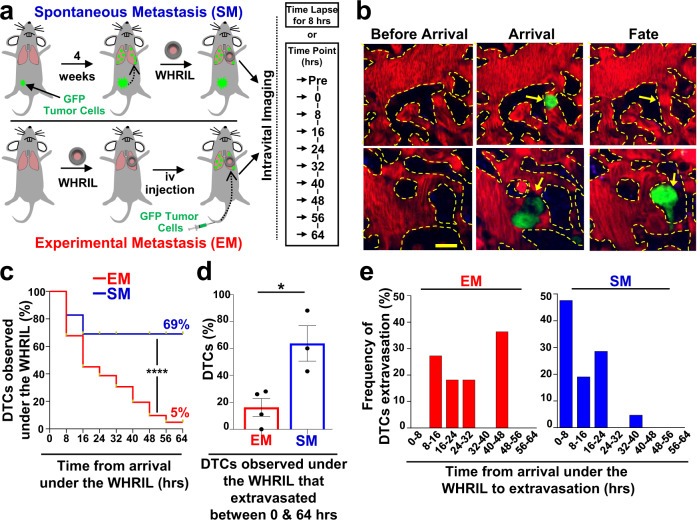
Tumor cells that spontaneously disseminate from the primary tumor to the lung have a drastically increased metastatic efficiency compared to intravenously injected tumor cells. **a** Experimental design to track the fate of individual disseminated tumor cells using an Experimental Metastasis (EM) model (top) and a Spontaneous Metastasis (SM) model (bottom). **b** Serial imaging through the WHRIL allows tracking of the fate of disseminated tumor cells. Vasculature before arrival (left), upon arrival (middle) and after arrival of tumor cells (TCs). Fate of TCs (yellow arrows) could be either recirculation or apoptosis (right, top) or extravasation into the lung parenchyma (right, bottom). Red = tdTomato labeled endothelial cells and 155 kDa Tetramethylrhodamine dextran labeled blood serum, Green = GFP labeled tumor cells, Blue = second harmonic generation. Scale bar = 15 μm. **c** Kaplan-Meier survival curves showing the percentage of E0771-GFP tumor cells observed under the WHRIL at each 8 hr time point over a period of 64 hrs. EM: *n* = 62 tumor cells analyzed in 3 mice. SM: *n* = 29 tumor cells analyzed in 3 mice. Log-rank (Mantel-Cox) test (*p* < 0.0001). ****p < 0.0001. **d** Percentage of E0771-GFP EM and SM tumor cells observed under the WHRIL that extravasated between 0 and 64 hrs after arrival. EM: *n* = 89 tumor cells in 4 mice. SM: *n* = 29 tumor cells in 3 mice. Bar = mean. Error bars = ±SEM. Unpaired two-tailed *t*-test (*p* = 0.017). *p < 0.05. **e** Quantification of the time from arrival under the WHRIL to extravasation into the lung parenchyma for each E0771-GFP EM and SM tumor cell. **Left:** EM: *n* = 11 tumor cells in 3 mice. **Right:** SM: *n* = 21 tumor cells in 3 mice. Source data are provided as a Source Data file.

We began our investigation by generating mouse models of spontaneous metastasis. For these studies, we specifically chose to use orthotopic injections of cancer cell lines, and not transgenic animal models of cancer, so as to be able adhere closely to earlier studies of metastasis, which all used cancer cell lines^7,15–17,25,26^. Specifically, we developed orthotopic primary breast tumors in mice using E0771 cells labeled with green fluorescent protein (E0771-GFP) and grown in syngeneic C57/B6J VeCad-tdTomato mice (Fig. 1a, top) or, to ensure that the observed biological findings were generalizable, using human tumor cells (MDA-MB-231) labeled with GFP (231-GFP) grown in nude immunocompromised mice. To observe DTCs in the lung, we implanted the WHRIL into tumor-bearing mice, and 24 hrs later, imaged them with intravital imaging (IVI) using multiphoton microscopy (Supplementary Movie 1), as previously described^24^. Using this method, we were able to visualize both intravascular, extravascular tumor cells (Supplementary Fig. 1a, b, Supplementary Movies 2 & 3), and pre-existing metastases, and to observe the arrival of new DTCs to the lung vasculature in real time (Fig. 1b, “Arrival”, top and bottom).

Similar to observations made with vacuum-based imaging windows for the lung^13^, we observed that tumor cells arriving to the lung vasculature completely fill the capillary lumen and exclude all blood serum (as indicated by IV-injected fluorescent dextran), suggesting that these cells are arrested due to physical restraint rather than active attachment. We did not find that tumor cells roll along the vasculature or otherwise migrate from their place of lodgment, as was previously observed in lung explants^8^ or in zebrafish^27,28^. We never observed tumor cells to proliferate intravascularly, as reported by Al-Mehdi et al.^8^. We also never observed CTC clusters traversing capillaries as was observed in vitro by Au et al.^29^.

Since the progression of tumor cells through the metastatic cascade takes much longer than 8 hrs, we posited that the fate of disseminated tumor cells could be followed by imaging the vasculature once every 8 h, using in vivo microcartography^24^ to relocate the same cells and microvasculature during each imaging session. To rule out the possibility that tumor cells may migrate in or out of the field of view between imaging sessions, we analyzed our time-lapsed images to assess the motility of spontaneously metastasizing tumor cells before and after extravasation. As can be seen in Supplementary Fig. 1c Left & 1d Left, we never observed tumor cells migrating beyond a single field of view. In fact, we were surprised to find that tumor cells were largely immobile. Based on our measurements, we projected that the fastest cells would take, at a minimum, 69 h to move from the center of the field of view to its boundary (256 µm). Thus, we next undertook evaluations of the long-term fate of disseminated tumor cells by capturing a single image of the vasculature once every eight hours.

### Spontaneously metastasizing tumor cells are retained in the lung significantly longer and in higher numbers compared to intravenously injected tumor cells

To track the fate of each newly arriving DTC as it progresses through the metastatic cascade, we prepared mice using the same procedure described above and then directly visualized the lung vasculature to record the presence of any previously disseminated tumor cells (Fig. 1a, Time Point “Pre”), or the empty vasculature (Fig. 1b, Before Arrival). The location of each preexisting tumor cell (including pre-existing metastases) was recorded and these locations excluded from further analysis, as their precise arrival time was unknown. After 8 h, we again imaged the vasculature using the WHRIL and recorded the locations of all newly arrived DTCs. These images constitute *t* = 0 (Fig. 1a, Time Point 0 hrs) with cells arriving sometime between 0 and 8 h after the start of the experiment.

We then imaged the lung vasculature every 8 h thereafter (Fig. 1a, Time Points 8 through 64 h), using microcartography to predict to the same location for each time point. This location was verified by visual inspection of the unique architecture of the microvasculature. The fate of each tumor cell was determined as described in Fig. 1b, which depicts the lung vasculature before the arrival of a tumor cell (Fig. 1b, “Before Arrival”, top and bottom), the lodged tumor cell upon arrival (Fig. 1b, “Arrival”, top and bottom), and finally the fate of the tumor cell as either death or recirculation within the vasculature (Fig. 1b, “Fate”, top), or extravasation (Fig. 1b, “Fate”, bottom).

With this methodology, we determined the arrival and subsequent disappearance or extravasation of tumor cells within 8 h timeframes. Unexpectedly, we found that, after an initial modest decline, most E0771-GFP tumor cells (~70%) were retained in the lung for the entire experimental period (Fig. 1c, blue curve). Though, we observed a greater initial decline in the 231-GFP model, the number of tumor cells retained in the lung also persisted in this model, so that ~35% remained after 64 h (Supplementary Fig. 2a, blue curve). This was in stark contrast to previous reports where it was observed that DTCs die off and are rapidly cleared from the tissue^7,25,30–32^. To eliminate the use of the WHRIL as a contributing variable, we sought to repeat these experiments with the same experimental metastasis model used in the prior publications.

After repeating our time-lapse imaging validation experiments to ensure that EM cells cannot migrate out of a field of view within 8 h (Supplementary Fig. 1c Right & 1d Right), we established a comparable method for tracking DTCs within the experimental metastasis model. We implanted the WHRIL into mice and then 24 h later, we injected the same tumor cells into their tail veins (E0771-GFP in syngeneic C57/B6 or 231-GFP in nude mice). We then immediately began imaging the vasculature under the WHRIL. These images thus constitute *t* = 0 for the EM model (Fig. 1a, bottom, Time Point 0 h). We found that, as we had initially expected, E0771-GFP EM tumor cells were rapidly cleared from the lung, with a greater than 50% drop within the first 16 h, and a steady decline thereafter, leaving only 5% after 64 h (Fig. 1c, red curve). This represents a 10-fold increase in tumor cell retention of SM compared to EM tumor cells.

Consistently, we observed that the number of 231-GFP EM tumor cells also rapidly declined to ~40% within the first 16 h, and then continued to decline steadily until less than 2% remained at 64 h postinjection (Supplementary Fig. 2a, red curve). Although the difference between EM and SM in this model was not as large as in the syngeneic model, the difference between the two models persisted and remained significant, confirming that disseminating tumor cells that originate in a primary tumor are retained longer, and in higher numbers, within the secondary site of the lung.

### Spontaneously metastasizing tumor cells extravasate more quickly than intravenously injected tumor cells

It has been hypothesized that extravasation is a major rate-limiting step in metastasis because tumor cells are killed by the mechanical trauma that they are subjected to in the circulatory system^33,34^, and when tumor cells are able to extravasate quickly, they metastasize more efficiently^5^. We therefore posited that the increased rate of retention of SM cells is due to a better ability to extravasate. To test this, we used the WHRIL to analyze the number of tumor cells that extravasated within the experimental time period (between 0 and 64 h). While only 16% of EM cells were able to extravasate, a significantly greater proportion of SM cells (64%) was able to extravasate during the same time period after arrival to the lung (Fig. 1d). Interestingly, while we did observe a slight increase in the ability of SM cells to extravasate in the 231-GFP model, this was not statistically different from EM cells (Supplementary Fig. 2b).

The observation that syngeneic SM cells extravasate more efficiently prompted us to evaluate how long individual tumor cells arriving in the lung take to cross the vascular endothelium. We therefore imaged SM and EM cells every 8 h after their arrival under the WHRIL and observed no EM cells to extravasate between 0 and 8 h after intravenous (iv) injection. The distribution of extravasation times was wide with many cells taking as long as 40–48 h to cross the endothelium (Fig. 1e, red bars) with the average time of extravasation being 28.0 ± 4.1 h. In contrast, we found that a much larger proportion of SM cells was able to cross the endothelium quickly, with ~50% doing so within 0–8 h from the time of the first arrival to the lung vasculature (Fig. 1e, blue bars). By 24 h, the vast majority of SM tumor cells had extravasated. Moreover, the average time of extravasation for SM cells was nearly half that for EM cells (11.6 ± 1.9 hrs, Fig. 1e). Similar observations were made with 231-GFP cells, where only ~30% of EM tumor cells extravasated within the 0–8 h period while ~80% of the SM cells extravasated in the same period (Supplementary Fig. 2c). While the distribution of extravasation times was not as wide for the EM 231-GFP cells as was observed for the EM E0771-GFP cells, the difference in means persisted with EM cells extravasating on average at 11.8 ± 1.0 h vs. 6.4 ± 0.8 h for SM cells. These data demonstrate that disseminating tumor cells that originate in a primary tumor extravasate much more quickly than intravenously injected tumor cells.

### Expression of Mena^INV^ confers early extravasation competency to disseminated tumor cells

Our previous studies^35–37^ showed that expression of alternative splice variants of the actin regulatory protein, Mena, confer dramatically different metastatic phenotypes to tumor cells^38^. Expression of the isoform, Mena11a, is associated with an epithelial, non-metastatic phenotype. Meanwhile Mena^INV^, an isoform not expressed in tumor cells cultured in vitro, but induced upon contact with macrophages, enhances tumor cell migration and transendothelial migration during intravasation within the primary tumor^35–37^.

**Fig. 2:**
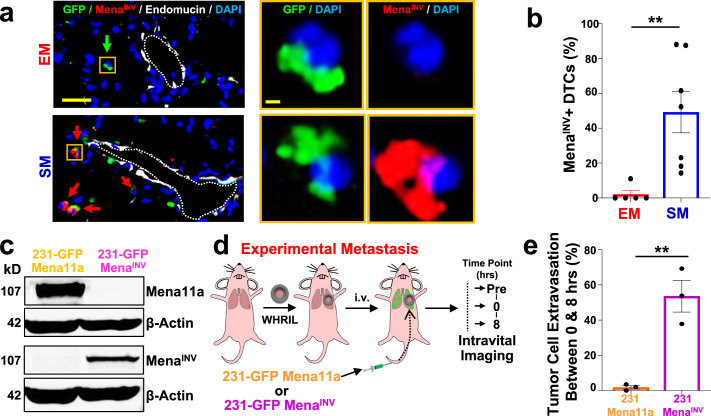
Mena^INV^ expression in disseminated tumor cells regulates extravasation efficiency. **a Left:** Mena^INV^ expression in extravascular E0771-GFP tumor cells in the lung of an EM model (top) and an SM model (bottom). Green arrow: Mena^INV^ negative tumor cell. Red arrows: Mena^INV^ positive tumor cells. Scale bar = 50 μm. **Right:** Zoomed in view of a disseminated tumor cell (yellow box) in both models. Green = GFP, Red = Mena^INV^, White = endomucin, Blue = DAPI. Scale bar = 10 μm. **b** Quantification of extravascular Mena^INV^ positive disseminated tumor cells in the lung of each group from Fig. 2a. EM: *n* = 41 cells in 5 animals. SM: *n* = 89 cells in 7 animals. Bar = mean. Error bars = ±SEM. Two-tailed Mann-Whitney test (*p* = 0.0025). **p < 0.01. **c** Western blot of 231-GFP-Mena11a and 231-GFP-Mena^INV^ cells. β-Actin was used as a loading control. Full blot is presented in Supplementary Fig. 16. **d** Outline of experimental design to determine percentage of tumor cells able to extravasate between 0 and 8 h after iv-injection. Intravital imaging of the lung vasculature through the WHRIL was performed before (Timepoint = “Pre”), immediately after (Time point = 0 h), and then finally 8 h after tumor cell injection (Time point = 8 h). **e** Percentage of tumor cells that extravasated between 0 and 8 h after iv injection. 231-GFP-Mena11a: *n* = 90 cells in 3 mice. 231-GFP-Mena^INV^: *n* = 88 tumor cells in 3 mice. Bar = mean. Error bars = ±SEM. Two-tailed unpaired *t*-test (*p* = 0.0048). **p < 0.01. Source data are provided as a Source Data file.

Therefore, we hypothesized that Mena^INV^ expression could play a similar role during extravasation at the secondary site by conferring to tumor cells the ability to cross the endothelium quickly, a role never before explored for this protein. As a preliminary test, we took tissue sections from the lungs of the EM and SM models and stained them for GFP (to identify tumor cells), Mena^INV^, endomucin (to identify the vasculature), and DAPI (to identify all nuclei) (Fig. 2a). We found a ~25-fold increase (49% vs. 2%) in the percentage of SM tumor cells expressing Mena^INV^, compared to EM cells (Fig. 2b). In the 231-GFP model, the percent of cells expressing Mena^INV^ was ~4 times higher *(*48% vs. 13%) for SM compared to EM cells (Supplementary Fig. 2d, e). This shows that Mena isoform expression in tumor cells retained in the lungs is significantly different between SM and EM cells.

To determine whether the expression of Mena^INV^ directly confers the ability to extravasate early (within 8 hr of arrival in the lung vasculature), we performed gain- and loss-of-function experiments using two genetically modified 231-GFP cell lines: one that overexpresses Mena^INV^ (231-GFP-Mena^INV^) and one that overexpresses Mena11a but does not express Mena^INV^ at all (231-GFP-Mena11a)^39^ (Fig. 2c). Using these two cell lines, we performed the experimental metastasis assay and tracked the number of tumor cells that had extravasated after 8 hr (Fig. 2d). Over 50% of the 231-GFP-Mena^INV^ cells that arrived under the WHRIL extravasated within the first 8 hr post-injection, while lack of Mena^INV^ expression almost completely abrogated tumor cell extravasation within this time period (Fig. 2e). This resulted in a nearly 30-fold increase in early extravasation of Mena^INV^ (54%) compared to Mena 11a (2%) expressing cells. These data show that the selective expression of Mena^INV^, which is only upregulated in tumor cells that have successfully intravasated, greatly enhances the efficiency of early extravasation of DTCs into secondary sites.

### Spontaneously metastasizing tumor cells survive significantly longer at the secondary site compared to intravenously injected tumor cells

**Fig. 3:**
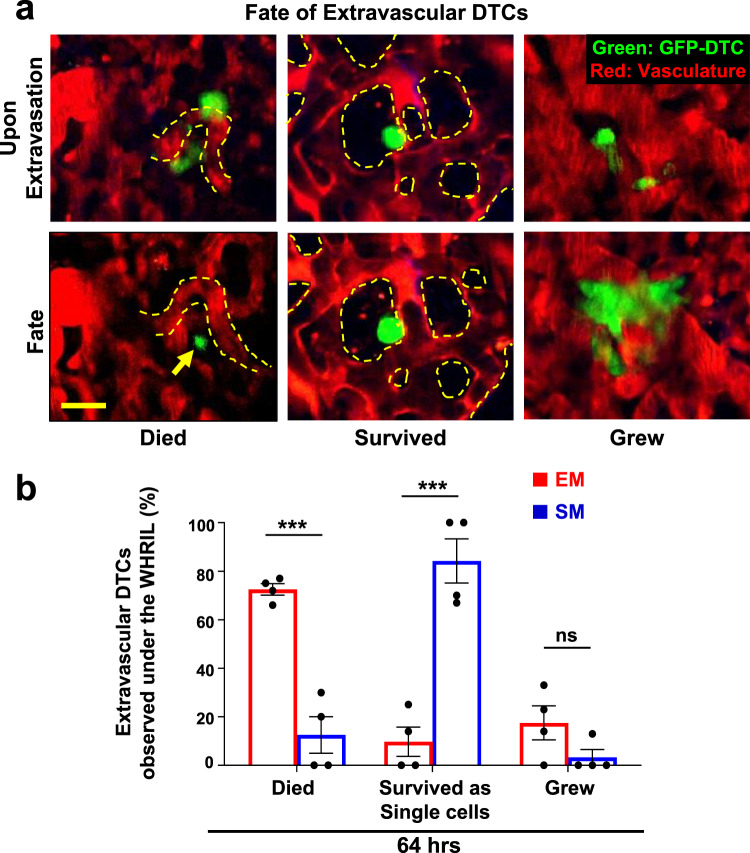
Spontaneously metastasizing tumor cells survive significantly longer at the secondary site compared to intravenously injected tumor cells. **a** Representative intravital microscopy images showing the possible fates of extravascular disseminated tumor cells in the lung parenchyma. **Top:** Images of disseminated tumor cells just after extravasation. **Bottom left:** Example of an extravascular tumor cell, which has died, as evidenced by small extravascular apoptotic bodies (yellow arrow). **Bottom middle:** Example of an extravascular tumor cell that survived as a single and solitary tumor cell over time. **Bottom right:** Example of an extravascular tumor cell that began to divide and grow into a micro-metastasis. Red = tdTomato labeled endothelial cells and 155 kDa Tetramethylrhodamine dextran labeled blood serum, Green = GFP labeled tumor cells. Yellow dashed lines delineate blood vessel boundaries. Scale bar = 15 μm. **b** Percentage of extravascular E0771-GFP disseminated tumor cells that died, survived, or grew after extravasation in EM and SM models 64 hrs after arrival to the lung vasculature. EM: *n* = 27 tumor cells in 4 mice. SM: *n* = 31 tumor cells in 4 mice. Bar = mean. Error bars = ±SEM. For Died and Survived columns, a two-tailed unpaired t-test was used (*p* = 0.0003 and 0.0005, respectively). For Grew columns, a two-tailed Mann-Whitney test was used (*p* = 0.14). ***p < 0.001. ns = not significant. Source data are provided as a Source Data file.

It is generally accepted that, after extravasation into the lungs, only a very small percentage of tumor cells survives^40^. Given the differences between SM and EM observed thus far, we aimed to test whether the presence of the primary tumor influences tumor cell survival after extravasation in the lung. Thus, we imaged the lung vasculature in the SM and EM models, as described in Fig. 1a, every eight hours for 64 hrs to track each individual tumor cell longitudinally. There were three possible outcomes for disseminated tumor cells: they died (Fig. 3a, left), evidenced by the presence of GFP+ cellular debris in the parenchyma, as was previously observed by Kienast et al.^12^ (Fig. 3a, left, yellow arrow); they survived in the lung parenchyma as single cells (Fig. 3a, middle); or they grew into micro-metastases (Fig. 3a, right).

We found that a large majority (72%) of EM cells died shortly after extravasation, with a 28% survival rate at 64 hrs. Of these, 10% remained as single cells at 64 h postinjection and 18% began to form micro-metastasis (Fig. 3b left, red bars). By 64 h after extravasation, the vast majority of SM cells (84%) survived as single and solitary tumor cells and only a small percentage (13%) died. Of the surviving cells, only a small subset formed micrometastasis (3%) during this time period (Fig. 3b left, blue bars). We find no statistical difference in the number of cells that eventually developed into micro-metastasis between the two models. Similar observations were made with 231-GFP cells (Supplementary Fig. 2f). These data demonstrate that disseminated tumor cells originating in a primary tumor have a drastically increased ability to extravasate and successfully survive as solitary cells in the lung parenchyma compared to intravenously injected tumor cells.

### Spontaneously metastasizing tumor cells have higher expression of dormancy markers than intravenously injected tumor cells

The observation that the vast majority of SM cells survive as single and solitary cells, without dying or proliferating during the first 64 hrs of their residency in the lung, suggested that they may have become dormant. This is consistent with earlier studies using EM to disseminate tumor cells in the CAM^9,26^, liver^41^, and lung^17^. However, in those studies, the dormant state of the tumor cells was only determined using tools that assess active proliferation (absence of division, Ki-67 expression). This prompted us to assess if the solitary DTCs we found in the SM model are also in a dormant state using more recently discovered markers. One of the best molecular markers of dormancy is the orphan nuclear receptor NR2F1^42,43^, a member of the retinoic acid receptor family^43^ that has been shown to be a marker for cancer dormancy in pre-clinical models and cancer patients, as well to be an independent prognostic indicator for time-to-distant-recurrence in breast cancer patients^42^. Furthermore, NR2F1 has been shown to regulate tumor dormancy in different mouse models, including breast cancer^20,21,43–45^.

**Fig. 4:**
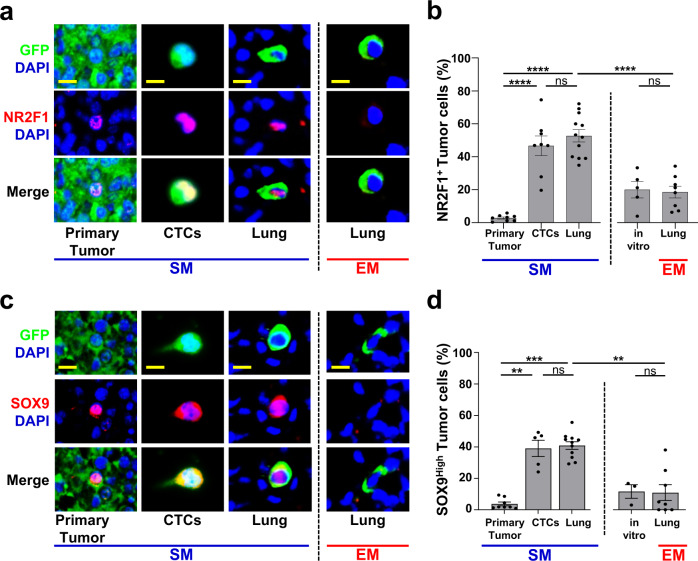
Spontaneously metastasizing tumor cells are more frequently positive for dormancy or stem-like markers compared to intravenously injected tumor cells. **a** Representative immunofluorescence images of NR2F1 expression in primary tumors, circulating tumor cells (CTCs), and disseminated tumor cells (Lung) from an E0771-GFP SM model (Left) and in disseminated tumor cells (Lung) from an EM model (right). Green = GFP, Red = NR2F1, Blue = DAPI. Scale bar for Primary Tumor = 50 μm. Scale bar for CTCs and Lung = 15 μm. **b** Percentage of NR2F1-positive and negative tumor cells in each group in **A**. Primary Tumor: *n* = 2,041 cells in 115 fields of view (65 × 65 µm^2^) in 8 animals; CTCs: *n* = 550 cells in 8 animals; SM Lung: *n* = 237 cells in 12 animals; EM Lung: *n* = 199 cells in 8 animals. In vitro: *n* = 463 cells in 5 independent experiments. Bar = mean. Error bars = ±SEM. For all comparisons, a two-tailed one-way ANOVA test with Sidak’s multiple comparisons adjustment was used (PT vs. CTC: *p* < 0.0001; PT vs. Lung SM: *p* < 0.0001; CTC vs. Lung SM: *p* = 0.80; Lung SM vs. Lung EM: *p* < 0.0001; In vitro vs. Lung EM: *p* = 1.00). ****p < 0.0001. ns = not significant. **c** Representative immunofluorescence images of SOX9 expression in primary tumors, circulating tumor cells (CTCs), and disseminated tumor cells (Lung) from an E0771-GFP SM model (Left) and in disseminated tumor cells (Lung) from an EM model (right). Green = GFP, Red = SOX9, Blue = DAPI. Scale bar for Primary Tumor = 50 μm. Scale bar for CTCs and Lung = 15 μm. **d** Percentage of SOX9^High^ tumor cells from each group in **C**. Primary Tumor: *n* = 4,633 cells in 150 fields of view (65 × 65 µm^2^) in 8 animals; CTCs: *n* = 558 cells in 5 animals, SM Lung: *n* = 341 cells in 11 animals; EM Lung: *n* = 182 cells in 8 animals. In vitro: *n* = 298 cells in 3 independent experiments. Bar = mean. Error bars = ±SEM. For CTC vs. Lung SM (*p* = 0.89) a two-tailed one-way ANOVA test with Sidak’s multiple comparisons adjustment was used. For PT *vs*. CTC: (*p* = 0.0027), PT vs. Lung SM (*p* = 0.0002), In vitro vs. Lung EM (*p* = 1.00), and Lung SM vs. Lung EM (*p* = 0.0011) a two-tailed Kruskal-Wallis test with Dunn’s multiple comparisons adjustment was used. **p < 0.01. ***p < 0.001. ns = not significant. Source data are provided as a Source Data file.

In tissue and CTCs from both metastasis models, we could find single tumor cells (GFP+) that expressed nuclear NR2F1 (Fig. 4a). We found that in lung tissues, the frequency of disseminated tumor cells expressing nuclear NR2F1 was upregulated ~3-fold in SM cells when compared to EM cells (53% vs. 19%) (Fig. 4b, “Lung” columns), suggesting that a significantly greater proportion of spontaneously disseminated tumor cells enter dormancy as supported by prior studies using SM protocols^43,45^. We also observed numerous instances of NR2F1-positive tumor cells located inside the lung vasculature (Supplementary Fig. 3). This raised the question of whether SM tumor cell dormancy was initiated after arrival to the lung vasculature, or if it occurred within the primary tumor, as we previously showed happens under hypoxic conditions^21^. We therefore quantified NRF21+ cells in primary tumor tissues and circulating tumor cells (CTCs) from the SM model (Fig. 4a, Supplementary Fig. 4a). Only a very small percentage (~3%) of primary tumor cells were positive for NR2F1 (Fig. 4b, “Primary Tumor” bar), consistent with data from head and neck squamous cell carcinoma (HNSCC) PDX models and human HNSCC tumors^21^ and the mouse mammary tumor virus-polyoma virus middle T antigen (MMTV-PyMT) model^21^. Despite there being only a small number of NR2F1-positive primary tumor cells, we observed that ~50% of CTCs were NR2F1-positive (Fig. 4b, “CTCs” bar), suggesting that tumor cells are programmed for dormancy either before they intravasate (since acquisition can occur in hypoxic microenvironments^21^) or during intravasation. This represents a very significant enrichment (~17-fold) upon intravasation. Given the short residence time of the cells in the vasculature as CTCs, it is unlikely this enrichment is due to the death of non-dormant cells within the systemic circulation. This high level of NR2F1 DTC positivity was also found in disseminated tumor cells in the lung tissue (Fig. 4b, left “Lung” bar). Importantly, we did not observe a difference between the percentage of NR2F1-positive cells present in vitro, before injection, and the fraction of EM cells observed in the lung three days postinjection (Fig. 4b, right, “in vitro” & “Lung” bars), indicating that expression of NR2F1 is not influenced by passage through the blood. Similar observations were made with 231-GFP cells (Supplementary Fig. 4b), though the percentage of NR2F1 cells within the SM lung was significantly lower than in the CTCs. Overall, these data show that a larger proportion of tumor cells that originate in a primary tumor and arrive to the lungs express dormancy markers compared to intravenously injected tumor cells.

### Spontaneously metastasizing tumor cells are more frequently positive for both dormancy and stem-like markers compared to intravenously injected tumor cells

Recently, it was shown^43^ that NR2F1 in tumor cells coordinates the expression of other genes that are found in self-renewing embryonic stem cells (e.g. SOX9, SOX2, and NANOG^46^) and that can themselves coordinate NR2F1^47^. In particular, it was discovered that NR2F1 binds directly to the promoter of SOX9 to regulate SOX9 expression in tumor cells, resulting in dormancy and growth arrest^43^. Furthermore, it was observed that SOX2 mRNA is significantly upregulated in dormant tumor cells^43^. Based on these observations, we hypothesized that tumor cells originating in a primary tumor may take on a more stem-like and growth arresting phenotype compared to intravenously injected tumor cells as part of the dormancy program that is induced in the primary tumor.

To address this, lung tissue sections from EM and SM models, primary tumor tissues, and CTCs from the SM model, were stained for SOX9 (Fig. 4c). The expression of SOX9 in SM cells in the lung was ~4-fold higher when compared to EM cells (41% vs. 11%, Fig. 4d). As with NR2F1, we found only a small population (4%) of SOX9^High^ cells in the primary tumor (Fig. 4d, “Primary Tumor” bar), but a dramatic enrichment of SOX9^High^ CTCs (39%, a ~10-fold increase, Fig. 4d, “CTCs” bar), suggesting that tumor cells are programmed for stemness in the primary tumor, before, or during intravasation. Again, we did not observe a difference between the percentage of SOX9^High^ cells in vitro and the fraction of EM cells in the lung (Fig. 4b, right, “in vitro” & “Lung” bars), suggesting that induction of a stem-like program does not occur in the blood. Similar observations were made with 231-GFP cells (Supplementary Fig. 4c, d).

**Fig. 5:**
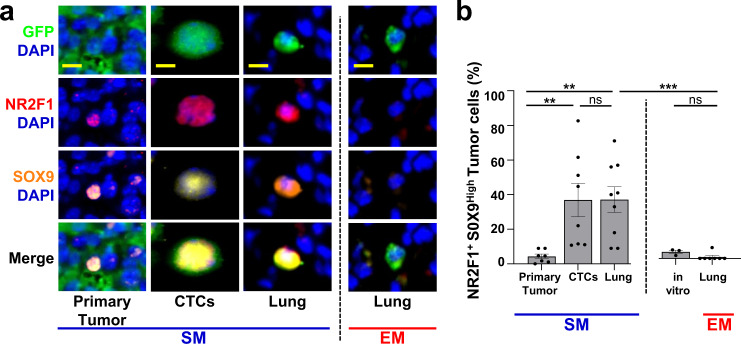
Spontaneously metastasizing tumor cells are more frequently doubly positive for dormancy and stem-like markers compared to intravenously injected tumor cells. **a** Representative images of triple immunofluorescence staining for GFP, NR2F1, and SOX9 expression in primary tumors, circulating tumor cells (CTCs), and disseminated tumor cells (Lung) from an E0771-GFP SM model (Left) and in disseminated tumor cells (Lung) from an EM model (Right). Green = GFP; Red = NR2F1; Orange = SOX9; Blue = DAPI. Scale bar for Primary Tumor = 50 μm. Scale bar for CTCs and Lung = 15 μm. **b** Percentage of double-positive tumor cells NR2F1-positive SOX9^High^ from each group in Fig. 5a. Primary Tumor: *n* = 2383 in 97 fields of view (65 × 65 µm^2^) in 7 animals; CTCs: *n* = 379 cells in 8 animals; SM Lung: *n* = 104 cells in 9 animals; In vitro: *n* = 413 cells in 3 independent experiments. EM Lung: *n* = 75 cells in 7 animals. Bar = mean. Error bars = ±SEM. For EM Lung vs. SM Lung (*p* = 0.0001) and EM Lung vs. in vitro (*p* = 0.69), a two-tailed Kruskal-Wallis test with Dunn’s multiple comparisons adjustment was used. For PT *vs*. CTC: (*p* = 0.0041), PT vs. Lung SM (*p* = 0.0030), and CTC *vs*. Lung SM (*p* = 1.00) a two-tailed ANOVA test with Sidak’s multiple comparisons adjustment was used. **p < 0.01. ***p < 0.001. ns = not significant. Source data are provided as a Source Data file.

Given the mechanistic link between NR2F1 and SOX9 expression^43^, we determined whether disseminated tumor cells co-express NR2F1 and SOX9, which would result in both quiescence and self-renewal (a stem-like program, such as exists in adult quiescent stem cells). Lung tissue from EM and SM models, primary tumor tissues, and CTCs from the SM model were stained for GFP, NR2F1, SOX9, and DAPI (Fig. 5a & Supplementary Fig. 4e). In the primary tumor, we observed only a small population (4%) that co-expressed NR2F1 and SOX9. However, SM cells were enriched for double-positive CTCs and DTCs in the lung compared EM cells (37% vs. 1% for each, Fig. 5b). Similar observations were made with 231-GFP cells (Supplementary Fig. 4f). Overall, these data show that SM tumor cells become progressively more enriched in dormancy and stem-like phenotypes (as evidenced by expression of markers of dormancy and stemness) as they disseminate from the primary tumor to the lung.

### NR2F1 regulates the fate of extravasated tumor cells

These data raise the question of what impact NR2F1 expression has on the ability of DTCs to metastasize. We previously demonstrated that NR2F1 in tumor cells does impact metastasis, with its downregulation increasing metastasis in the lung and spleen, as well as in surgery margins where dormant residual cells persist^43^, and its activation suppressing metastatic growth post-extravasation^45^. However, it is still unknown what impact this downregulation has on the ability of tumor cells to extravasate, or whether cells with downregulated NR2F1 are immediately able to begin proliferating in the lung.

To address the question of what impact reduction of NR2F1 expression has on the ability of tumor cells to extravasate, we downregulated NR2F1 in tumor cells using the Tet-ON-inducible shRNAmir-NR2F1 (Supplementary Fig. 5a–c), as previously described^43^. We then tail vein injected these cells and measured their kinetics of extravasation using intravital imaging, tracking the number of tumor cells that extravasated between 0 and 24 hrs (Supplementary Fig. 5d top). We found that, while downregulation of NR2F1 limits the extravasation ability of tumor cells by ~10%, still ~25% of the DTCs with knockdown are able to extravasate, suggesting a minor role for NR2F1 on this step (Supplementary Fig. 5e).

To determine whether reduction of NR2F1 expression makes DTCs begin to proliferate immediately, we tail vein injected shNR2F1 DTCs into mice both with and without doxycycline (to regulate Tet-ON-inducible shRNAmir-NR2F1) and then stained their lungs for GFP to identify micro-metastatic foci at 4 and 8 days after injection. We found that neither knockdown nor NR2F1-expressing DTCs begin proliferating immediately, but knockdown cells are significantly more proliferative by 8 days postinjection (Supplementary Fig. 5f). We additionally confirmed these findings in a spontaneous metastasis model (Supplementary Fig. 5d, bottom & Supplementary Fig. 5g).

Overall, these data demonstrate that NR2F1 does determine the fate of DTCs, and its regulation affects metastatic growth primarily by regulating a post-extravasation dormant state combined with a less significant contribution to extravasation.

### NR2F1 and Mena^INV^ positive tumor cells are preferentially associated with TMEM doorways in the primary tumor

Given the dramatic increase in dormancy markers as tumor cells move from the primary tumor into the vasculature, we hypothesized that the dormancy program may be activated near to, or at, intravasation sites. Our prior work^48–52^ has shown that tumor cells intravasate through cellular doorways in the vasculature called tumor microenvironment of metastasis (TMEM) doorways. This stable triple cell complex is composed of a Mena^High^ tumor cell, a Tie2^High^ macrophage, and a blood vessel endothelial cell, all in direct physical contact. We have shown that TMEM doorways are the sole sites of breast cancer cell intravasation^53^ and we have clinically validated the density of TMEM doorways as a prognostic marker for metastatic recurrence in breast cancer patients^52^. Thus, programming for dormancy might be induced as migratory tumor cells approach, interact with, or reside in the vicinity of TMEM doorways.

**Fig. 6:**
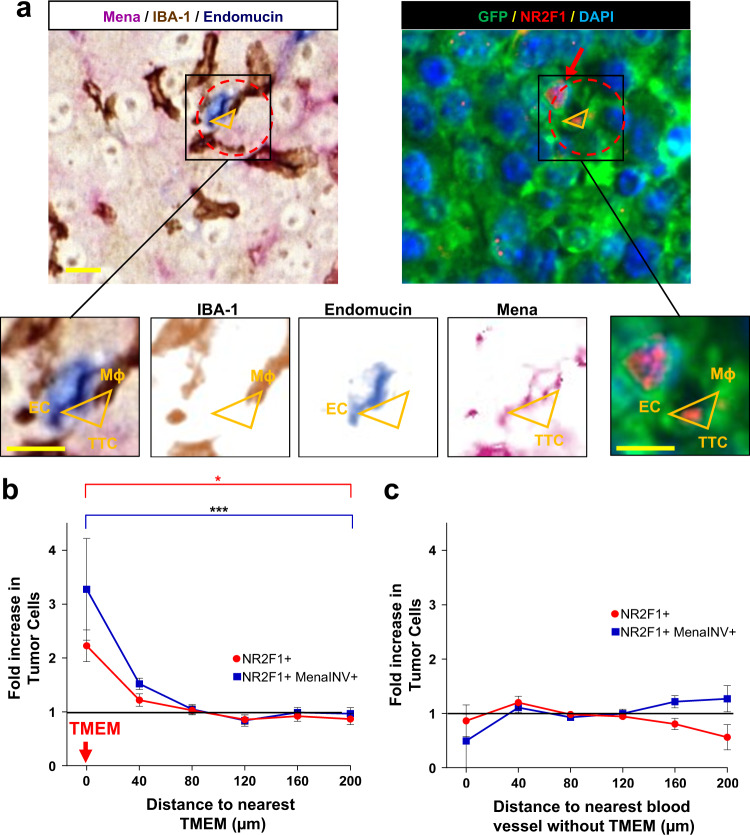
Dormant tumor cells are preferentially associated with TMEM doorways in the primary tumor. **a Left:** Representative image of triple immunohistochemical stain in E0771-GFP primary tumor for the cells composing TMEM doorway positioned at vertices of yellow triangle: Mena expressing tumor cells = pink; IBA-1 expressing macrophages = brown; endomucin expressing endothelial cells = blue. Red dashed circle encompasses the perimeter of TMEM doorway. Scale bar = 60 µm. Insets are zoom-in of boxed region (first panel on left). Other insets show color deconvolutions for each of the stains (Mena, Endomucin, IBA-1). TTC = TMEM Doorway Tumor Cell. EC = Endothelial Cell. Mϕ = Macrophage. Scale bar = 15 µm. **Right** Sequential slide of tissue in Left panel, immunofluorescently stained for NR2F1 expressing tumor cells: GFP = green; NR2F1 = red; DAPI = blue. Red dashed circle encompasses the perimeter of TMEM doorway. Vertices of the orange triangles point to each constitutive cell. Red arrow points to NR2F1-positive cells. **b** Quantification showing the frequency of distances between NR2F1 positive tumor cells (red dots) or NR2F1 and Mena^INV^ double-positive tumor cells (blue dots) to the nearest TMEM doorway in the primary tumor. Data is normalized to the frequency of distances between all DAPI positive nuclei to the nearest TMEM doorway. Bar = mean. Error bars = ±SEM. *n *= 10 1–3 mm^2^ regions of interest area in four mice for NR2F1, and *n *= eight 1–3 mm^2^ regions of interest area in 4 mice for NR2F1 and Mena^INV^. For comparison between the 0 and 200 µm bins, a two-tailed unpaired t-test was used (Mena^INV^+: *p* = 0.0004; NR2F1+Mena^INV^+: *p* = 0.029). *p < 0.05. ***p < 0.001. **c** Quantification showing the frequency of distances between NR2F1^+^ tumor cells or NR2F1^+^ (red dots) and Mena^INV+^ tumor cells (blue dots) to the nearest blood vessel lacking TMEM in the primary tumor. Data is normalized to the frequency of distances between all DAPI positive nuclei to the nearest blood vessel lacking TMEM. Bar = mean. Error bars = ±SEM. *n *= nine 1–3 mm^2^ regions of interest area in four mice for NR2F1, and *n *= eight 1–3 mm^2^ regions of interest area in fourmice for NR2F1 and Mena^INV^. Source data are provided as a Source Data file.

Serial sections of primary breast tumor tissues were stained for TMEM doorways using a triple immunohistochemical stain^50,52^ (Fig. 6a, left and insets) or with GFP to identify tumor cells, NR2F1, and DAPI (Fig. 6a, right and rightmost inset). Using digital whole slide scanning and tissue alignment software (see Methods), we were able to co-register the two slides down to the single-cell level and measure the relative distance from each NR2F1-positive tumor cell to the nearest TMEM doorway. Analysis of these distances revealed an ~2.2-fold enrichment of NR2F1-positive tumor cells near TMEM doorways (0–80 µm), compared to tumor cells farther away (160-200 µm) (Fig. 6b, red curve). Interestingly, NR2F1-positive tumor cell enrichment was specifically associated with TMEM doorways, as we did not find any enrichment around blood vessels lacking TMEM doorways (Fig. 6c, red curve). These data indicate that tumor cells within the primary tumor, and in close proximity to TMEM doorways, upregulate the expression of the dormancy marker NR2F1.

Given the high level of enrichment of NR2F1 positive CTCs (Fig. 5b), and that Mena^INV^ expression is upregulated near TMEM doorways^54^, we asked whether the two proteins are co-expressed within the same cells. This is expected as intravasation is dependent upon Mena^INV^ expression^35,55^. Thus, we stained primary breast tumors for GFP, Mena^INV^, NR2F1, and TMEM doorways (Supplementary Fig. 6), and performed a similar distance analysis to that described above.

As expected, our analysis revealed an enrichment (~3.2-fold) of double Mena^INV^-positive and NR2F1-positive tumor cells at TMEM doorways (0–80 µm), compared to tumor cells farther away (160–200 µm) (Fig. 6b, blue curve). No enrichment was observed relative to blood vessels without TMEM doorways (Fig. 6c, blue curve).

Taken together, these data demonstrate that tumor cells that are destined to disseminate, become upregulated in their expression of markers of dormancy as they approach intravasation sites.

### DTCs are upregulated in their expression of proteins associated with dormancy and dissemination, and this expression is lost with metastatic growth in the lung

Given that tumor cells acquire pro-dissemination and dormancy phenotypes as they approach TMEM doorways (where they can intravasate and disseminate), we asked next whether these phenotypes are maintained as they arrive to and take residence in the secondary site of the lung. To this end, we took lung tissues from the SM model, stained them for NR2F1 and Mena^INV^ expression (Supplementary Fig. 7a), and quantified the percent of single and double-positive tumor cells in single DTCs and in small (≤10 tumor cells), medium (11–300 tumor cells), and large (≥300 tumor cells) micro-metastases (Supplementary Fig. 7b). We found that DTCs are equally likely (~25% each) to express any combination of the two markers. However, as the tumor cells begin to grow, this expression pattern is progressively lost with the number of double negative TCs growing to ~90% of the large metastases and double-positive cells accounting for ~1%. These data are consistent with our previous observation that NR2F1 expression is lost in growing metastatic lesions in both mouse models and human metastasis^21,43^ and additionally demonstrates that Mena^INV^ expression is also lost. Altogether, these data show that tumor cells destined to disseminate, acquire a pro-dissemination and dormancy phenotype in the primary tumor that is carried to the secondary site, and is lost during metastatic growth.

### Macrophages regulate the expression of NR2F1 in tumor cells

**Fig. 7:**
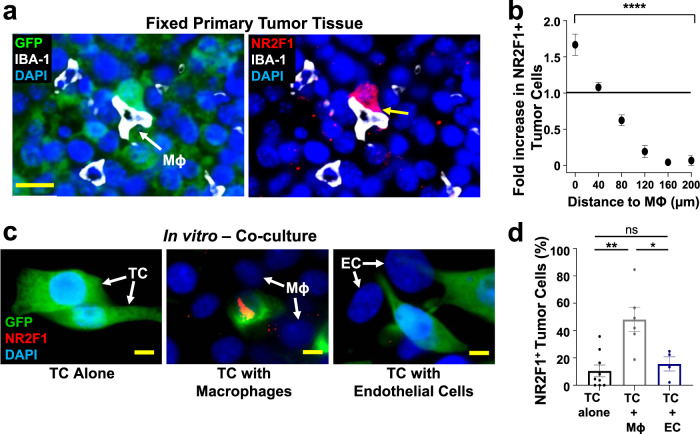
Macrophages regulate dormancy in tumor cells. **a** Representative image of triple immunofluorescently stained in E0771-GFP primary tumor tissue for tumor cells, macrophages, and NR2F1. Green = GFP; Red = NR2F1; White = IBA-1; Blue = DAPI. White arrow shows a macrophage. The yellow arrow shows the contact between an NR2F1-positive tumor cell and a macrophage. Mϕ=Macrophage. Scale bar=20 μm. **b** Quantification showing the frequency of distances between NR2F1^+^ tumor cells to the nearest macrophage in the primary tumor. Data is normalized to the frequency of distances between all DAPI^+^ nuclei to the nearest TMEM. Bar = mean. Error bars = ±SEM. *n* = 34 fields of view (551 × 316 µm^2^) in 4 animals. For comparison between the 0 and 200 µm bins a two-tailed Mann-Whitney test was used (*p* < 0.0001). ****p < 0.0001. **c** Representative immunofluorescence images of NR2F1 expression in E0771-GFP tumor cells cultured alone, in direct contact with BAC1.2F5 macrophages, or in direct contact with HUVEC endothelial cells. White arrows show macrophages or endothelial cells in direct contact with a tumor cell. Green = GFP; Red = NR2F1; Blue = DAPI. TC = Tumor Cell. Mϕ = Macrophage. EC = Endothelial Cell. Scale bar = 15 μm. **d** Percentage of NR2F1-positive tumor cells from each group in **C**. TC alone: *n* = 777 cells in 9 independent experiments; TC+Mϕ; *n* = 226 cells in 6 independent experiments, TC+EC = *n* = 359 cells in 4 independent experiments. Bar = mean. Error bars = ±SEM. For TC vs. TC+Mϕ (*p* = 0.0039), and for TC vs. TC+EC (*p* = 1), a two-tailed Kruskal-Wallis test with Dunn’s multiple comparisons adjustment was used. For TC+Mϕ vs. TC+EC (0.012), a two-tailed one-way ANOVA with Sidak’s multiple comparison adjustment was used. *p < 0.05. **p < 0.01; ns = not significant. Source data are provided as a Source Data file.

It is currently unknown whether macrophages in general, and whether macrophage-tumor cell interactions around TMEM doorways more specifically, are able to regulate NR2F1 expression on cancer cells. In order to investigate this, we studied the spatial distribution of NR2F1-positive tumor cells relative to macrophages in fixed primary breast tumors (Fig. 7a). We observed an ~2-fold enrichment of NR2F1-positive tumor cells in close proximity (0-40 µm) to macrophages in primary tumors (Fig. 7b). However, to confirm that the induction of NR2F1 is due to macrophage-tumor cell and not macrophage-endothelial cell interactions, we co-cultured tumor cells with macrophages or with endothelial cells and stained them for NR2F1. We found that NR2F1 expression is significantly increased in tumor cells co-cultured with macrophages compared to tumor cells cultured alone (48% *vs*. 10%), or co-cultured with endothelial cells (16% *vs*. 10%) (Fig. 7c, d). When co-cultured cells were separated by a 0.4 µm porous membrane, we observed a similar increase in tumor cell NR2F1 expression in the presence of macrophages, indicating that soluble factors are responsible for the induction of NR2F1 (Supplementary Fig. 8a, b).

**Fig. 8:**
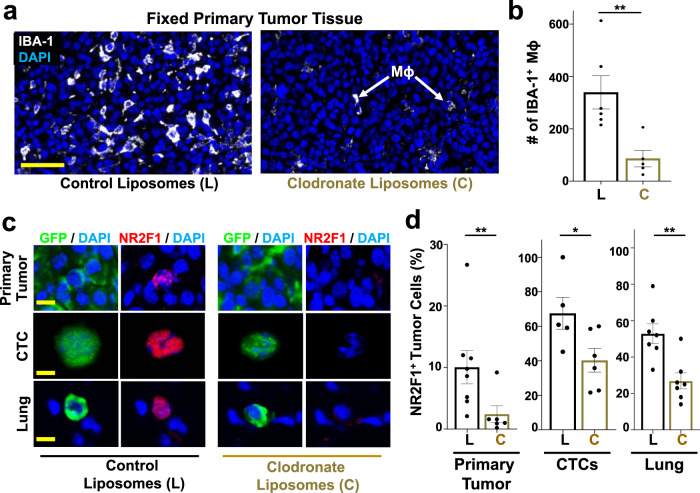
Macrophage depletion reduces dormancy in primary tumors, CTCs, and disseminating tumor cells in vivo. **a** Representative immunofluorescence images of E0771-GFP primary tumor tissues treated with either control liposomes or clodronate liposomes and stained for macrophages: IBA-1 = White; DAPI = Blue. Scale bar for Primary Tumor = 100 μm. Mϕ = Macrophage. **b** Percentage of IBA-1 positive macrophages in 10 fields of view (1088 × 629 μm^2^) in each group from Fig. 8a. Control Liposomes: *n* = 60 HPFs in 6 animals. Clodronate liposomes: *n* = 50 HPFs in 5 animals. Bar = mean. Error bars = ±SEM. Unpaired t-test (*p* = 0.0087). **p < 0.01. **c** Representative immunofluorescence images of NR2F1 expression in primary tumors, circulating tumor cells (CTCs), and disseminated tumor cells (Lung) from an E0771-GFP SM model treated with control (Left) or clodronate liposomes (Right). Green = GFP, Red = NR2F1, Blue = DAPI. Scale bar for Primary Tumor = 50 μm. Scale bar for CTCs and Lung = 15 μm. **d** Percentage of NR2F1-positive tumor cells in each group from Fig. 8c. Control Liposomes - Primary Tumor: 3048 cells in 119 fields of view (65 × 65 µm^2^) in 8 animals; CTCs: *n* = 139 cells in 5 animals; Lung: *n* = 166 cells in 7 animals. Clodronate Liposomes - Primary Tumor: *n* = 2,298 cells in 79 fields of view (65 × 65 µm^2^) in 6 animals, CTCs: *n* = 293 cells in 6 animals; Lung: *n* = 190 cells in 7 animals. For primary tumor (*p* = 0.007), a Mann-Whitney test was used. For CTCs (*p* = 0.038) and Lungs (*p* = 0.0029), unpaired t-tests were used. *p < 0.05. **p < 0.01. Source data are provided as a Source Data file.

To determine the impact that the presence or absence of macrophages, systemically, has on NR2F1 expression, we treated tumor-bearing mice (SM model) with control liposomes or clodronate liposomes to systemically deplete them of macrophages^56^. Clodronate treatment led to a significant macrophage depletion in primary tumor tissues (Fig. 8a, b, Supplementary Fig. 8c, d).

We then quantified the percentage of NR2F1-positive tumor cells in primary tumors, CTCs, and lung tissues from an E0771-GFP SM model (Fig. 8c). We found that, while the overall number of disseminated tumor cells is reduced in clodronate liposome treated animals^36,49,57^, of the cells that do disseminate, there is a significant reduction in the fraction that are positive for NR2F1 in CTCs and in disseminated tumor cells found in the lungs, as well as in the primary tumor, compared to control animals (Fig. 8d). Similar observations were made with 231-GFP cells (Supplementary Fig. 8e, f).

To rule out the possibility that clodronate treatment affects other phagocytic cell types, we analyzed dendritic cells (CD11c^+^ and IBA-1^−^) in both mice treated with liposome control and clodronate liposomes. In accordance with earlier published studies^58,59^, we observed no significant change in the number of CD11c^+^ IBA-1^−^ cells following clodronate treatment, suggesting that clodronate is specific to macrophages (Supplementary Fig. 9).

We therefore conclude that tumor associated macrophages in the primary tumor play an important role in inducing the expression of dormancy markers in primary cancer cells that are destined to disseminate.

### Expression of NR2F1 and Mena^INV^ are regulated together

Given our observation that the expression of Mena^INV^ and NR2F1 in tumor cells are both increased near TMEM doorways, we sought to determine if both proteins are mechanistically linked, or independently regulated. To this end, we stained and quantified the expression of NR2F1 in MMTV-PyMT tumors taken from wild-type and Mena knock-out mice (Supplementary Fig. 10a). We found that NR2F1 expression is significantly decreased in mice lacking expression of Mena (Supplementary Fig. 10b). This indicates that NR2F1 and Mena^INV^ are, to some extent, dependently regulated within tumor microenvironment, although we cannot conclude from these data that Mena directly regulates NR2F1. There is still a possibility that they have independent or indirect mechanisms of regulation as well.

### Systemic depletion of macrophages reduces tumor cell retention, extravasation, and survival

Given that spontaneously DTCs acquire the expression of Mena^INV^ and NR2F1 when they interact with macrophages near TMEM doorways, we sought to test whether systemic depletion of macrophages would prevent the ability of tumor cells to extravasate and survive in secondary sites, as is expected based upon earlier work^60,61^. To this end, we systemically depleted macrophages in mice bearing a primary tumor (SM model) using clodronate liposomes, and tracked the fate of DTCs during the steps of retention, extravasation, and survival in the lung (Supplementary Fig. 11a). As expected, in mice treated with control liposomes, a large percentage of E0771-GFP tumor cells were retained in the lung for the entire experimental period (Supplementary Fig. 11b). However, in macrophage-depleted mice, the number of tumor cells retained in the lung drastically decreased with only few cells remaining in the lung at the end of the experiment (5%). We further found that, while the majority of tumor cells were able to extravasate in the control mice (68%), a significantly lower proportion of cells (31%) extravasated during the same time period after arrival to the lung in the clodronate treated mice (Supplementary Fig. 11c). We further aimed to test if the depletion of macrophages would influence tumor cell survival after extravasation in the lung. To this end, we tracked each individual tumor cell longitudinally, as previously described. We found that the depletion of macrophages drastically decreased the survival of DTCs after extravasation, making them similar to EM cells (Supplementary Fig. 11d). Together, these data, along with those in Figs. 7 and 8, indicate the crucial role of primary tumor-associated macrophages in educating tumor cells for retention, extravasation, and survival in the lung, although we cannot rule out the possibility that macrophages at secondary sites might play a complementary role.

To further confirm the role of macrophages in tumor cell extravasation and survival, we used an alternative method to systemically deplete macrophages: the macrophage-fas-induced apoptosis (MaFIA)^48,62,63^. In this model, treatment with the small molecule, AP20187 (also known as the B/B homodimerizer), drastically decreases the number of macrophages systemically (Supplementary Fig. 12. Similar observations to the clodronate treatment were made with the MaFIA mice, validating the role of macrophages in tumor cell retention, extravasation, and survival (Supplementary Fig. 13).

### Macrophages condition disseminating tumor cells for enhanced retention, extravasation, and survival

To further test whether interactions between tumor cells and macrophages before dissemination would educate tumor cells to acquire the ability to be retained and survive in the lung, we co-cultured tumor cells with or without macrophages in vitro, isolated the tumor cells, injected them into the tail veins of mice, and tracked their fate. We found that ~33% of macrophage-conditioned tumor cells were retained in the lung for the entire experimental period compared to only 5% of tumor cells cultured alone (Supplementary Fig. 14a, b) indicating that tumor cell-macrophage interactions, before intravasation, can educate disseminating tumor cells for increased retention within the naïve lung.

Although this increase in retention was statistically significant, we did not observe as great an increase as was observed with SM cells. Indeed, the difference between the SM cells and the macrophage conditioned tumor cells was also statistically significant, suggesting that macrophages within the secondary site (or other variables not reproduced by the in vitro co-culture) additionally condition disseminating tumor cells for increased retention, as has been described previously^60,61^.

Similarly, we found that macrophage conditioned tumor cells survived after extravasation at rates similar to SM cells, again indicating that tumor cell-macrophage interactions, before intravasation, play a role in educating tumor cells for retention and post-extravasation survival at the secondary site. Together, these data indicate that interactions with macrophages before arrival to the secondary site increase retention and survival of disseminating tumor cells, although not to the same level as SM cells.

### The presence of a primary tumor cannot account for all SM phenotypes

Given that tumor cell-macrophage interactions before dissemination are not able to completely rescue the SM phenotype, we next asked whether the simple presence of the primary tumor also affects the ability of DTCs to be retained, extravasate, and survive in the secondary site. To address this question, we intravenously injected E0771-GFP tumor cells into mice bearing a primary tumor labeled with a different color (E0771-mCherry), and tracked the fate of the GFP labeled cells for 48 hrs (Supplementary Fig. 15a). In contrast to the near-complete clearance of EM cells injected into naïve mice, EM cells injected into mice bearing primary tumors (EM+PT cells) show a statistically significant increase in retention (8% *vs*. 37%) over 48 hrs (Supplementary Fig. 15b). However, EM+PT cells are also retained at significantly lower levels than SM cells, indicating that conditioning of the secondary site by the primary tumor is, by itself, insufficient to rescue the SM phenotype. We further found that EM+PT cells show higher levels of extravasation into the lung parenchyma, approaching those observed with SM cells (Supplementary Fig. 15c). Finally, we observed that the survival of EM+PT cells after extravasation never reached the levels of SM cells, with the difference from EM not statistically significant (Supplementary Fig. 15d).

Taken together, these results suggest that while the presence of the primary tumor only partially influences tumor cell retention and extravasation: the impact is incomplete compared to that observed with SM cells with SM enabling a unique retention and a unique survival capacity post-extravasation. Therefore, as a whole, the primary tumor’s effect on the systemic environment cannot completely account for the SM phenotypes.

## Discussion

Studying the fate of individual DTCs in an intact organ such as the lung has been a major challenge for metastasis research because, until recently, it has not been possible to follow individual DTCs in the lung over time. The result of this limitation is that it has been impossible to determine when spontaneously disseminated tumor cells had arrived to the organ, and for how long they had resided there. To overcome this limitation many studies have relied on the experimental metastasis (EM) assay where tumor cells are injected directly into the vasculature to set a time zero for the study.

As revealed in our work, a major caveat of the EM assay is the implicit assumption that the processes of education that disseminating tumor cells undergo within the primary lesion are of marginal importance for DTC fate, and that cancer cells injected as a bolus directly into the vasculature are identical in their ability to progress through the metastatic cascade.

However, it is becoming increasingly clear that the primary tumor plays an important role in determining DTC fate. For example, it is possible to find gene signatures within the primary tumor that indicate whether tumor cells are likely to disseminate^64^, and if they are likely to grow into metastases^65^, even long after dissemination^19,20^. In models where primary tumors are never excised, these can create systemic effects, preparing pre-metastatic niches in secondary sites^66^ or influencing the reaction of the immune system to disseminated tumor cells^67^. In addition to these effects, we recently determined that intratumoral microenvironments can activate programs of dormancy in DTCs^21^, which may be the unaccounted for mechanism of therapy evasion and late recurrence in patients.

This is in accord with our observation that tumor cells that spontaneously disseminate from primary tumors remain in the lung for extended periods of time compared to those that are injected directly into the vasculature. Thus, the primary tumor microenvironment and access to vasculature from these niches play a protective role for the cancer cells destined to disseminate.

It was previously proposed that the harsh conditions of the circulatory system lead to tumor cell destruction^33^, and that metastatic seeding is more likely to occur in areas with low perfusion^27^. Consistent with this, we found that the survival advantage of spontaneously metastasizing (SM) cells over injected cells may be connected to their ability to extravasate into the lung parenchyma faster, resulting in a decreased time-from-arrival-to-extravasation and a shorter exposure to the circulatory system. Clearance of EM tumor cells from the vasculature could not be a result of an adaptive immune reaction to GFP, firstly, because it has been previously shown that GFP produces no detectable in vivo immune responses in C57B6 mice^68^, and second, because any immune reaction would be expected to affect both EM and SM models equally, making EM and SM cells equally susceptible to adaptive immune clearance. Taken together, these observations indicate that neither destruction in the circulation nor extravasation are major limiting steps for disseminating tumor cells originating in a primary tumor.

Our observations show that, although the difference in overall tumor cell retention between EM and SM cells in the immunocompromised model is significant, it is not as large as that observed in a syngeneic model (Fig. 1c). This reduction could be due to the immune system. While it has not been shown that B and T cells influence DTC clearance from the lung vasculature, it has been demonstrated that NK cells play a critical role in preventing tumor cell retention in the lung^5,69,70^ and several studies have demonstrated that the activity of NK cells is higher in Nude mice compared to C57B6 mice^71,72^. It has further been shown that there is a difference in NK cell reactivity between Nude and C57B6 mice, with Nude mice having much higher levels^71,73,74^. Therefore, we propose that the observed increased clearance in the 231-GFP SM model is likely due to a higher activity of NK cells in Nude mice compared to C57B6 mice. This is an avenue that merits further investigation.

**Fig. 9:**
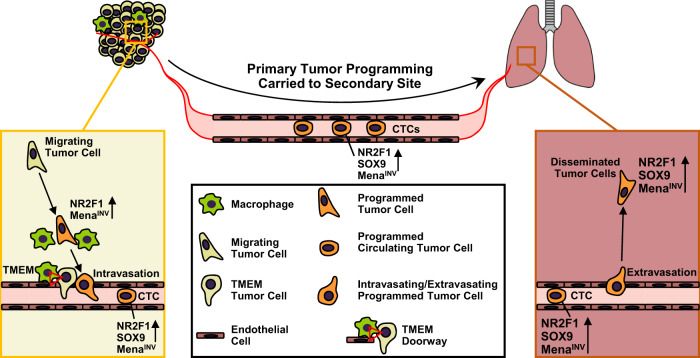
Model illustrating how the presence of a primary tumor programs disseminated tumor cells for stemness and dormancy at the secondary site. Left Panel: Within the primary tumor, migrating tumor cells are attracted to blood vessels. As they approach TMEM doorways (red triangle) on the vasculature, these tumor cells interact with macrophages and programs of dormancy (NR2F1) and invasion (Mena^INV^) are activated. Dormant cells also adopt cancer stem cell properties (SOX9). These cells then intravasate through TMEM doorways into the vasculature and become circulating tumor cells (CTCs). **Right Panel:** CTCs retain these programs at the secondary site where the invasion program (Mena^INV^) facilitates extravasation. The dormancy program expressed by these disseminated tumor cells (DTCs) keeps them as single cells.

Through gain- and loss-of-function experiments, we showed that Mena^INV^ (an isoform of the actin regulatory protein, Mena, involved in cell motility and chemotaxis^38^), is required for extravasation and is one important factor in the faster extravasation of SM cells compared to EM cells. We previously showed that expression of Mena^INV^ drives invadopodium assembly and function^35^ and is required for transendothelial migration within the primary tumor^75^, and that expression of Mena^INV^ persists in primary tumors, CTCs, and DTCs in the lung^76^. However, it was unknown whether Mena^INV^ plays a role in extravasation in the secondary site. Our current work thus indicates that Mena^INV^ is a common molecular pathway, important for many of the steps of metastasis including invasion^35,38^, intravasation^75^, and now extravasation (Fig. 9).

Given the importance of Mena^INV^ for intravasation, it may be surprising that only ~50% of DTCs in the lung show Mena^INV^ expression (Fig. 2b, Supplementary Figs. 2e and 7b). However, the expression of this protein is highly transient, with a lifetime on the order of only 2 min^37^. Thus, it is to be expected that, without a sustaining signal, much of this expression would be lost as it might not be needed for post-extravasation survival.

Our prior work has shown that Mena^INV^ is induced via macrophage-tumor cell contact, and that this induction is mediated by Notch signaling^35,77^. Though it was previously reported that no decrease in metastatic efficiency is observed after specific ablation of alveolar macrophages^61^, it is important to note that this observation was made using an EM model where the secondary site was not conditioned by the primary tumor. Further, the alveolar macrophage function may be cancer-type regulated as it was recently shown that it is required for the initial steps of lung cancer lung colonization^78^. However, further study of alveolar macrophage function is needed to rule in or out their role in SM biology

A distinguishing feature between EM and SM models is that the vast majority of SM cells do survive in the lung as solitary tumor cells, suggesting that they may be in a dormant state. Indeed, we found that, as in other studies^21^, DTCs express nuclear NR2F1, a well-established marker for dormancy used in different pre-clinical models^20,21,43,44^ as well as in the clinic for breast and prostate cancer patients^42^. The co-expression of NR2F1 (a dormancy marker) and SOX9 (a dormancy and cancer stem cell marker) preferentially in SM tumor cells suggests that these cancer cells adopt both dormant and cancer stem cell properties, a unique biology associated with the SM process.

The above data match our previous findings showing that hypoxia in the primary tumor induces NR2F1 expression in a subpopulation of tumor cells^21^. Here, we expand this work with the finding that the primary tumor induces the expression of both NR2F1 and SOX9 in disseminating tumor cells that are in the vicinity of TMEM doorways. While it is not impossible for TMEM doorways to be hypoxic (transient hypoxia has been observed adjacent to neoangiogenic vasculature, which is often only intermittently perfused^79^), we do not think that hypoxia at TMEM is a requirement for the induction. Cancer cells arriving to TMEM could be post-hypoxic as proposed by us^21^ as these cells have a more persistent directionality towards blood vessels^80^. Further, there is substantial crosstalk between the hypoxia and Notch pathways, with Notch signaling modulating hypoxia responses^81^, and hypoxia promoting the expression of Notch signaling proteins^82^.

Though NR2F1-positive and SOX9^High^ cancer cells constitute a very small percentage of the primary tumor, they both become enriched as tumor cells approach TMEM doorways, enter the bloodstream, and finally arrest in the lung. This is consistent with the fact that NR2F1 is upstream of SOX9. Our data expand this mechanistic analysis by showing that NR2F1 and Mena^INV^ are co-regulated, and that their induction is caused by interaction with tumor-associated macrophages which are enriched in a niche surrounding TMEM doorways^48,49^. Importantly, this highly disseminating and dormant phenotype is carried with DTCs to the secondary site. Taken together, our current work demonstrates that both NR2F1 and Mena^INV^ expression in tumor cells is induced by contact in the primary tumor with macrophages at TMEM doorways, similar to our recent finding that macrophages in the primary tumor program DTCs for stemness^54^.

These data offer the opportunity of establishing a new biomarker for the risk of carrying dormant DTCs (like that observed via the measurement of CTCs^42^) based upon the analysis of excised primary tumor tissue (like that for risk of systemic dissemination observed via TMEM detection^50–52^). While we have previously shown that depletion of macrophages dramatically reduces the number of tumor cells disseminating to the lung across tumor evolution^36,57,83^, it had not previously been demonstrated that macrophages are required to induce NR2F1-positivity in both CTCs in the blood, and DTCs in the lung. However, since clodronate treatment depletes macrophages systemically, we cannot definitively rule out a contribution (providing maintenance signals, for example) from bone marrow-derived- or tissue resident-lung macrophages to conclude that this effect is solely from primary tumor macrophages.

Two observations indicate that induction of the dormancy program does not occur in the circulation. The first is that a reduction of NR2F1 upon macrophage depletion occurred in both DTCs and CTCs, and the second is that there was no difference between the percentage of NR2F1-positive tumor cells in vitro (pre-injection) and in the lungs in the days following injection. Even with this evidence, we cannot rule out that tumor cells could eventually acquire and then maintain a dormancy program at the secondary site via other mechanisms (as we previously demonstrated with TGF-β2 signaling^84^).

Indeed, we cannot in general rule out that some tumor cell programming may occur at the secondary site via the influence of the primary tumor, either by the influence of previously disseminated tumor cells that create a favorable microenvironment for subsequently arriving DTCs, or via primary tumor secretions of exosomes and soluble factors^85–87^. An important question that remains to be elucidated is the molecular mechanism by which macrophages induce the expression of NR2F1 in tumor cells, but this investigation is beyond the scope of our current study.

While our technological advance has enabled us to visualize the lung vasculature over the time period we have presented, it is still impossible to visualize the lung vasculature over the longer durations so as to determine the persistence of NR2F1 or the further growth of metastases. Additionally, the arrival of cells to the lung is a stochastic event, and while cells can be detected in the lungs at earlier time points in primary tumor growth, their arrival is much more sporadic. Thus, the investigation of early dissemination faces a significant challenge in trying to attain a statistically quantifiable number of events.

In conclusion, these data indicate that spontaneously disseminating tumor cells acquire programs of dissemination, dormancy, and stemness by interacting with macrophages in the vicinity of TMEM doorways within the primary tumor, and that TMEM doorways are not only sites of tumor cell intravasation^48,53^, but are also microenvironmental niches that program a potentially lethal tumor cell population. This programming imparts to tumor cells the ability to intravasate and extravasate efficiently (via Mena^INV^ expression), to survive long-term, resist antiproliferative chemotherapy (via dormancy), and to acquire tumor initiation competency (via stemness) that can result in the formation of metastases.

Furthermore, our data reveal a link between the dissemination machinery and dormancy. This is an important link because it may explain the observation in patients and in mouse models that early DTCs are dormant and serve as founders of metastasis years after dissemination^88–91^.

## Methods

### Cell Culture

E0771-GFP medullary breast adenocarcinoma cells, originally isolated from a spontaneous mammary tumor in C57BL/6 mice, were obtained from Dr. Wakefield’s lab at the NIH, who in turn obtained them from Dr. Fengzhi Li in Dr. Enrico Mihich’s lab at Roswell Park Cancer Institute, Buffalo, NY. E0771 cells stably expressing-mCherry driven by the PGK promoter were generated with standard lentiviral transduction procedures and were FACS sorted for expression of the mCherry vector.

The MDA-MB-231 human breast cancer cell line was purchased from ATCC. The MDA-MB-231, stably expressing GFP, were generated using retroviral vectors with retroviral packaging and infection and were FACS sorted for the over-expression of each fusion protein, as described elsewhere^92^. MDA-MB-231-GFP cells over-expressing Mena^INV^ or Mena11a were generated as previously described^36^. shRNAmir-NR2F1 cells were generated by TURBO-RFP-shNR2F1-mir-encoding lentivirus infection of MDA-MB-231-GFP cells which were then selected with puromycin (2.5 µg/mL), as previously described^43^. The MDA-MB-231-GFP, MDA-MB-231-GFP-Mena^INV^, MDA-MB-231-GFP-Mena11a, and MDA-MB-231- shRNAmir-NR2F1 cell lines were cultured in DMEM (ThermoFisher, cat #12320032) media supplemented with 10% (v/v) FBS and 1% penicillin/streptomycin. The E0771-GFP and E0771-mCherry cell lines were cultured in RPMI medium 1640 (ThermoFisher, cat #12633012), media supplemented with 10% (v/v) heat-inactivated fetal bovine serum (Atlanta Biologicals, FBS-Premium Select, cat# S11550), and 1% penicillin/streptomycin (Gibco, cat #15-140-122). Tumor cell lines were used between passage 10 and 25 and their morphology and biological properties at the time of assay were checked to be consistent with those of the original vial.

BAC1.2F5 macrophages were cultured in a-MEM supplemented with 10% FBS, and 3000 U/mL of purified human recombinant CSF-1 (generously provided by Dr. Richard Stanley, Einstein College of Medicine), and, used between passage 2 and 15. Human Umbilical Vein Endothelial Cells (HUVECs) were obtained from Lonza and were cultured in EGM-2 SingleQuot Kit media (Lonza, cat #CC-3162) and used at passage 2-10. All cell lines were authenticated and were routinely tested for mycoplasma and resulted negative (Sigma LookOut Mycoplasma PCR detection kit, cat #MO0035-1KT). Authentication of E0771-GFP cells were previously authenticated as described previously^24^.

### Animals

All procedures were conducted in accordance with the National Institutes of Health regulation concerning the care and use of experimental animals and with the approval of the Einstein College of Medicine Animal Care and Use Committee (IACUC). Two different animal models were used: an immunocompetent C57BL/6 mouse model and an immunodeficient NUDE mouse model (*Foxn1*^*nu*^*/Foxn1*^*nu*^, Jackson Labs, cat #007850). Three transgenic variants of the C57BL/6 strain of mice were used for intravital imaging: (i) a VeCad-tdTomato mouse expressing the fluorescent protein tdTomato on all endothelial cells, generated by crossing B6.FVB-Tg(Cdh5-cre)7Mlia/J (Jackson Labs, cat #006137) with B6.Cg-Gt(ROSA)26Sor^tm14(CAG-tdTomato)Hze^/J (Jackson Labs, cat #007914) and (ii) a wild type C57BL6/J mouse (Jackson Labs, cat #000664); (iii) C57BL/6 MaFIA mice C57BL/6-Tg(CSF1R-EGFP-NGFR/FKBP1A/TNFRSF6)2Bck/J) (Jackson Labs, cat # 005070). Mice were bred in house, except for MaFIA mice that were obtained from the Jackson Laboratory. Only female mice between 12 and 24 weeks of age were used for experiments. VeCad-tdTomato mice were genotyped by visually checking for the presence of the tdTomato fluorescent protein in the blood of mice.

Transgenic mice expressing the Polyoma Virus Middle-T (PyMT) antigen under the control of mammary tumor virus long terminal repeat (MMTV-LTR)^93^ were bred in-house. Generation of MMTV-PyMT MENA−/− mice by crossing PyMT mice with MENA heterozygotes has been described previously^94^. All transgenic mice were checked for the presence of the transgenes via PCR analysis.

Mice were maintained in a light, humidity, and temperature-controlled environment. Specifically, the light-dark cycle was controlled with light from 6 am to 8 pm, 45–65% humidity, and 21–25 C temperature.

### Metastasis models

#### Experimental metastasis model (EM)

E0771-GFP, MDA-MB-231-GFP, MDA-MB-231-GFP-Mena^INV^, MDA-MB-231-GFP-Mena11a, or MDA-MB-231-GFP-shRNAmir-NR2F1 cell lines were prepared by trypsinizing a 10 cm confluent culture dish of tumor cells and passing them through a 40 μm cell strainer (Falcon, cat #352340) to avoid clumps. A total of 2 × 10^5^ cells was resuspended in 50 μL of sterile PBS and intravenously (iv) injected via lateral tail vein into a WHRIL-bearing mouse.

#### Spontaneous metastasis model (SM)

E0771-GFP or MDA-MB-231-GFP cells were prepared as described above. E0771-GFP cells (1 × 10^6^) were resuspended in 100 μL of sterile PBS, MDA-MB-231-GFP cells (1 × 10^6^) were resuspended in 100 μL of 20% of collagen I (BD Biosciences, cat #354234). Cells were injected in the 4th lower left mammary fat pad of the mouse. Tumor size was measured once per week using a Vernier caliper and tumor volume was calculated using the ellipsoid formula: tumor volume (mm^3^) = (width in mm)^2^ × (length in mm)/6. The maximum tumor size allowed by our animal protocol is 1,500 mm^3^. Therefore, when tumor reached a size around 1,500 mm^3^, a WHRIL was placed and imaging performed 24 h later.

### Surgery for implantation of the window for high resolution imaging of the lung (WHRIL)

The surgery for the WHRIL implantation and the Window passivation method were performed as described previously^24,95^. Briefly, mice are anesthetized, depilated (chest hair), intubated, ventilated, and secured in the left lateral decubitus position on a heated surgical platform. A pre-surgery analgesic is administered, and the skin and muscle resected from the upper left chest region. The thoracic cavity is breached and a 5 mm circular defect is cut in the chest wall exposing the lung tissue. A window frame is sutured in place and the underside adhered to lung tissue while applying positive end-expiratory pressure (PEEP). Next, a 5 mm coverslip is coated on one side with adhesive and used to seal the aperture of the window frame while adhering the exposed lung tissue to the glass and a purse-string suture is used to cinch the skin within the window frame groove. Finally, an insulin syringe is placed into the thoracic cavity through the diaphragm and used to remove any excess air and the mouse allowed to recover from anesthesia.

### Intravital Imaging

#### Procedure

Mice were anesthetized using 2% isofluorane and injected with 50 µL of 155 kDa Tetramethylrhodamine-labeled dextran (200 µg/mL) retro-orbitally for visualization of blood flow, as previously described^24,96^. Mice were inverted, placed on the microscope stage, and a fixturing plate was taped to the stage using paper tape. The animal was placed in a heated chamber maintained at physiological temperatures by a forced-air heater (WPI Inc., AirTherm ATX), during the course of imaging. Imaging was performed on a previously described, custom-built, two-laser multiphoton microscope^97^. All images were captured in 16 bit using a 25×1.05 NA objective lens and acquired with two frame averages.

#### Retention of tumor cells analysis

As summarized in Fig. 1a, for the EM model, the WHRIL was implanted in a naïve mouse and 24-h post-operation, 2 × 10^5^ E0771-GFP or MDA-MB-231-GFP tumor cells were iv-injected. Tumor cells observed trapped in the lung vasculature under the (WHRIL) were immediately recorded at the time zero (*t* = 0). Subsequently, every 8 h the lung was imaged (*t* = 8, 16, 24, 32, 40, 48, 56, and 64 h post-injection) and, using in vivo micro-cartography to return to the same imaging field^24^, the same lung vasculature was re-localized to observe and track individual tumor cells longitudinally. For the SM model, the WHRIL was implanted in a mouse bearing a tumor of *~*1,500 mm^3^ in size. 24-h post-operation, the lung was imaged to identify DTCs already present in the lung (Fig. 1a, Time Point = Pre). These cells, and any pre-existing metastatic tumors, were then excluded from further analysis. 8 h later, the lung was again imaged and any newly arrived DTCs were recorded (Fig. 1a, Time Point = 0 h). Then, similar to the EM model, the lung was imaged every 8 h (at 8, 16, 24, 32, 40, 48, 56, 64 h from the time that DTCs arrive in the lung vasculature) to track longitudinally the fate of spontaneously DTCs over a period of 64 h.

For the retention analysis, we defined a tumor cell at each time point as “retained*”* in the lung when we were able to observe the same cell during each imaging session, independent of whether the cell was intra- or extravascular. If the tumor cell was not observed at a time point, then we defined this cell as “*disappeared”*. Kaplan-Meier survival curves showing the retention of tumor cells in the lung over time were generated with GraphPad Prism.

#### Extravasation of tumor cells analysis

Tumor cells were divided into two subclasses: intravascular or extravascular, based on their location relative to the vasculature. To determine the location of a cell relative to the vasculature, the images of the vasculature at each time point (*t* = 8, 16, 24, 32, 40, 48, 56, and 64 h) were analyzed and co-registered with the corresponding prior time point using Adobe Photoshop CC 2015. The tumor cells overlapping with vasculature were considered to be intravascular. Cells not overlapping with the vasculature were considered as having extravasated. Tumor cells were excluded if their localization could not be accurately resolved. For extravascular tumor cells, we were also able to identify three different fates over time: 1) tumor cell death, 2) survival as a single and solitary tumor cell, or 3) growth into micro-metastases. We identified tumor cell death by the appearance of cellular debris (apoptotic bodies) in the field of view. We determined a tumor cell to have survived as a single tumor cell when we observed it remain a single cell in the same field of view over time. Finally, we determined a tumor cells to have formed micro-metastases when we observed cells to have increased in area by larger than that of a single cell.

#### Migration of tumor cells analysis

To track the migration of tumor cells, and to confirm that we were able to observe the same tumor cell at each imaging session, continuous time-lapse imaging of a minimum 8 h was performed to record the motility path of tumor cells in the lung vasculature. For the time-lapse imaging sessions, a tail-vein catheter was inserted to periodically provide hydration (PBS) and to allow re-administration of dextran^96^. Cell motility was manually tracked from one frame to the next using the ROI_Tracker plugin^97^. These traces were plotted in Excel (Microsoft) and used to calculate the migration paths of each cell.

#### Image processing and analysis

Image analysis was performed in ImageJ/Fiji^98^. All images presented are the raw data acquired from the microscope with minimal adjustment of brightness and contrast and with the application of our previously published blood averaging technique which removes the variation in blood serum signal created by flowing erythrocytes^24^. Time-lapse movies were assembled into Hyperstacks and any slight, residual x-y drift not eliminated by the fixturing window was removed using the HyperStackReg plugin^99^ (https://github.com/ved-sharma/HyperStackReg), which is based upon the StackReg plugin for ImageJ^100^.

### In vitro co-culture assay

For the co-culture assay, E0771-GFP tumor cells were plated either in direct contact, or in a 6-well Transwell system with BAC1.2F5 macrophages or HUVEC cells at a 1:4 ratio (20,000 tumor cells and 80,000 macrophages or HUVEC cells) for 24 h in DMEM supplemented with 10% FBS. The following day, tumor cells were stained for NR2F1 expression as described in the “Immunofluorescence of Tumor Cells Cultured in Vitro” section.

### Macrophage separation from cultured cells

E0771-GFP tumor cells were plated either alone or in direct contact with BAC1.2F5 macrophages at a 1:4 ratio (200,000 tumor cells and 800,000 macrophages) for 48 h in DMEM supplemented with 10% FBS. The following day, tumor cells and macrophages were collected and tumor cells were isolated from macrophages using MACS CD11b Microbeads (Miltenyi Biotec, cat #130-049-601), as previously described^101^. Briefly, after counting, 1 × 10^7^ cells were resuspended in 90 µL of buffer (PBS, 0.5% BSA, and 2 mM EDTA) and 10 µL of MACS CD11b Microbeads (Miltenyi Biotec). These microbeads are colloidal super-paramagnetic beads conjugated with monoclonal anti-mouse CD11b (Mac-1α) antibodies. The cells were placed at 4 °C for 15 min and then placed in a magnetic separator to separate macrophages from tumor cells. The purification of tumor cells from macrophages is in a range of 95%, as we previously described^101^. Collected tumor cells were counted immediately and intravenously injected in mice bearing a lung window.

### Immunocytochemistry staining of tumor cells in vitro

To test the baseline expression of NR2F1 or SOX9^High^ in tumor cells cultured in vitro, E0771-GFP or MDA-MB-231-GFP cells were plated with a confluence of 70–80% in a 35 mm glass-bottom µDishes (Ibidi, cat #81156) for 24 h in DMEM 10% FBS. The following day, tumor cells were washed with PBS three times, fixed in 4% (w/v) paraformaldehyde at room temperature for 15 min, permeabilized with 0.15% (v/v) Triton X-100 for 10 min, and blocked with a blocking buffer solution (10% FBS, 1% BSA, 0.0025% fish skin gelatin in PBS) at room temperature for 1 h. Then, tumor cells were incubated overnight at 4 C in the presence of primary antibodies against chicken anti-GFP (Novus, cat #NB100-1614, concentration 100 μg/mL) and rabbit anti-NR2F1 (Abcam, cat #ab181137, concentration 5 μg/mL), or rabbit anti-SOX9 (Millipore, cat #AB5535, concentration 1 μg/mL). The day after, cells were washed with PBS containing 0.05% Tween-20, and incubated for 1 h at room temperature with secondary antibodies conjugated with Alexa Fluor 488 for GFP (Invitrogen, cat #A11039, concentration 1 μg/mL) and Alexa Fluor 546 for NR2F1 or for SOX9 (Invitrogen, cat #A11034, concentration 1 μg/mL). Following three washes in PBS containing 0.05% Tween-20, cells were incubated with spectral DAPI (Akoya Biosciences, cat #SKU FP1490) for 5 min. Negative controls included incubation with PBS solution instead of the primary antibody. Fluorescence images were captured using an epi-fluorescence microscope (GE, DeltaVision) with a 60x objective and CoolSNAP HQ2 CCD camera. For image analysis, the NR2F1 channel was thresholded to just above background based upon the negative control. For SOX9^High^ the channel was thresholded so that the number of SOX9^High^ tumor cells was ~5% of the total number of tumor cells in the primary tumor, as previously published^54^. The same threshold was applied to the lung tissue analysis.

### Immunofluorescence (IF) staining of fixed tissues

In SM mice, primary tumors and lungs were collected when the tumor reached a size of ~1500 mm^3^. In EM mice, lungs were collected after 3 days post-tumor cells injection. After extraction, primary tumors and lungs were fixed in 10 mL of 10% of formalin (v/v) for 48 h. After 48 h, tissues were embedded in paraffin and then processed for immunofluorescence staining.

#### IF Staining for a single marker: NR2F1, SOX9, or Mena^INV^

Primary tumor or lung paraffin-embedded sections (4 μm) were first melted at 60 C for 1 h, deparaffinized in xylene, and rehydrated in a graded series of ethanol solutions. Antigen unmasking was performed 1 mM EDTA (pH 8.0) or 1x citrate buffer (pH 6.0) (Diagnostic BioSystems, cat #99990-096) at 97 C for 20 min in a conventional steamer. Slides were rinsed with PBS, permeabilized with 0.5% Triton X-100 in PBS for 5 min at room temperature, and incubated with the blocking buffer solution (10% FBS, 1% BSA, 0.0025% fish skin gelatin in PBS) for 1 h at room temperature. Slides were then incubated overnight at 4 C with primary antibodies against chicken anti-GFP (Novus, cat #NB100-1614, concentration 100 μg/mL) and rabbit anti-NR2F1 (Abcam, cat #ab181137, concentration 5 μg/mL), rabbit anti-SOX9 (Millipore, cat #AB5535, concentration 1 μg/mL), chicken-Mena^INV^ (generated in the Condeelis Laboratory, concentration 0.25 µg/mL), or rat-Endomucin (Santa Cruz, cat #sc-65495, concentration 2 μg/mL). For the Mena^INV^ staining, goat anti-GFP (Novus, cat #NB100-1770, concentration 10 μg/mL) was used. Slides were washed three times in PBS containing 0.05% Tween-20 and incubated with a secondary fluorescent antibody (all Invitrogen, concentration 1 μg/mL) for 1 h in the dark at room temperature. After washing, slides were incubated with spectral DAPI for 5 min and mounted with ProLong Gold antifade reagent (Life Technologies, cat #P36980). For negative controls, slides were incubated with PBS solution instead of primary antibodies. Slides were imaged on a Pannoramic 250 Flash II digital whole slide scanner (3DHistech) using a 20× 0.75NA objective lens to capture low magnification fields of view.

For quantification of fluorescence signals, high-resolution images were captured using an epi-fluorescence microscope (GE, DeltaVision) with a 100x objective, and CoolSNAP HQ2 CCD camera. Total cell numbers per high-power field (65 × 65 µm^2^, see legend) were counted and the percentages of positive or negative cells were calculated. NR2F1, Mena^INV^, and Endomucin channels were thresholded to just above background based upon the negative control.

#### IF co-staining for double markers: NR2F1 and SOX9, CD11c and IBA-1

For the NR2F1 and SOX9, or CD11c and IBA-1 co-staining, a multiplex immunofluorescence Perkin Elmer Opal 4-color Fluorescent immunohistochemistry (IHC) kit was used according to the manufacturer’s directions. After standard slide preparation as described above, slides were stained with different combinations of primary antibodies. For the NR2F1 and SOX9 co-staining, chicken anti-GFP (Novus, cat #NB100-1614, concentration 10 μg/mL) and rabbit anti-NR2F1 (Abcam, cat #ab181137, concentration 0.5 μg/mL), rabbit anti-SOX9 (Millipore, cat #AB5535, concentration 0.1 μg/mL), were mixed. For negative controls, negative controls, slides were incubated with PBS solution instead of primary antibodies.

Fluorescence images of NR2F1 and SOX9, were captured using an epi-fluorescence microscope (GE, DeltaVision) with a 100x objective and CoolSNAP HQ2 CCD camera. Total cell numbers per field of view were counted and the percentages of positive or negative cells were calculated.

For IBA-1 staining, rabbit anti-IBA-1 (Wako, cat #019-19741, concentration 0.05 μg/mL) was used. For CD11c staining, rabbit anti-CD11c (Invitrogen, cat #PA5-90208, concentration 1.5 μg/mL) was used. Slides were imaged on the Pannoramic 250 Flash II digital whole slide scanner using a 20 ×0.75NA objective lens. IBA-1 and CD11c channels were thresholded to just above background based upon the intensity of the negative controls.

CD11c positive cells were counted manually. IBA-1 positive cells were counted using ImageJ/FIJI in each field of view. A watershed segmentation was used to separate touching IBA-1 positive cells. Then, all cells with an area greater than 19 µm^2^ were counted using the “analyze particles” function. The total number of IBA-1 positive cells in 10 fields of view (1088 × 629 μm^2^) were counted.

### TMEM immunohistochemistry staining

Tumor sections were deparaffinized, as described above, and stained for TMEM doorways. TMEM stain is a triple immunohistochemical stain in which 3 antibodies are applied sequentially and developed separately with different chromogens on a Dako Autostainer, as previously published^51^. Briefly, we used an anti-pan-Mena antibody (BD, cat. #610693, concertation 5 µg/mL) to detect invasive tumor cells, an anti-IBA-1 antibody (Wako, cat. #019-19741, concentration 0.167 µg/mL) to detect macrophages, and an anti-endomucin (Santa Cruz, cat #sc-65495, concentration 0.67 µg/mL) to detect the blood vasculature. TMEM doorways in the E0771-primary tumor were identified manually by a pathologist.

### TMEM vs. NR2F1 or NR2F1- and MenaINV-positive tumor cells distance analysis

Sequential sections from primary E0771-GFP tumors were stained on one slide for TMEM IHC (which stains for tumor cells with Mena, macrophages with IBA-1 and blood vessels with endomucin), as described above, and with IF for GFP (to detect tumor cells) and NR2F1 or GFP, NR2F1, and Mena^INV^ (as described in the “Immunofluorescence Staining” section) on the sequential slide. Manual identification of TMEM doorways was performed by a pathologist. TMEM IHC and IF images were aligned in ImageJ using the Landmark Correspondences plugin. A series of previously published ImageJ macros^54^ were used to calculate the distance of each NR2F1 or NR2F1/Mena^INV^ double-positive tumor cell in the field to its nearest TMEM doorway or to its nearest blood vessel lacking TMEM doorways. The distance of NR2F1 or NR2F1/Mena^INV^ double-positive tumor cells to TMEM doorways was calculated in reference to TMEM doorways (Fig. 6). The boundaries of TMEM doorways were defined by a region of interest (ROI) dilated by 60 µm from the TMEM doorway macrophage. Cells that were inside or touching this ROI were considered to be at a distance of 0 µm from the TMEM doorway. Distance histograms were normalized to the distances between all tumor cells in the field (DAPI staining) and the nearest TMEM doorway or the nearest blood vessel without TMEM doorways. Similarly, distance analyses between NR2F1 or NR2F1/Mena^INV^ double-positive tumor cells and their nearest blood vessel without TMEM doorways was done by thresholding the blood vessel channel and removing blood vessels, which contained TMEM doorways. Distance histograms were analyzed and plotted in GraphPad Prism.

### Macrophage Depletion using Clodronate Liposomes

Tumor bearing mice were treated for 7 days with either 200 µL of PBS liposomes (injected intraperitoneally), or with 200 µL Clodronate liposomes (also injected intraperitoneally) (Encapsula Nano Sciences, cat. #CLD-8901). Twenty-four hours after the first injection of clodronate liposomes, the lung window was implanted and the fate of disseminated tumor cells was followed as described in the “Intravital Imaging*”* section. Primary tumors and lungs were collected, fixed as described above and paraffin-embedded sections were stained for macrophages or NR2F1 expression as described in the “Immunofluorescence Staining” section.

### Macrophage Depletion with B/B Homodimerizer in MaFIA Mice

Tumor bearing mice were treated for 7 days with either 100 µL of 10 mg/kg B/B homodimerizer (Clontech, cat #AP20187; diluted in 4% ethanol, 10% PEG-400, and 1.7% Tween-20) or vehicle control by intraperitoneal injection daily for 7 days. Twenty-four hours after the first injection of B/B homodimerizer, the lung window was implanted and the fate of disseminated tumor cells was followed as described above. In addition to the suicide gene, MaFIA mice also express GFP within all cells of the myeloid lineage. Despite our tumor cells also expressing GFP, we were able to distinguish these two cell types in our imaging. In the case of B/B treatment, these myeloid cells were eliminated, so the only GFP signal remaining was from tumor cells. In the case of control treatment, GFP-tumor cells were distinguishable by being morphologically larger than macrophages, and most importantly, by having a much brighter GFP signal than the macrophages. Thus, the signal from macrophages could be effectively eliminated by adjusting the microscope’s GFP channel detector so that only tumor cells were visible.

Primary tumors were collected, fixed, embedded in paraffin, and stained for macrophages as described above.

### NR2F1 Knockdown

#### Extravasation Analysis

2 × 10^5^ MDA-MB-231-GFP-shRNAmir-NR2F1 cells, treated with or without doxocycline, were resuspended in 50 μL of sterile PBS and intravenously injected via the lateral tail vein into nude mice bearing a WHRIL. Tumor cells trapped in the lung vasculature under the WHRIL were immediately recorded at the time zero (*t* = 0). Subsequently, every 8 h the lung was imaged for a period of 24 h and the fate of tumor cells was identified using microcartography, as described above.

#### Quantification of Micrometastatic Foci in the Lung

For experimental metastasis, 2 × 10^5^ MDA-MB-231-GFP-shRNAmir-NR2F1 cells were resuspended in 50 μL of sterile PBS and intravenously (iv) injected via the lateral tail vein into nude mice. In the doxocycline-induced knockdown of NR2F1 studies, mice were injected i.p. with 25 mg/kg of doxycycline or vehicle every 48 h, as previously described^43^.

For spontaneous metastasis, 1×10^6^ MDA-MB-231-GFP-shRNAmir-NR2F1 cells were resuspended in 100 μL of 20% of collagen I and injected in the 4th lower left mammary fat pad of nude mice. When the tumor reached a size around 1,500 mm^3^, mice were randomly divided in two groups, and treated i.p. with 25 mg/kg of doxycycline or vehicle every 48 h for 4 or 8 days, as previously described^43^.

In both EM and SM models, after the termination of the Doxocycline treatment, lungs were harvested at different time points, fixed in 10% formalin, and embedded in paraffin. Two sections for each lung were cut, each 50 µm apart, and stained for GFP and DAPI. Metastatic foci containing >5 tumor cells were counted in the entire lung tissue section in both slides. Counts were normalized to the tissue area.

### Quantification of NR2F1 Intensity in PyMT Tissues

Sections from primary MMTV-PyMT and Mena knock-out tumors were stained for PyMT and NR2F1, as described in the “Immunofluorescence Staining” section. For PyMT detection, rat anti-PyMT (Novus, cat #NB100-2749, concentration 0.01 μg/mL) was used. For NR2F1 detection, a rabbit anti-NR2F1 (Abcam, cat #ab181137, concentration 0.5 μg/mL) was used.

Fluorescence images were captured using an epi-fluorescence microscope (GE, DeltaVision) with a 100x objective, and CoolSNAP HQ2 CCD camera. Given the heterogeneous expression of NR2F1 in PyMT tissues, an analysis where NR2F1 positive or negative tumor cells were counted was not possible. Therefore, the intensity of the nuclear expression in each tumor cell was quantified. For this analysis, first a mask of nuclei was generated using the program cellpose (https://www.cellpose.org/). Then, the intensity of NR2F1 in each nucleus was measured using ImageJ/Fiji.

### Circulating Tumor Cells Staining

Mice bearing a primary tumor of ~1000 mm^3^ size were anesthetized with isoflurane and about 1 mL of blood was drawn from the right heart ventricle using 25 G needles coated with heparin. Erythrocytes were lysed using 10 mL of 1x RBC lysis buffer (eBioscience, cat #00-4333-57) for 10 min at room temperature. The samples were centrifuged at 200 g for 5 min, cells were reconstituted in 10 mL of DMEM supplemented with 10% FBS, plated in a 35 mm glass-bottom µDishes (Ibidi, cat #81156), and allowed to adhere overnight. The following day, tumor cells were stained using antibodies against chicken anti-GFP (Novus, cat #NB100-1614, concentration 10 μg/mL) and rabbit (Invitrogen, cat #A16024, concentration 1 μg/mL), goat anti-SOX9 (RD, cat #AF3075, concentration 1 µg/mL), as described in the “Immunocytochemistry Staining of Tumor Cells in vitro” section. Fluorescence images were captured using an epi-fluorescence microscope (GE, DeltaVision) with a 60x objective and CoolSNAP HQ2 CCD camera. For image analysis, the NR2F1 and SOX9^High^ were analyzed as described above.

### Western Blot

Western blot analysis was performed using standard protocols as previously described^102^. Antibodies chicken anti-Mena^INV^ (generated in the Condeelis Laboratory, concentration 0.25 µg/mL), rabbit anti-Mena11a (generated in the Condeelis Laboratory, concentration 1 mg/mL), rabbit anti-NR2F1 (Abcam, cat #ab181137, concentration 0.5 μg/mL) and mouse anti-β-actin (Sigma, cat #A5441, 1 μg/mL) were used. Images for immunoblotting have been cropped for presentation. Full-size images are presented in Supplementary Figs. 16 and 17.

### RT–PCR and qPCR

RT–PCR and qPCR were performed as described previously^75^.

NR2F1 human forward primer: 50-GCCTCAAAGCCATCGTGCTG-30.

NR2F1 human reverse primer: 50-CCTCACGTACTCCTCCAGTG-30.

### Statistical Analysis

All statistical analyses were carried out using GraphPad Prism v9 or SPSS v24. Data are expressed as mean ± standard error of the mean (S.E.M). Unless otherwise specified in the figure legends, statistical significance between groups was determined using unpaired two-tailed Student’s t-tests for normally distributed data (checked with the Shapiro-Wilk test) and with Mann-Whitney or Kruskal-Willis tests for non-normally distributed data. Differences were considered significant for *p* < 0.05. All in vivo and in vitro experiments were independently repeated and included at the least three biologically independent samples, as indicated in the figure legends. Mice that died during intravital imaging sessions or the fate of disseminated tumor cells which could not followed for the entire experimental design, were removed from the study or marked as censored.

### Reporting Summary

Further information on research design is available in the Nature Research Reporting Summary linked to this article.

## Supplementary information

Supplementary Information

Supplementary Movie 1

Supplementary Movie 2

Supplementary Movie 3

Description of Additional Supplementary Files

Reporting Summary

## Data Availability

Source data are provided with this paper. The authors declare that all data supporting the findings of this study are available within the Article, Supplementary Information or Source Data file. Source data are provided with this paper.

## Source data

Source data file
