# Supplementary material for: Primary tumor associated macrophages activate programs of invasion and dormancy in disseminating tumor cells
Source: Nat Commun. 2022 Feb 2;13:626. doi: 10.1038/s41467-022-28076-3 (PMC8811052; doi:10.1038/s41467-022-28076-3)
Supplement: Supplementary file 1 — Supplementary Information [file 41467_2022_28076_MOESM1_ESM.pdf]

Primary tumor associated macrophages activate programs of invasion and dormancy in disseminating tumor cells.

Lucia Borriello[1,2], Anouchka Coste[1,3], Brian Traub[1,3], Ved P. Sharma[1,2,4], George S. Karagiannis[2,5], Yu Lin[1,2], Yarong Wang[1,2], Xianjun Ye[1,2,4], Camille L. Duran[1,2], Xiaoming Chen[1,2,4], Madeline Friedman[1], Maria Soledad Sosa[6], Dan Sun[7,8], Erica Dalla[9], Deepak K. Singh[7,8], Maja H. Oktay[1,2,4,10], Julio A. Aguirre-Ghiso*[,2,4,7,8], John S. Condeelis*[,1,2,3,4], and David Entenberg*[,1,2,4,8,10]

[1] Department of Anatomy and Structural Biology, Albert Einstein College of Medicine / Montefiore Medical Center, Bronx, NY, USA
[2] Gruss-Lipper Biophotonics Center, Albert Einstein College of Medicine / Montefiore Medical Center, Bronx, NY, USA
[3] Department of Surgery, Albert Einstein College of Medicine / Montefiore Medical Center, Bronx, NY, USA
[4] Integrated Imaging Program, Albert Einstein College of Medicine / Montefiore Medical Center, Bronx, NY, USA
[5] Department of Microbiology and Immunology, Albert Einstein College of Medicine / Montefiore Medical Center, Bronx, NY, USA
[6] Department of Pharmacological Sciences, Icahn School of Medicine at Mount Sinai, New York, NY, USA
[7] Department of Cell Biology, Albert Einstein College of Medicine / Montefiore Medical Center, 1300 Morris Park Avenue, Bronx, NY 10461, USA
[8] Cancer Dormancy and Tumor Microenvironment Institute, Einstein Cancer Center, Albert Einstein College of Medicine/ Montefiore Medical Center, 1300 Morris Park Avenue, Bronx, NY 10461, USA
[9] Division of Hematology and Oncology, Tisch Cancer Institute, Icahn School of Medicine at Mount Sinai, New York, NY, USA
[10] Department of Pathology, Einstein College of Medicine / Montefiore Medical Center, Bronx, NY, USA

*** Corresponding Authors:**
David Entenberg: david.entenberg@einsteinmed.edu
John Condeelis: john.condeelis@einsteinmed.edu
Julio Aguirre-Ghiso: julio.aguirre-ghiso@einsteinmed.edu

33 SUPPLEMENTARY FIGURE LEGENDS

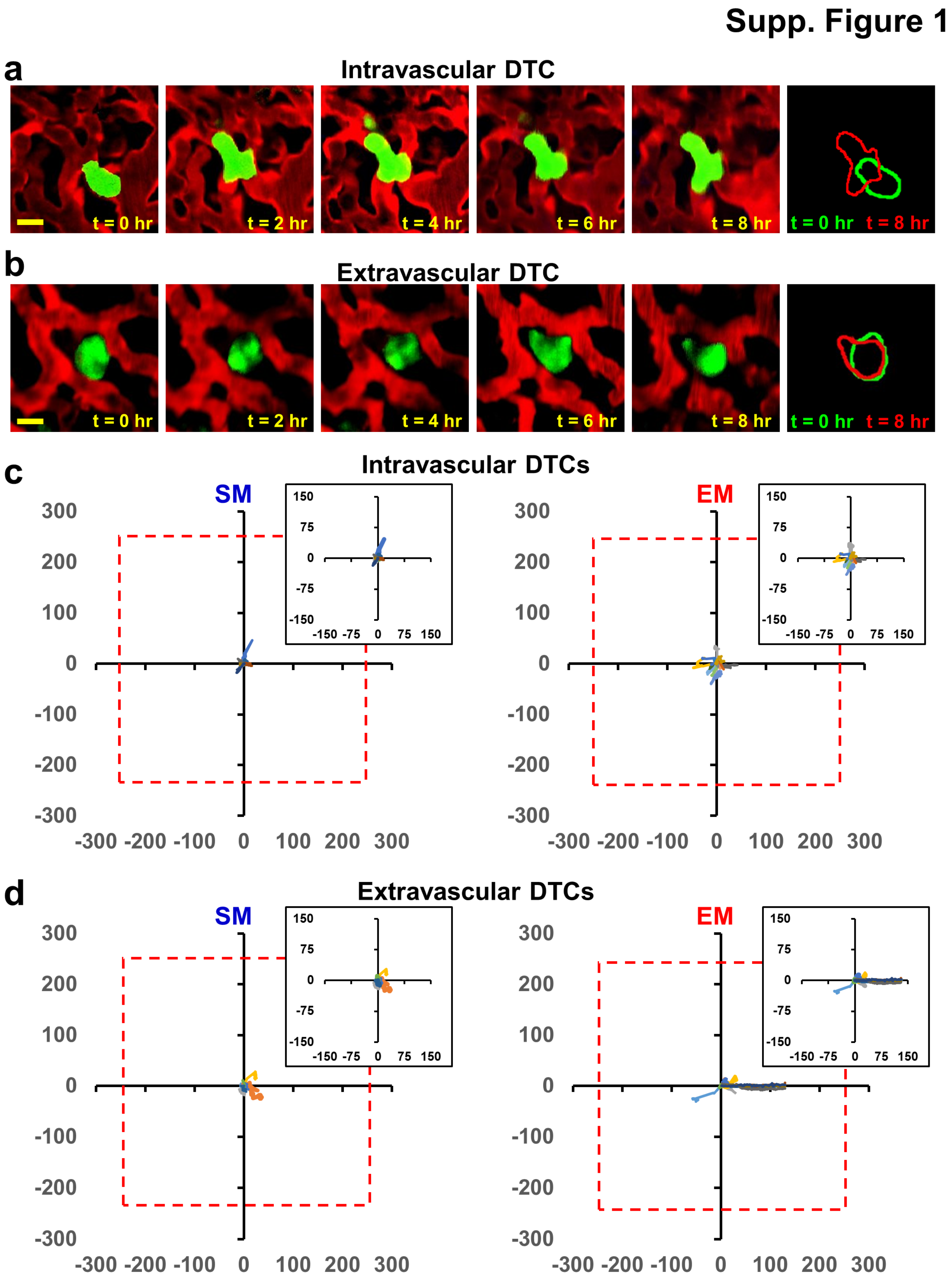

34

Supplementary Figure 1: Disseminated Tumor Cells Remain within an Imaging Field of View throughout an 8 hr Period.

a: Representative intravital microscopy images showing intravascular disseminated tumor cells at different time points spanning 8 hrs. Red=tdTomato labeled endothelial cells and 155 kDa Tetramethylrhodamine dextran labeled blood serum, Green=GFP labeled tumor cells. **Rightmost Panel:** Outlines of the tumor cell at t=0 (green) and t=8 hr (red) showing net displacement. Scale bar=15 μm.

b: Representative intravital microscopy images showing extravascular disseminated tumor cells at different time points spanning 8 hrs. Red=tdTomato labeled endothelial cells and 155 kDa Tetramethylrhodamine dextran labeled blood serum, Green=GFP labeled tumor cells. **Rightmost Panel:** Outlines of the tumor cell at t=0 (green) and t=8 hr (red) showing net displacement. Scale bar=15 μm.

c: Traces tracking the migration of intravascular E0771-GFP cells within SM (left) and EM (right) models over an 8 hr period of time. Each tracked tumor cell is represented in a plot with the initial position (t=0 hrs) translated to the origin so as to provide an overview of the migration path of each cell. Red dashed box indicates a full field of view in the microscope (512x512 μm^2). Insets are zoom-ins of the central 150 μm. Average cell velocities: SM=1.2 ± 0.1 μm/hr; EM=1.7 ± 0.1 μm/hr; mean ± SEM. SM: n=11 tumor cells in 4 mice. EM: n=22 tumor cells in 2 mice.

d: Traces tracking the migration of extravascular E0771-GFP cells within SM (left) and EM (right) models over an 8 hr period of time. Each tracked tumor cell is represented in a plot with the initial position (t=0 hrs) translated to the origin so as to provide an overview of the migration path of each cell. Red dashed box indicates a full field of view in the microscope (512x512 μm^2). Insets are zoom-in of the central 150 μm. Average cell velocities: SM=2.0 ± 0.2 μm/hr; EM =1.8 ± 0.2 μm/hr mean ± SEM. SM: n=7 tumor cells in 4 mice. EM: n=11 tumor cells in 3 mice. Source data are provided as a Source Data file.

Supp. Figure 2

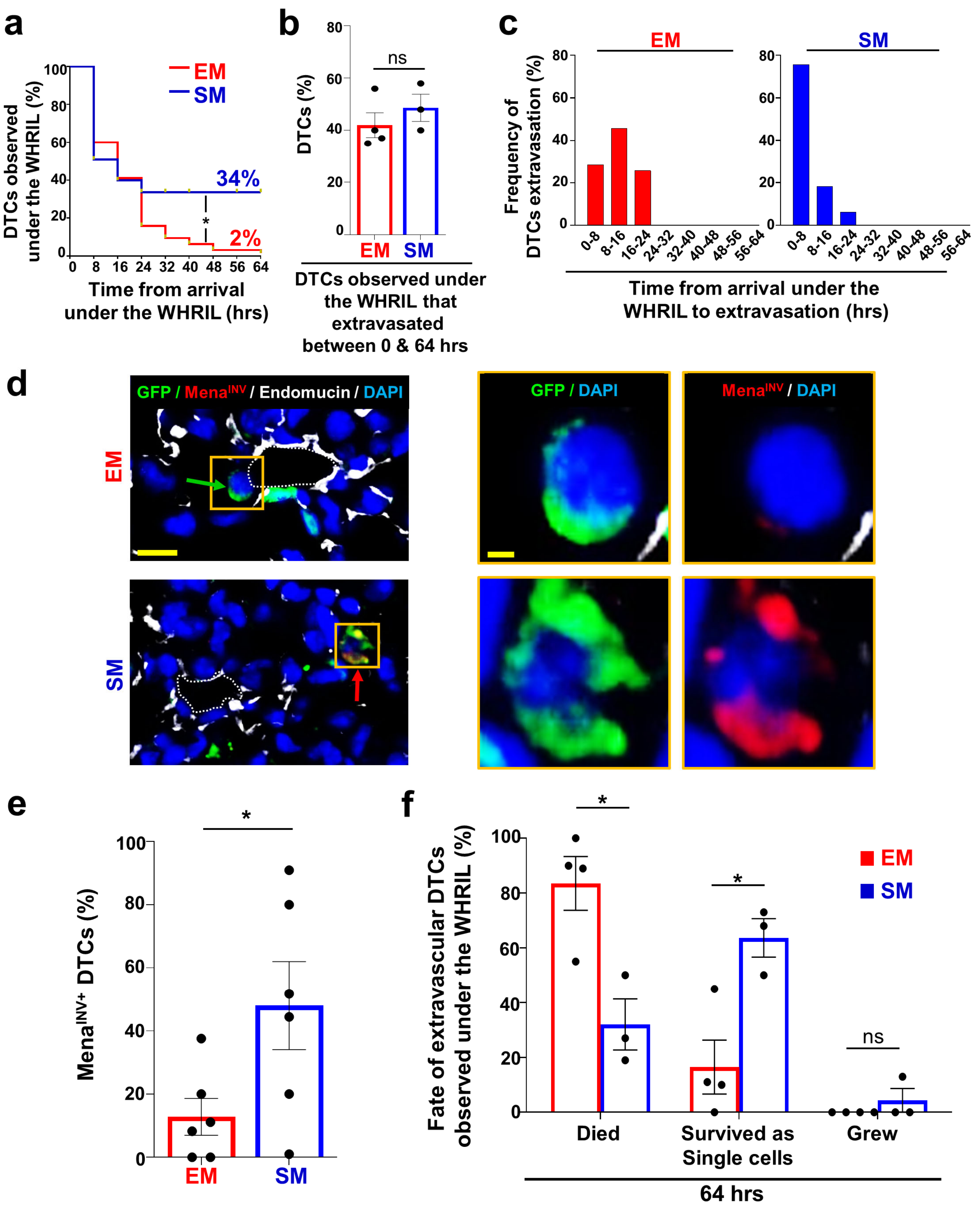

57

Supplementary Figure 2: Tumor Cells that Spontaneously Disseminate from the Primary Tumor to the Lung have a Drastically Increased Metastatic Efficiency Compared to Intravenously Injected Tumor Cells

a: Kaplan-Meier survival curves showing the percentage of 231-GFP disseminated tumor cells observed under the WHRIL at each 8 hr time point over a period of 64 hours. EM: n=95 tumor cells analyzed in 4 mice. SM: n=67 tumor cells analyzed in 3 mice. Log-rank (Mantel-Cox) test (p=0.023). *=p<.05.

b: Percentage of 231-GFP EM and SM tumor cells observed under the WHRIL that extravasated between 0 and 64 hrs after arrival. EM: n=37 tumor cells in 4 mice. SM: n=33 tumor cells in 3 mice. Bar=mean. Error bars=±SEM. Two-tailed unpaired t-test (p=0.39). ns=not significant.

c: Quantification of the time from arrival under the WHRIL to extravasation into the lung parenchyma for each 231-GFP EM and SM tumor cell. **Left:** EM: n=35 tumor cells in 4 mice. **Right:** SM: n=33 tumor cells in 3 mice.

d: Left: Representative immunofluorescence images of $Mena^{INV}$ expression in extravascular 231-GFP tumor cells in the lung of an EM model (**top**) and an SM model (bottom). Green arrow: $Mena^{INV}$ negative tumor cell. Red arrow: $Mena^{INV}$ positive tumor cell. Scale bar=50 μm. **Right:** Zoomed in view of yellow boxed area of a disseminated tumor cell in both models. Green=GFP, Red=$Mena^{INV}$, White=endomucin, Blue=DAPI. Scale bar=10 μm.

e: Quantification of extravascular $Mena^{INV}$ positive disseminated tumor cells in the lung of each group from D. EM: n=58 cells in 6 animals. SM: n=85 cells in 6 animals. Bar=mean. Error bars=±SEM. Two-tailed unpaired t-test (p=0.045). *=p<0.05.

f: Percentage of extravascular 231-GFP disseminated tumor cells that died, survived, or grew after extravasation in EM and SM models 64 hrs after arrival to the lung vasculature. Bar=mean. Error bars=±SEM. EM: n=35 tumor cells in 4 mice. SM: n=33 tumor cells in 3 mice. For the Died and Survived columns, a two-tailed unpaired t-test was used (p=0.014 and p=0.015, respectively). For the Grew columns, a two-tailed Mann-Whitney test was used (p=0.43). *=p<0.05; ns=not significant. Source data are provided as a Source Data file.

Supp. Figure 3

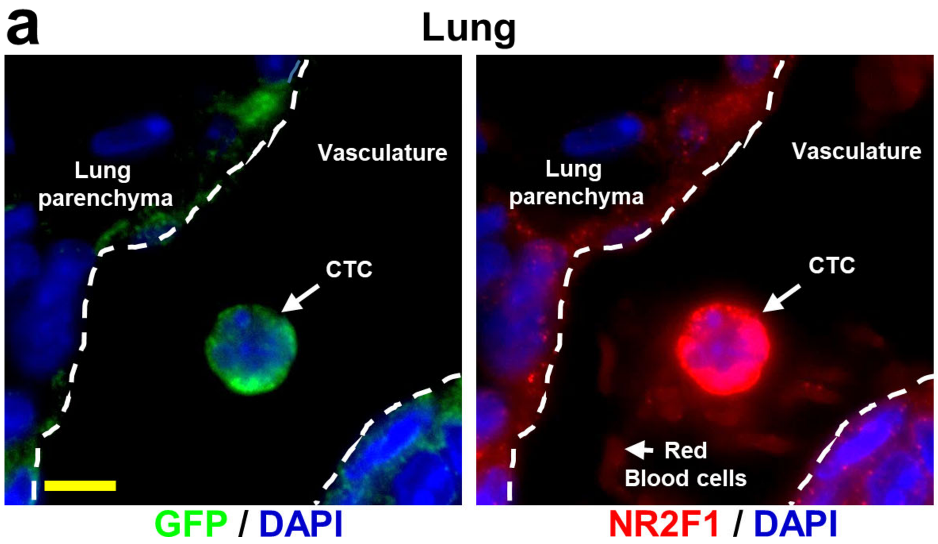

Supplementary Figure 3: Spontaneously Circulating Tumor Cells Are Positive for NR2F1 When Arriving in the Lung Vasculature

a: Representative immunofluorescence image of NR2F1 expression in circulating tumor cells in lung vasculature from a E0771-GFP SM model. n=1. **Left:** GFP channel. **Right:** NR2F1 channel. Green=GFP, Red=NR2F1, Blue=DAPI. Dotted line indicates the boundary of the vasculature. CTC=Circulating Tumor Cell. Scale bar=10 μm.

Supp. Figure 4

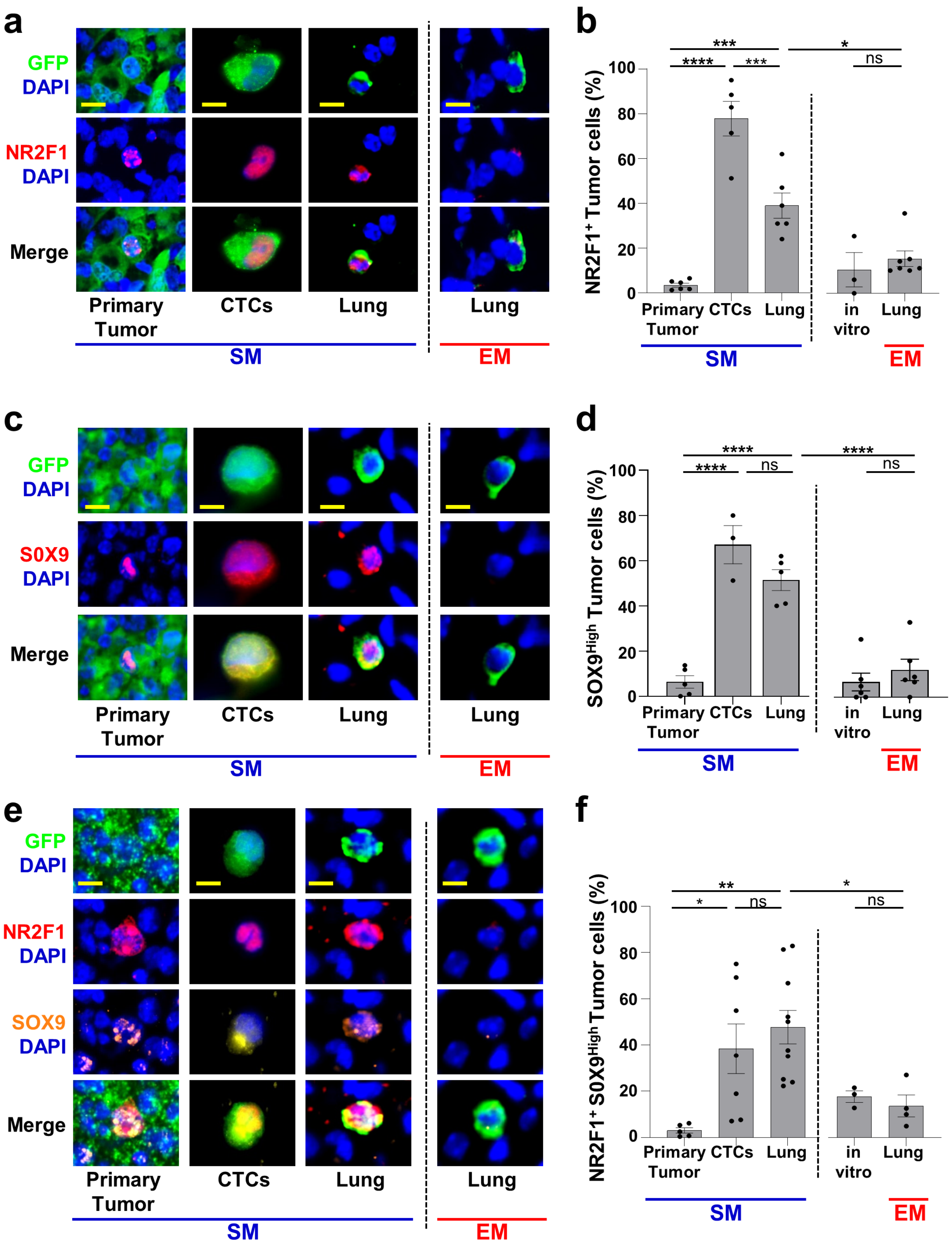

91

Supplementary Figure 4: Spontaneously Metastasizing Tumor Cells Are More Frequently Positive for Dormancy and Stem-like Markers Compared to Intravenously Injected Tumor Cells

a: Representative immunofluorescence images of NR2F1 expression in primary tumors, circulating tumor cells (CTCs), and disseminated tumor cells (Lung) from a 231-GFP SM model (**Left**) and in disseminated tumor cells (Lung) from an EM model (**Right**). Green=GFP, Red=NR2F1, Blue=DAPI. Scale bar for Primary Tumor=50 µm. Scale bar for CTCs and Lung=15 µm.

b: Percentage of NR2F1-positive and negative tumor cells in each group in Supplemental Figure 4a. Primary Tumor: n=2,729 cells in 100 fields of view (65x65 μm^2) in 6 animals; CTCs: n=166 cells in 5 animals; SM Lung: n=113 cells in 6 animals; In vitro: n= 261 cells in 3 independent experiments. EM Lung: n=133 cells in 7 animals. Bar=mean. Error bars=±SEM. For PT vs. CTC (p<0.0001), PT vs. Lung SM (p=0.0005), and CTC vs. Lung SM (p=0.0003) a two-tailed one-way ANOVA test with Sidak's multiple comparisons adjustment was used. For in vitro *vs.* Lung EM (p=0.99) and Lung SM *vs.* Lung EM (p=0.036), a two-tailed Kruskal-Wallis test with Dunn's multiple comparisons adjustment as used. *=p<0.05. **=p<0.01. ***=p<0.001. ****=p<0.0001. ns=not significant.

c: Representative immunofluorescence images of SOX9 expression in primary tumors, circulating tumor cells (CTCs), and disseminated tumor cells (Lung) from a 231-GFP SM model (**Left**) and in disseminated tumor cells (Lung) from an EM model (**Right**). Green=GFP, Red=SOX9, Blue=DAPI. Scale bar for Primary Tumor=50 µm. Scale bar for CTCs and Lung=15 µm.

d: Percentage of $SOX9^{High}$ tumor cells from each group in Supplemental Figure 4c. Primary Tumor: n=2,643 cells in 100 fields of view (65x65 μm^2) in 5 animals; CTCs: n=93 cells 3 animals, SM Lung: n=93 cells in 5 animals; EM Lung: n=99 cells in 6 animals. In vitro: n=576 cells in 6 independent experiments. Bar=mean. Error bars=±SEM. For PT *vs*. CTC (p<0.0001), PT *vs.* Lung SM (p<0.0001), CTC *vs.* Lung SM (p=0.22), and Lung SM *vs.* Lung EM (p<0.0001), a two-tailed ANOVA test with Sidak's multiple comparisons adjustment was used. For in vitro *vs.* Lung EM (p=0.31), a Mann-Whitney Test was used. ****=p<0.0001. ns=not significant.

e: Representative images of triple immunofluorescence staining for GFP, NR2F1, and $SOX9^{High}$ expression in primary tumors, circulating tumor cells (CTCs), and disseminated tumor cells (Lung) from a 231-GFP SM model (**Left**) and in disseminated tumor cells (Lung) from an EM model (**Right**).
Green=GFP; Red=NR2F1; Orange=SOX9; Blue=DAPI. Scale bar for Primary Tumor=50 µm. Scale bar for CTCs and Lung=15 µm.

f: Percentage of double positive (NR2F1-positive and $SOX9^{High}$) tumor cells from each group in Supplemental Figure 4e. Primary Tumor: n=2,140 cells in 95 fields of view (65x65 μm^2) in 5 animals; CTCs: n=199 cells in 7 animals; SM Lung: n=242 cells in 10 animals; In vitro: n=320 cells in 3 independent experiments. EM Lung: n=90 cells in 4 animals. Bar=mean. Error bars=±SEM.
For all comparisons, a two-tailed one-way ANOVA test with Sidak's multiple comparison adjustment was used. PT *vs*. CTC: p=0.034), PT *vs.* Lung SM (p=0.0027), CTC *vs.* Lung SM (p=0.89), Lung SM *vs.* Lung

EM (p=0.046), and in vitro *vs.* Lung EM (p=1.00). *=p<0.05; **=p<0.01; ns=not significant. Source data are provided as a Source Data file.

Supp. Figure 5

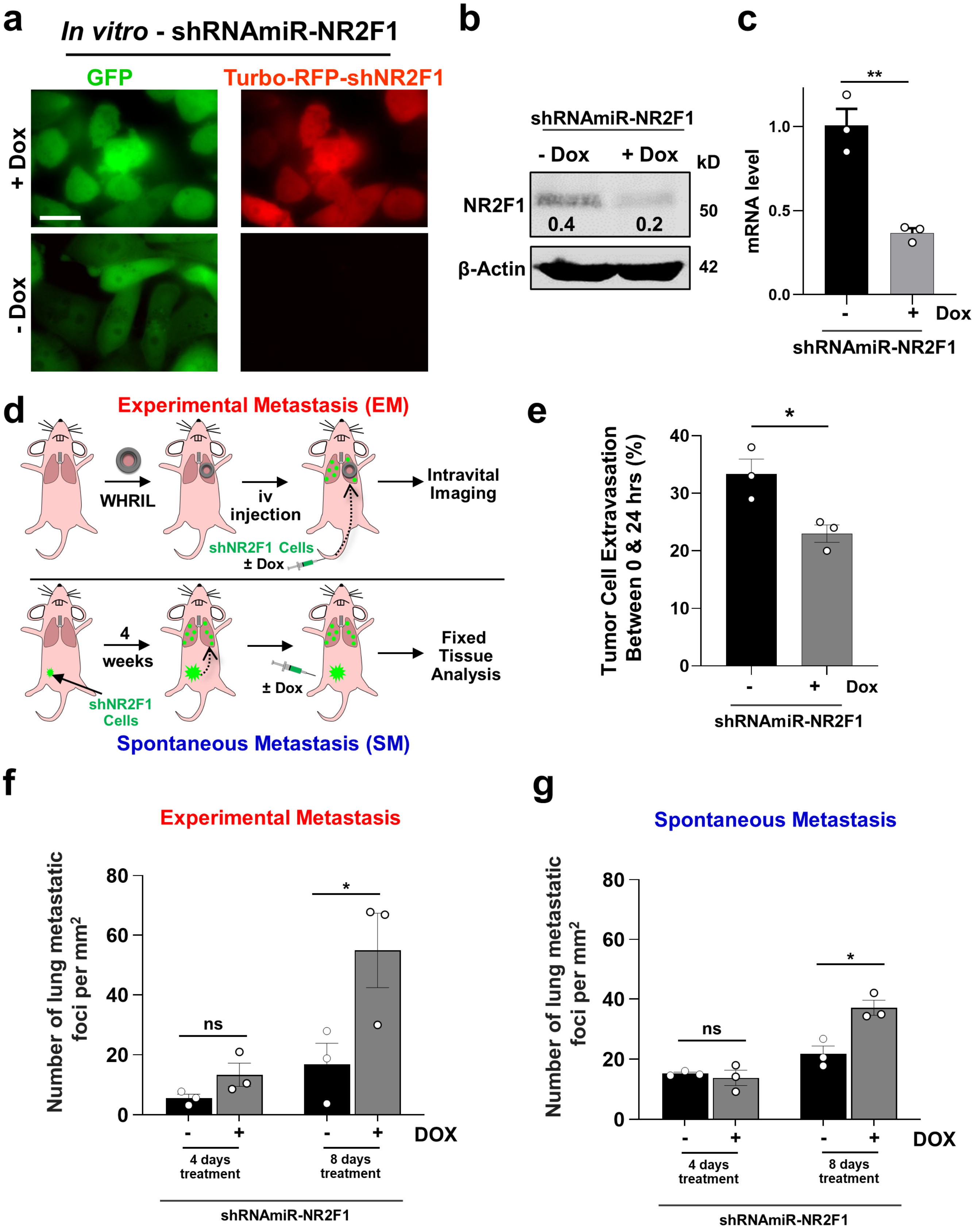

130

Supplementary Figure 5: *In Vivo* Regulation of the Fate of DTCs by NR2F1.

a: Representative images of MDA-MB-231-GFP-shRNAmir-NR2F1 cells cultured *in vitro* with, or without, doxycycline (DOX, 0.5 μg/mL). Green=GFP; Red=doxycycline shRNAmir-NR2F1. Scale bar 20 μm.

b: Western blot of MDA-MB-231-GFP-shRNAmir-NR2F1 cells treated with or without doxycycline (DOX, 0.5 μg/ml). β-Actin was used as a loading control.

c: qPCR data for NR2F1 from MDA-MB-231-GFP-shRNAmir-NR2F1 cells treated with or without doxycycline (DOX, 0.5 μg/ml). n=3 RNA replicates per condition. Two-tailed unpaired t-test (p=0.0034). **=p<0.01.

d: Outline of the experimental design for knocking down NR2F1 in 231-GFP-shRNAmir-NR2F1 tumor cells nude mice with doxycycline to determine the impact of NR2F1 expression on tumor cell extravasation and growth.

e: Percentage of MDA-MB-231-GFP-shRNAmir-NR2F1 cells treated with or without doxycycline that extravasated between 0 and 24 hrs after iv injection. Without Dox: n=47 cells in 3 mice. With Dox: n=51 tumor cells in 3 mice. Bar=mean. Error bars=±SEM. Two-tailed unpaired t-test (p=0.027). *=p<0.05.

f: Number of metastatic foci (≥5 tumor cells) counted in the lungs of nude mice intravenously injected with MDA-MB-231-GFP-shRNAmir-NR2F1 tumor cells and treated with or without doxycycline. Mice were treated with or without doxycycline (25 mg/kg) every other day for the entire experimental design. Without Dox: n=3 mice. With Dox: n=3 mice. Error bars =±SEM. For 4 days, a two-tailed unpaired t-test was used (p=0.13). For 8 days, a Kruskal-Wallis test was used (p=0.05). *=p<0.05; ns=not significant.

g: Number of metastatic foci (≥5 tumor cells) counted in the lungs of mice bearing a primary tumor and treated with or without doxycycline (25 mg/kg) every other day for the entire experimental design. Without Dox n=3 mice. With Dox: n=3. Error bars =±SEM. For both 4 days (p=0.62) and 8 days (p=0.014), a two-tailed unpaired t-test was used. *=p<0.05; ns=not significant. Source data are provided as a Source Data file.

Supp. Figure 6

155 **a**

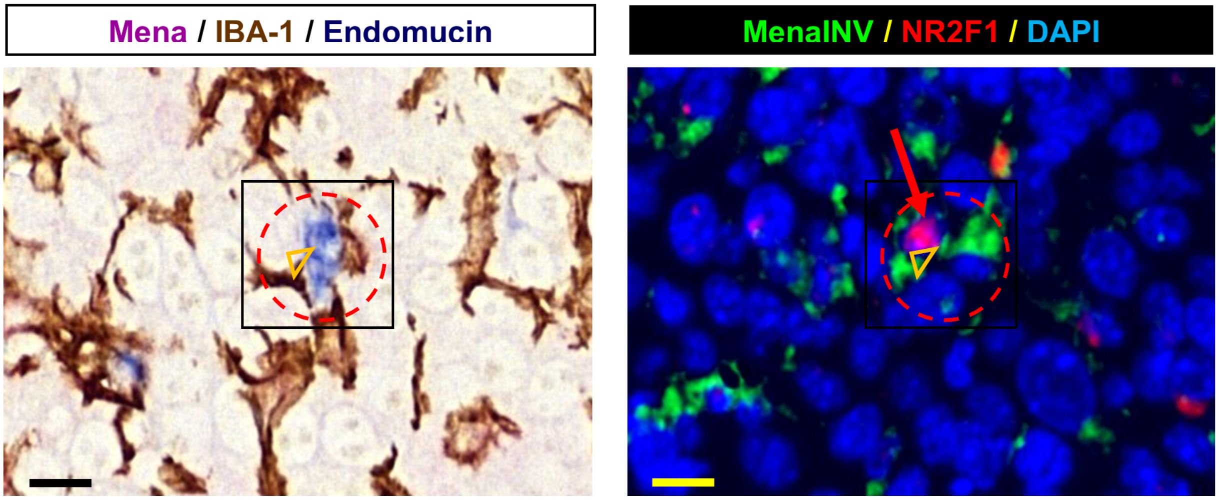

Supplementary Figure 6: Serial Sections of E0771-GFP Primary Tumor Tissue Stained for TMEM Doorways and GFP, NR2F1, and Mena^{INV}.

a: Left: Representative image of E0771-GFP primary tumor stained for TMEM doorways with a triple immunohistochemistry stain used for distance analysis shown in Figure 6b&c. Cells composing TMEM are positioned at vertices of yellow triangle: Pink=Mena expressing tumor cells; Brown=IBA-1 expressing macrophages; Blue=Endomucin expressing endothelial cells. Red dashed circle encompasses the perimeter of TMEM doorway. Scale bar=60 μm. **Right:** Tissue section sequential to Left immunofluorescently stained for GFP (not shown for clarity), Mena^{INV}, and, NR2F1: Green=Mena^{INV}; Red NR2F1; Blue=DAPI. Red dashed circle encompasses the perimeter of TMEM doorway. Red arrow points to an NR2F1 and Mena^{INV} double positive tumor cell. n=eight regions of interest (1-3 mm^2) in 4 mice.

Supp. Figure 7

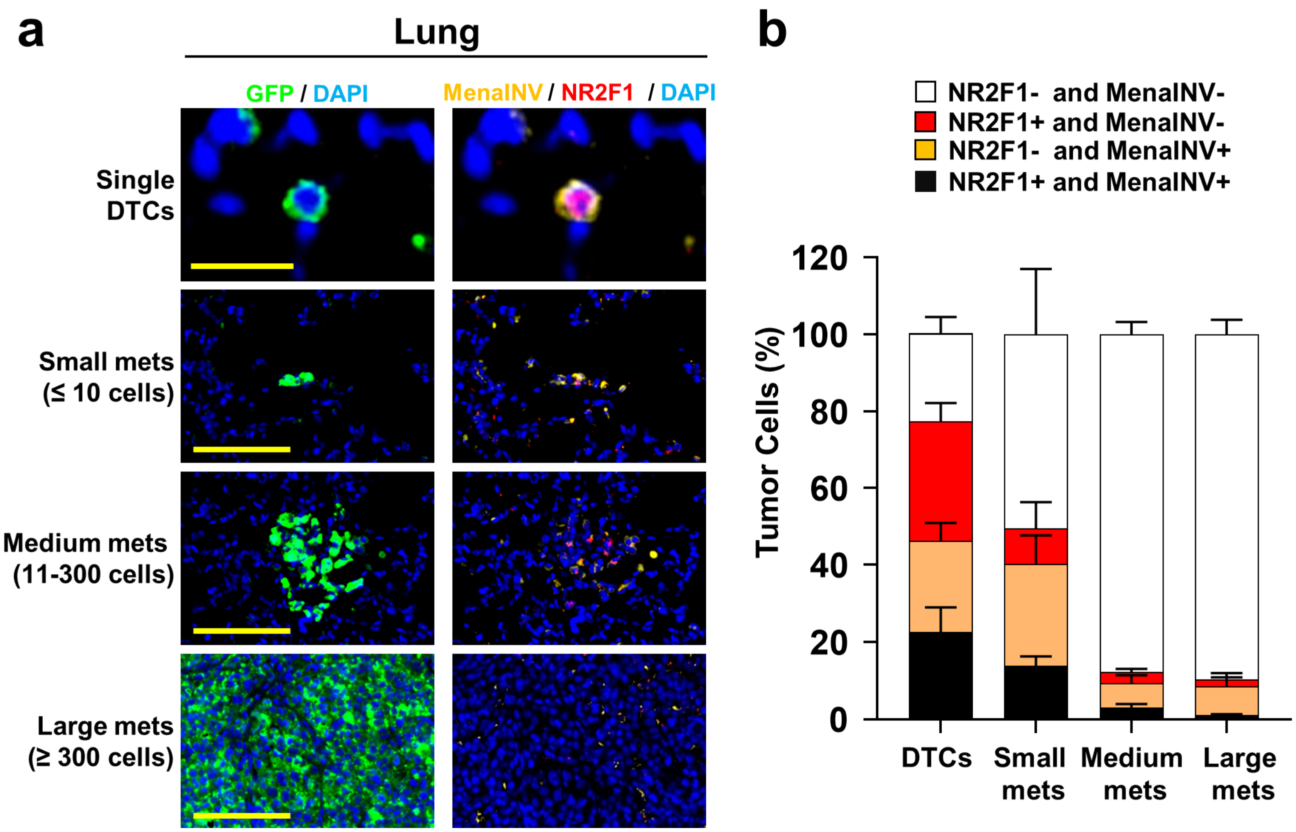

Supplementary Figure 7: Spontaneously Disseminated Tumor Cells Are Positive for NR2F1 and $\mathbf{Mena^{INV}}$.

a: Representative images of triple immunofluorescence staining for GFP, NR2F1, and $Mena^{INV}$ expression in disseminated tumor cells (DTCs) and at different stages of metastatic progression: small micro-metastases (≤10 cells), medium micrometastases (11-300 cells) and large micrometastases (≥300 cells) in an E0771-GFP SM model. Green=GFP; Orange=$Mena^{INV}$; Red=NR2F1; Blue=DAPI. Scale bar=20 μm for disseminated tumor cell and 100 μm for micrometastases.

b: Quantification of NR2F1 and $Mena^{INV}$ expression in single disseminated tumor cells (DTCs) and lung metastases at different stages of metastatic progression as shown in Supplemental Figure 7a. Disseminated tumor cells: n=452 cells in 5 animals. n=43 metastases analyzed in 5 mice. Bar=mean. Error bars=±SEM. Source data are provided as a Source Data file.

Supp. Figure 8

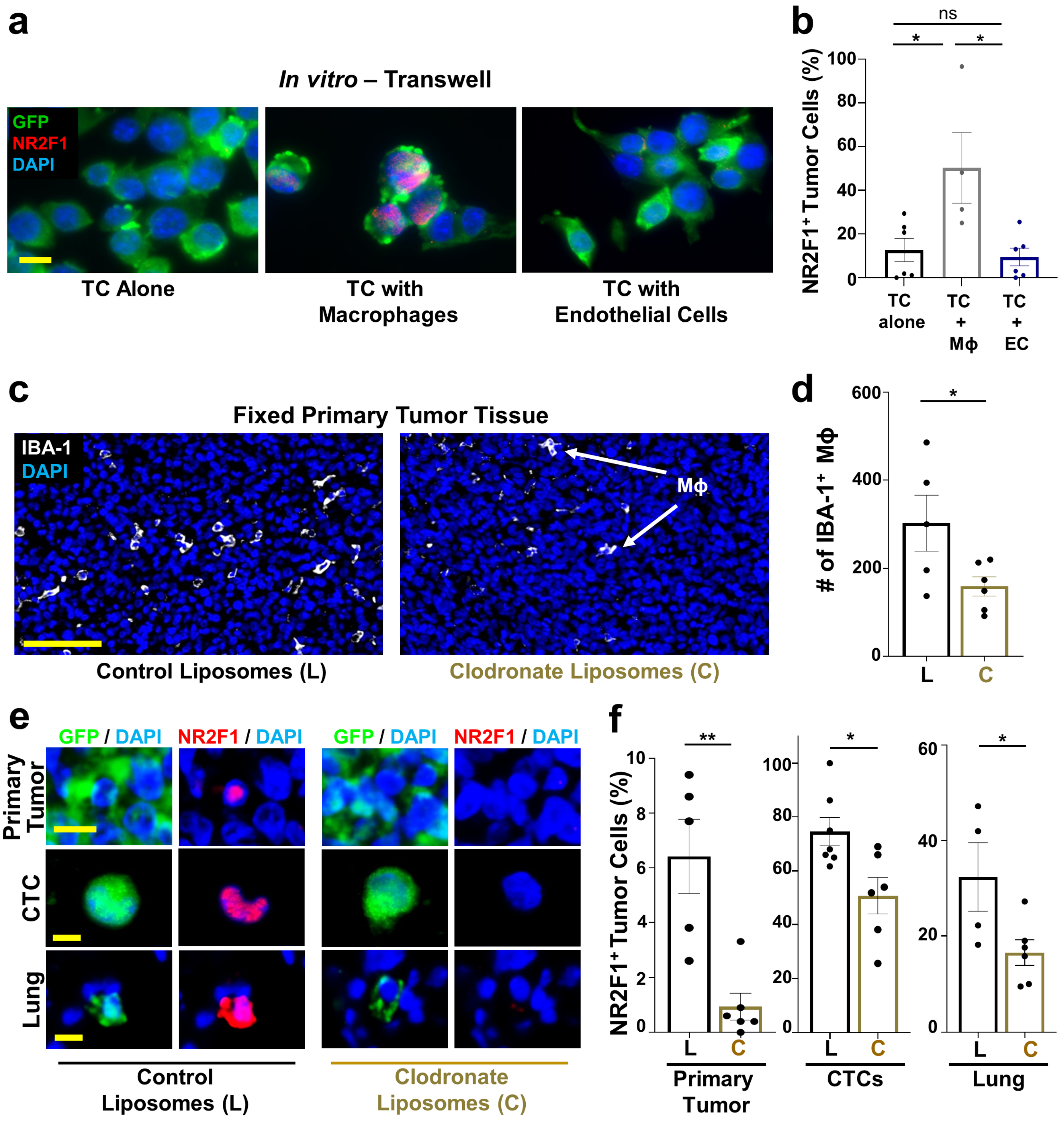

178

Supplementary Figure 8: Macrophages Regulate Dormancy in Disseminating Tumor cells

a: Representative immunofluorescence images of NR2F1 expression in E0771-GFP tumor cells cultured in a transwell system either alone (**Left**), together with macrophages (**Middle**), or together with endothelial cells (**Right**). Green=GFP; Red=NR2F1; Blue=DAPI. Scale bar=15 μm.

b: Percentage of NR2F1-positive tumor cells from each group in Supplemental Figure 8a. TC alone: n=734 cells in 6 independent experiments; TC + Mϕ; n=686 cells in 4 independent experiments. TC + EC; n=1,370 cells in 6 independent experiments. Bar=mean. Error bars=±SEM. For all comparisons a two-tailed one-way ANOVA test with Tukey's multiple comparisons adjustment was used. TC vs. TC + Mϕ: p=0.012, TC vs. TC + EC: p=0.95, TC + Mϕ vs. TC + EC: p=0.007. *=p<0.05.; ns=not significant. TC=Tumor Cell. Mϕ=macrophage. EC=Endothelial Cell.

c: Representative immunofluorescence images of 231-GFP primary tumor tissues treated for 7 days with either control liposomes or clodronate liposomes and stained for macrophages: IBA-1=White; DAPI=Blue. Scale bar for Tumor=100 μm. Mϕ=Macrophage.

d: Number of IBA-1 positive macrophages in 10 fields of view (960x568 $μm^2$) in each group from Supplemental Figure 8c. Control Liposomes: n=50 fields of view in 5 animals. Clodronate liposomes: n=60 fields of view in 6 animals. Two-tailed unpaired t-test (p=0.046).*=p<0.05. Bar=mean. Error bars=±SEM.

e: Representative immunofluorescence images of NR2F1 expression in primary tumors, circulating tumor cells (CTCs), and disseminated tumor cells (Lung) from a 231-GFP SM model treated with control liposomes (**Left**) or with clodronate liposomes (**Right**). Green=GFP, Red=NR2F1, Blue=DAPI. Scale bar for Primary Tumor=50 μm. Scale bar for CTCs and Lung=15 μm.

f: Percentage of NR2F1-positive tumor cells in each group from Supplemental Figure 8e. Control Liposomes - Primary Tumor: n=2,033 cells in 88 fields of view (65x65 $μm^2$) in 5 animals; CTCs: n=256 cells in 7 animals; Lung: n=98 cells in 4 animals. Clodronate Liposomes - Primary Tumor: n=2,829 cells in 109 fields of view (65x65 $μm^2$) in 6 animals; CTCs: n=337 cells in 6 animals; Lung: n=126 cells in 6 animals. Bar=mean. Error bars=±SEM. For Primary tumor columns (p=0.0087), a two-tailed Mann Whitney test was used. For CTCs (p=0.017) and Lung (p=0.043) columns, a two-tailed unpaired t-test was used.*=p<0.05. **=p<0.01. ns=not significant. Source data are provided as a Source Data file.

Supp. Figure 9

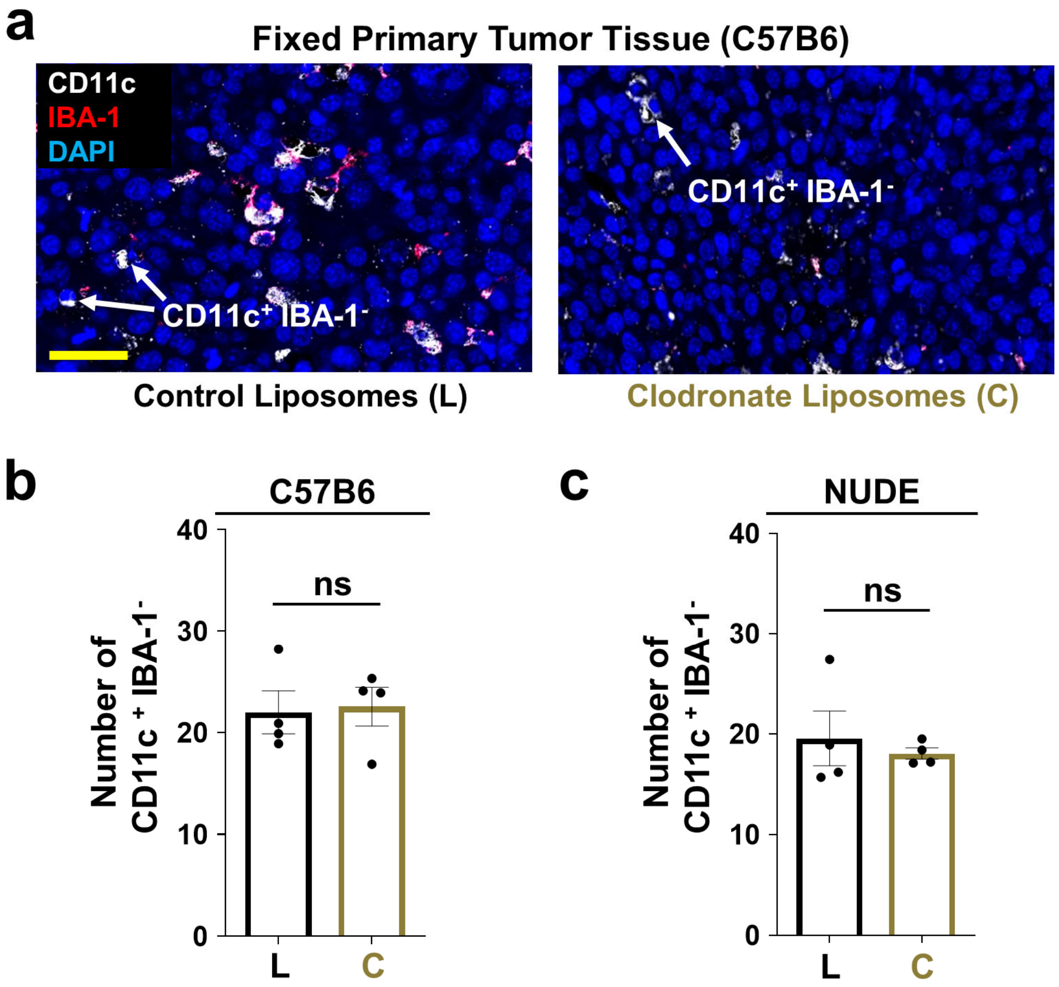

Supplementary Figure 9: Quantification of CD11c⁺ Cells in Primary Tumors.

a: Representative immunofluorescence images of E0771-GFP primary tumor tissues treated for 7 days with either control or clodronate liposomes and stained for dendritic cells ($CD11c^+$ $IBA\text{-}1^-$). White=CD11c; Red=IBA-1; Blue=DAPI. Mϕ=Macrophage. Scale bar =50 μm.

b: Percentage of CD11c positive and IBA-1 negative dendritic cells in 10 fields of view (486x236 μm^2) in each group from E0771-GFP primary tumor tissues. Control Liposomes: n=40 fields of view in 4 animals. Clodronate liposomes: n=40 fields of view in 4 animals. Bar=mean. Error bars=±SEM. Two-tailed unpaired t-test ($p=0.85$). ns=not significant.

c: Percentage of CD11c positive and IBA-1 negative dendritic cells in 10 fields of view (486x287 μm^2) in each group from MDA-MB-231-GFP primary tumor tissues. Control Liposomes: n=40 fields of view in 4 animals. Clodronate liposomes: n=40 fields of view in 4 animals. Error bars=±SEM. Two-tailed unpaired t-test ($p=0.61$). ns=not significant. Source data are provided as a Source Data file.

Supp. Figure 10

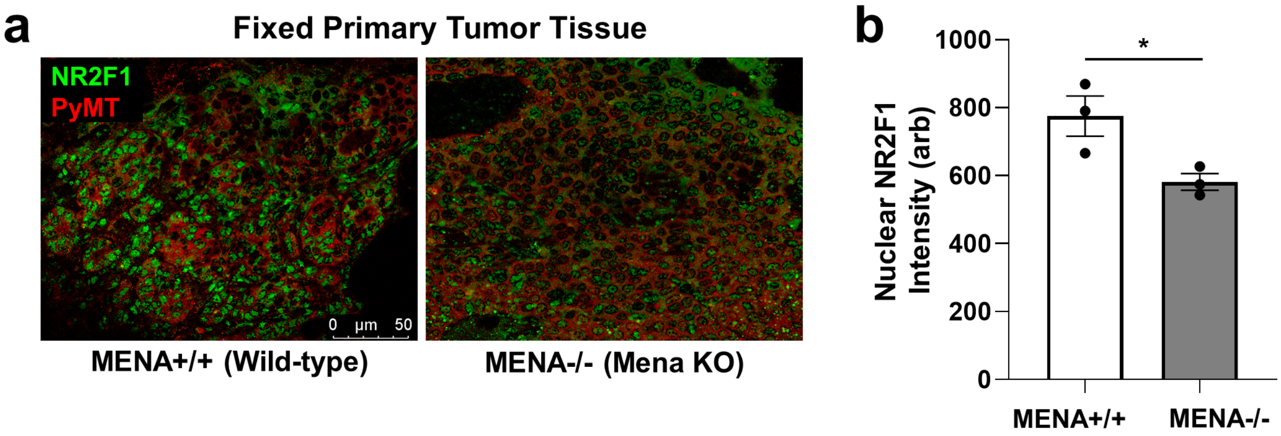

Supplementary Figure 10: Expression of NR2F1 in MMTV-PyMT Tumors Wild-Type and Knock-Out for Mena.

a: Representative immunofluorescence images of MMTV-PyMT primary tumors that are wild-type (**Left**), or knock-out for Mena (**Right**), and stained for NR2F1. Green=NR2F1; Red=PyMT. Scale bar=50 μm.

b: Quantification showing the intensity of nuclear NR2F1 expression in MMTV-PyMT primary tumors that are wild-type or knock-out for Mena. MMTV-PyMT primary tumor wild-type: n=2,756 cells in 47 fields of view (65x65 μm^2) in 3 animals. MMTV-PyMT primary tumor Mena knock-out: n=2,553 cells in 45 fields of view (65x65 μm^2) in 3 animals. Bar=mean. Error bars=±SEM. Two-tailed unpaired t-test (p=0.039).*=p<0.05. Source data are provided as a Source Data file.

Supp. Figure 11

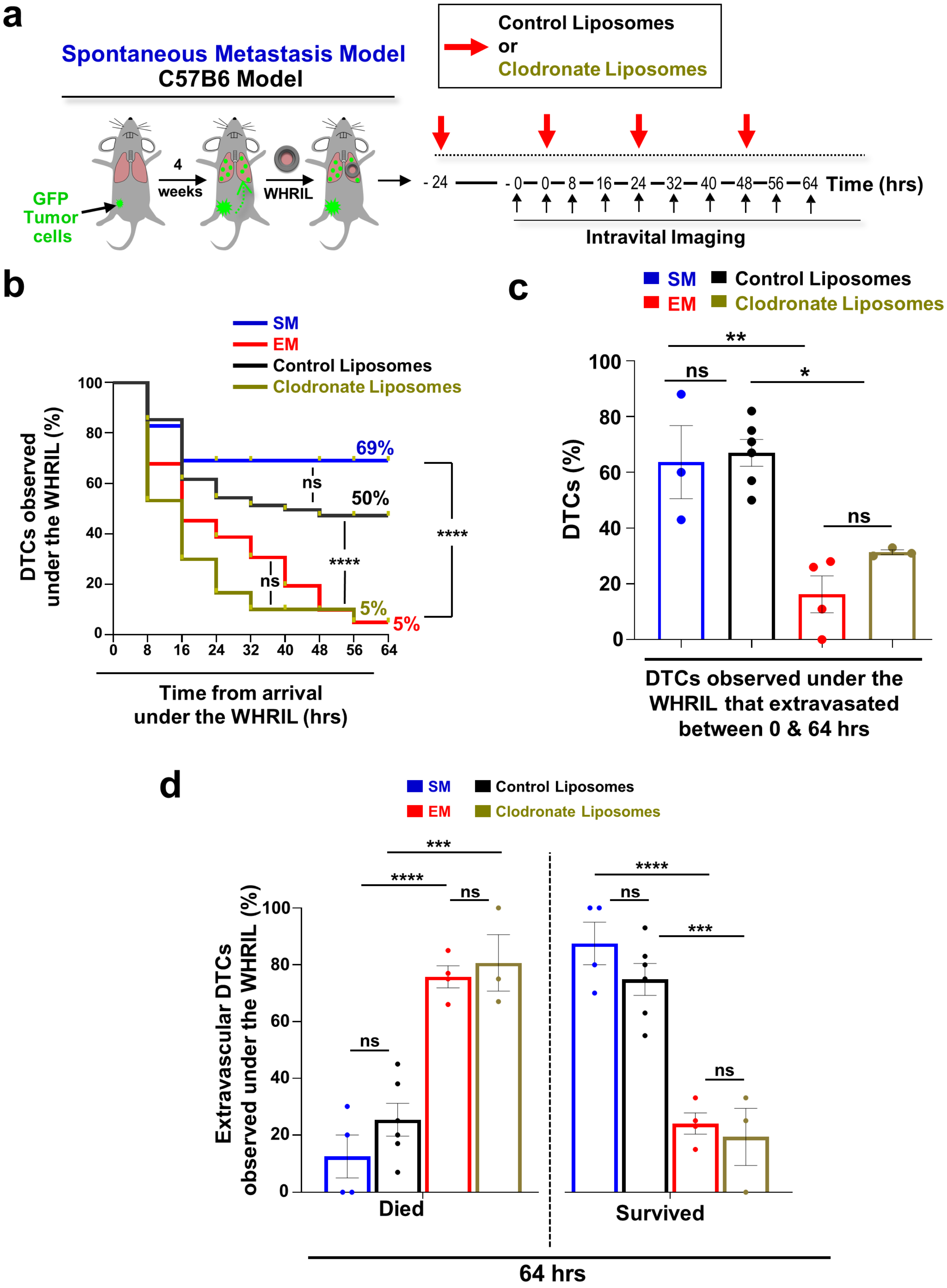

230

Supplementary Figure 11: Systemic Depletion of Macrophages Reduces Tumor Cell Retention, Extravasation, and Survival of DTCs in a SM Model.

a: Outline of the experimental design. GFP labeled tumor cells were injected into the mammary gland and tumors allowed to develop for ~4 weeks after which the WHRIL was surgically implanted and the mouse allowed to recover for 24 hrs. After that, mouse was treated with control or clodronate liposomes and imaged every 8 hrs for a period of 64 hrs.

b: Kaplan-Meier survival curves showing the percentage of E0771-GFP tumor cells observed under the WHRIL at each 8 hr time point over a period of 64 hrs in mice treated with control or clodronate liposomes, and in experimental metastasis (EM) and spontaneous metastasis (SM) models. EM and SM are same data from Figure 1c, for comparison. Control Liposomes: n=81 tumor cells analyzed in 6 mice. Clodronate Liposomes: n=32 tumor cells analyzed in 3 mice. EM: n=62 tumor cells analyzed in 3 mice. SM: n=28 tumor cells analyzed in 3 mice. Log-rank (Mantel-Cox) tests. EM vs. SM: p<0.0001, EM vs. Clodronate Liposomes: p=0.11, Clodronate Liposomes vs. Control Liposomes: p<0.0001, and Control Liposomes vs. SM: p=0.19. ****=p<0.0001. ns=not significant.

c: Percentage of E0771-GFP tumor cells observed under the WHRIL that extravasated between 0 and 64 hrs after arrival in mice treated with control or clodronate liposomes, and in experimental metastasis (EM) and spontaneous metastasis (SM) models. EM and SM are same data from Figure 1d, for comparison. Control Lipsomes: n=81 tumor cells analyzed in 6 mice. Clodronate Liposomes: n=32 tumor cells analyzed in 3 mice. EM: n=89 tumor cells analyzed in 4 mice. SM: n=29 tumor cells analyzed in 3 mice. Bar=mean. Error bars=±SEM. For all comparisons, a two-tailed one-way ANOVA test with Sidak's multiple comparisons adjustment was used. EM vs. SM: p=0.0036, EM vs. Clodronate Liposomes: p=0.57, Clodronate Liposomes vs. Control Liposomes: p=0.014, and Control Liposomes vs. SM: p=0.99. *=p<0.05. **=p<0.01. ns=not significant.

d: Percentage of extravascular E0771-GFP disseminated tumor cells that died or survived after extravasation in mice treated with control or clodronate liposomes, and in experimental metastasis (EM) and spontaneous metastasis (SM) models. EM and SM are same data from Figure 3b, for comparison. Control Liposomes: n=54 tumor cells in 6 mice. Clodronate Liposomes: n=10 tumor cells in 3 mice. EM: n=27 tumor cells analyzed in 4 mice. SM: n=31 tumor cells analyzed in 4 mice. Bar=mean. Error bars=±SEM. For all comparisons, a two-tailed one-way ANOVA test with Sidak's multiple comparisons adjustment was used. For Died: EM vs. SM: p<0.0001, EM vs. Clodronate Liposomes: p=0.98, Clodronate Liposomes vs. Control Liposomes: p=0.0003, and Control Liposomes vs. SM: p=0.52. For Survived: EM vs. SM: p<0.0001, EM vs. Clodronate Liposomes: p=0.99, Clodronate Liposomes vs. Control Liposomes: p=0.0003, and Control Liposomes vs. SM: p=0.52. ***=p<0.001. ****=p<0.0001. ns=not significant. Source data are provided as a Source Data file.

Supp. Figure 12

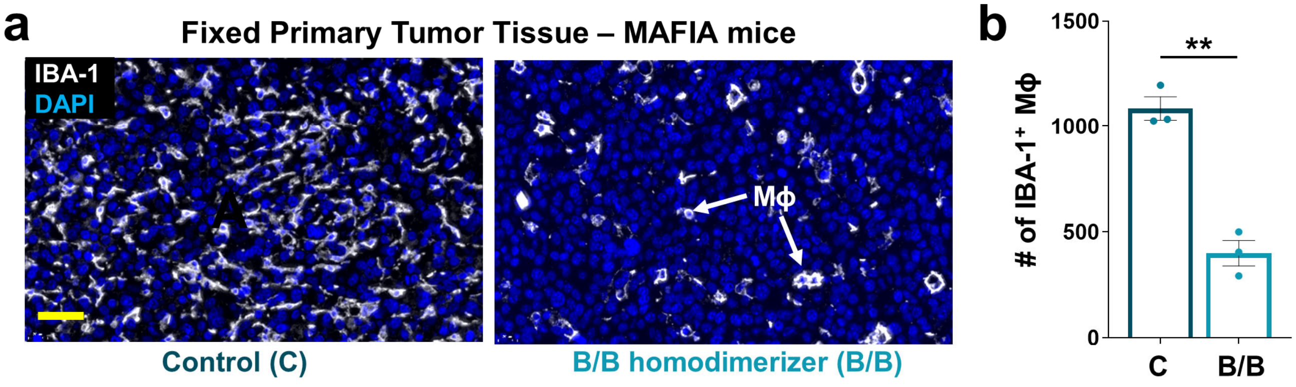

Supplementary Figure 12: Quantification of IBA-1$^+$ macrophages in primary breast tumors of MaFIA mice.

a: Representative immunofluorescence images of E0771-GFP primary tumor tissues treated for 7 days with either control (**Left**) or B/B homodimerizer (**Right**), and stained for macrophages: White=IBA-1; Blue=DAPI. Scale bar =50 μm. Mϕ=Macrophage.

b: Percentage of IBA-1 positive macrophages in 10 fields of view (963x570 μm^2) in each group of treated E0771 primary tumor tissues. Control: n=30 fields of view in 3 animals. B/B homodimerizer: n=30 fields of view in 3 animals. Bar=mean. Error bars=±SEM. Two-tailed unpaired t-test ($p=0.0011$). **=$p<0.01$. Source data are provided as a Source Data file.

Supp. Figure 13

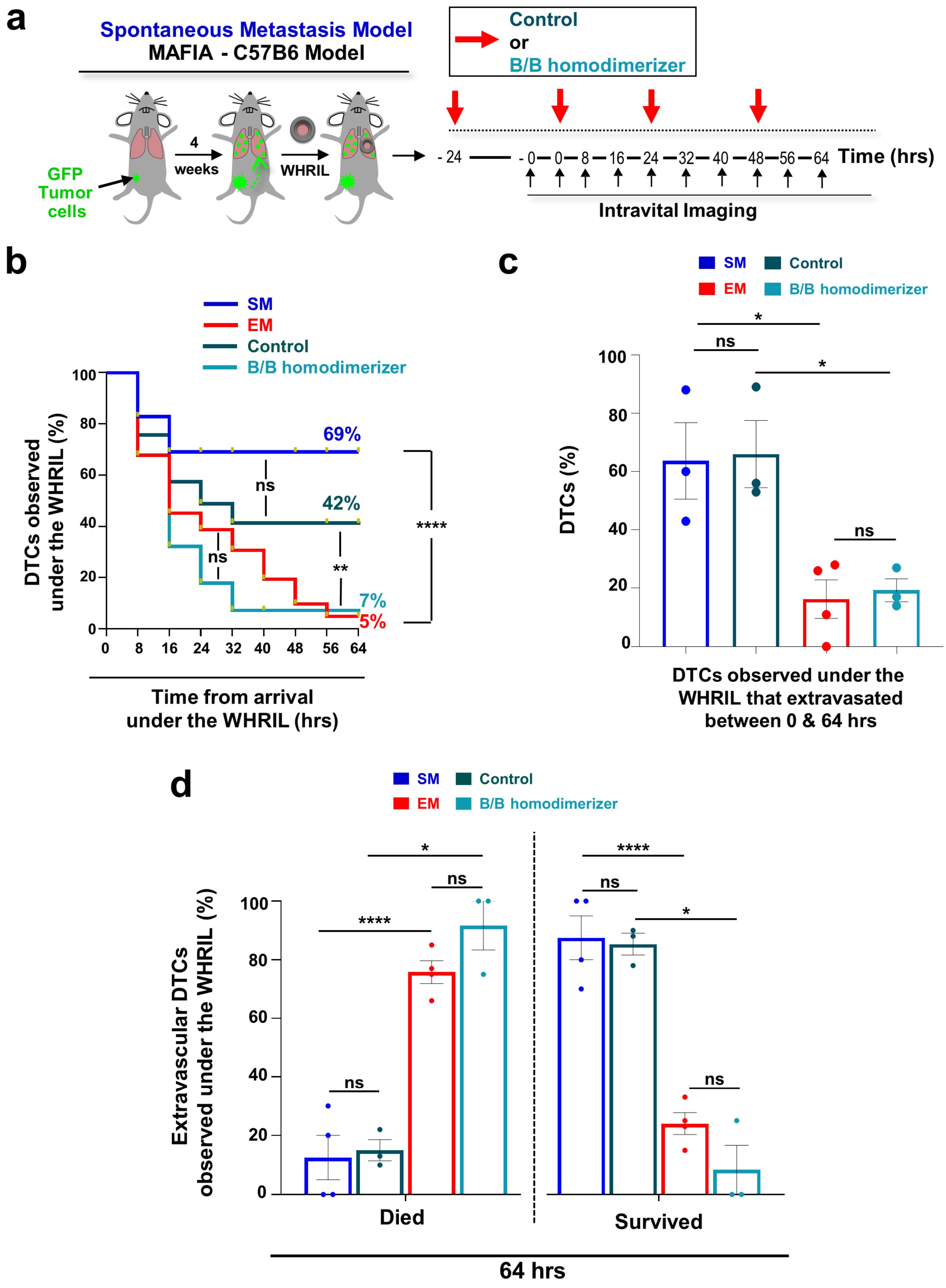

275

Supplementary Figure 13: Macrophage Depletion with B/B Homodimerizer in MaFIA Mice Reduces Tumor Cell Retention, Extravasation, and Survival of DTCs in the Lung.

a: Outline of the experimental design. GFP labeled tumor cells were injected into the mammary gland and tumors allowed to develop for ~4 weeks after which the WHRIL was surgically implanted and the mouse allowed to recover for 24 hrs. After that, mouse was treated with control or B/B homodimerizer and imaged every 8 hrs for a period of 64 hrs.

b: Kaplan-Meier survival curves showing the percentage of E0771-GFP tumor cells observed under the WHRIL at each 8 hr time point over a period of 64 hrs in mice treated with control or B/B homodimerizer, and in experimental metastasis (EM) and spontaneous metastasis (SM) models. EM and SM are same data from Figure 1c, for comparison. Control: n=44 tumor cells analyzed in 3 mice. B/B: n=28 tumor cells analyzed in 3 mice. EM: n=62 tumor cells analyzed in 3 mice. SM: n=28 tumor cells analyzed in 3 mice. Log-rank (Mantel-Cox) tests. EM vs. SM: $p<0.0001$, EM vs. B/B: $p=0.15$, Control vs. B/B: $p=0.0022$, and Control vs. SM: $p=0.092$. ****=$p<0.0001$. **=$p<0.01$. ns=not significant.

c: Percentage of E0771-GFP tumor cells observed under the WHRIL that extravasated between 0 and 64 hrs after arrival in mice treated with control or B/B homodimerizer, and in experimental metastasis (EM) and spontaneous metastasis (SM) models. EM and SM are same data from Figure 1d, for comparison. Control: n=44 tumor cells analyzed in 3 mice. B/B: n=28 tumor cells analyzed in 3 mice. EM: n=89 tumor cells analyzed in 4 mice. SM: n=29 tumor cells analyzed in 3 mice. Bar=mean. Error bars=±SEM. For all comparisons, a two-tailed one-way ANOVA test with Sidak's multiple comparisons adjustment was used. EM vs. SM: $p=0.018$, EM vs. B/B: $p=1.0$, Control vs. B/B: $p=0.028$, and Control vs. SM: $p=1.0$. *=$p<0.05$. ns=not significant.

d: Percentage of extravascular E0771-GFP disseminated tumor cells that died or survived after extravasation in mice treated with control or B/B homodimerizer, and in experimental metastasis (EM) and spontaneous metastasis (SM) models. EM and SM are same data from Figure 3b, for comparison. Control: n=27 tumor cells in 3 mice. B/B homodimerizer: n=6 tumor cells in 3 mice. EM: n=27 tumor cells analyzed in 4 mice. SM: n=31 tumor cells analyzed in 4 mice. Bar=mean. Error bars=±SEM. For Control *vs.* SM and for SM *vs.* EM, a two-tailed one-way ANOVA with Sidak’s multiple comparisons adjustment was used. For B/B *vs.* EM and for Control *vs.* B/B, a Kruskal-Wallis test with Dunn’s multiple comparisons adjustment was used. For Died: EM vs. SM: $p<0.0001$, EM vs. B/B: $p=1.0$, Control vs. B/B: $p=0.043$, and Control vs. SM: $p=0.88$. For Survived: EM vs. SM: $p<0.0001$, EM vs. B/B: $p=1.0$, Control vs. B/B: $p=0.043$, and Control vs. SM: $p=0.90$. *=$p<0.05$. ****=$p<0.0001$. ns=not significant. Source data are provided as a Source Data file.

Supp. Figure 14

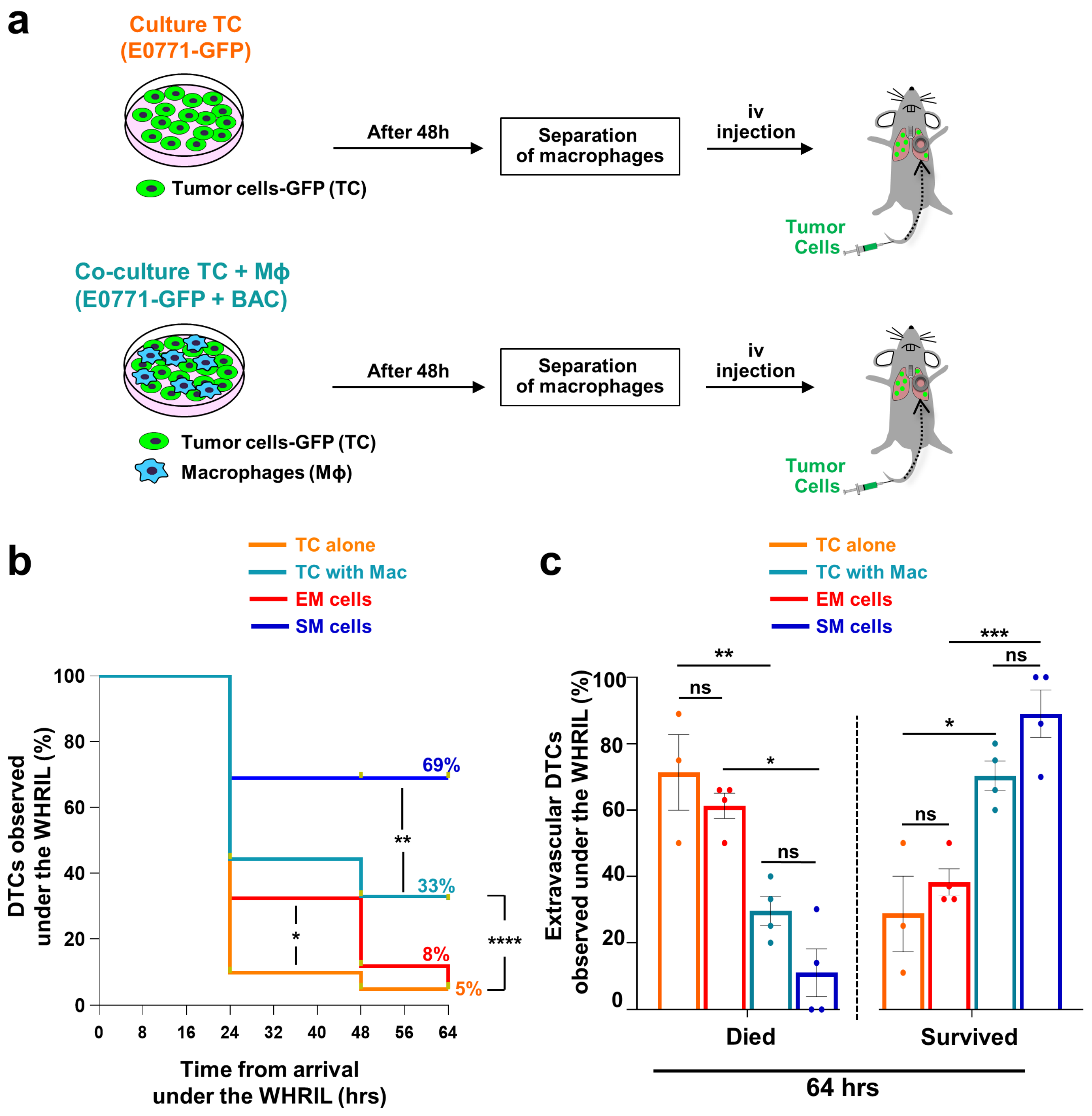

Supplementary Figure 14: Effect of Macrophages in Educating Tumor Cells to Be Retained and Survive in the Lung.

a: Outline of the experimental design. Tumor cells (TC) were cultured alone or with macrophages for 48 hrs. After that, macrophages were purified via the CD11b MicroBeads (as described in Material and Methods) separation column, and then injected into the tail vein of mice bearing a lung imaging window. Then, the fate of each TCs was tracked using intravital imaging every 24 hrs for a period of 64 hr.

b: Kaplan-Meier survival curves showing the percentage of E0771-GFP tumor cells cultured alone (TC alone), co-cultured with macrophages (TC with Mac), intravenously injected in mice (EM cells), or spontaneously disseminated from a primary tumor (SM cells), observed under the WHRIL every 24 hr over a period of 64 hrs. EM and SM data are from Figure 1c, but binned at a 24 hr interval, for comparison. Tumor cells cultured alone: n=62 cells in 3 mice. Tumor cells co-cultured with macrophages: n=87 cells in 4 mice. EM: n=60 cells in 3 mice. SM: n=28 cells in 3 mice. Log-rank (Mantel-Cox) tests. SM vs. TC with Mac: p=0.0030, EM vs. TC alone: p=0.020, and TC alone vs. TC with Mac: p<0.0001. *=p<0.05. **=p<0.01. ****=p<0.0001.

c: Percentage of extravascular E0771-GFP disseminated tumor cells that died or survived after extravasation. Tumor cells cultured alone: n=27 tumor cells in 4 mice. Tumor cells co-cultured with macrophages: n=38 cells in 4 mice. EM: n=19 cells in 4 mice. SM: n=31 tumor cells in 4 mice. EM and SM data are from Figure 3b, but binned at a 24 hr interval, for comparison. For EM vs. TC alone and for EM vs. SM died columns, a Kruskal-Wallis test with Dunn's multiple comparisons adjustment was used. For Died: EM vs. TC alone: p=1.00, TC alone vs. TC with Mac: p=0.010, EM vs. SM: p=0.016, and SM vs. TC with Mac: p=0.20. For Survived: EM vs. TC alone: p=0.82, TC alone vs. TC with Mac: p=0.019, EM vs. SM: p=0.0003, and SM vs. TC with Mac: p=0.23. For all other statistical comparisons, two-sided one-way ANOVA test with Sidak's multiple comparison adjustment were used. *=p<0.05. **=p<0.01. ***=p<0.001. ns=not significant. Source data are provided as a Source Data file.

Supp. Figure 15

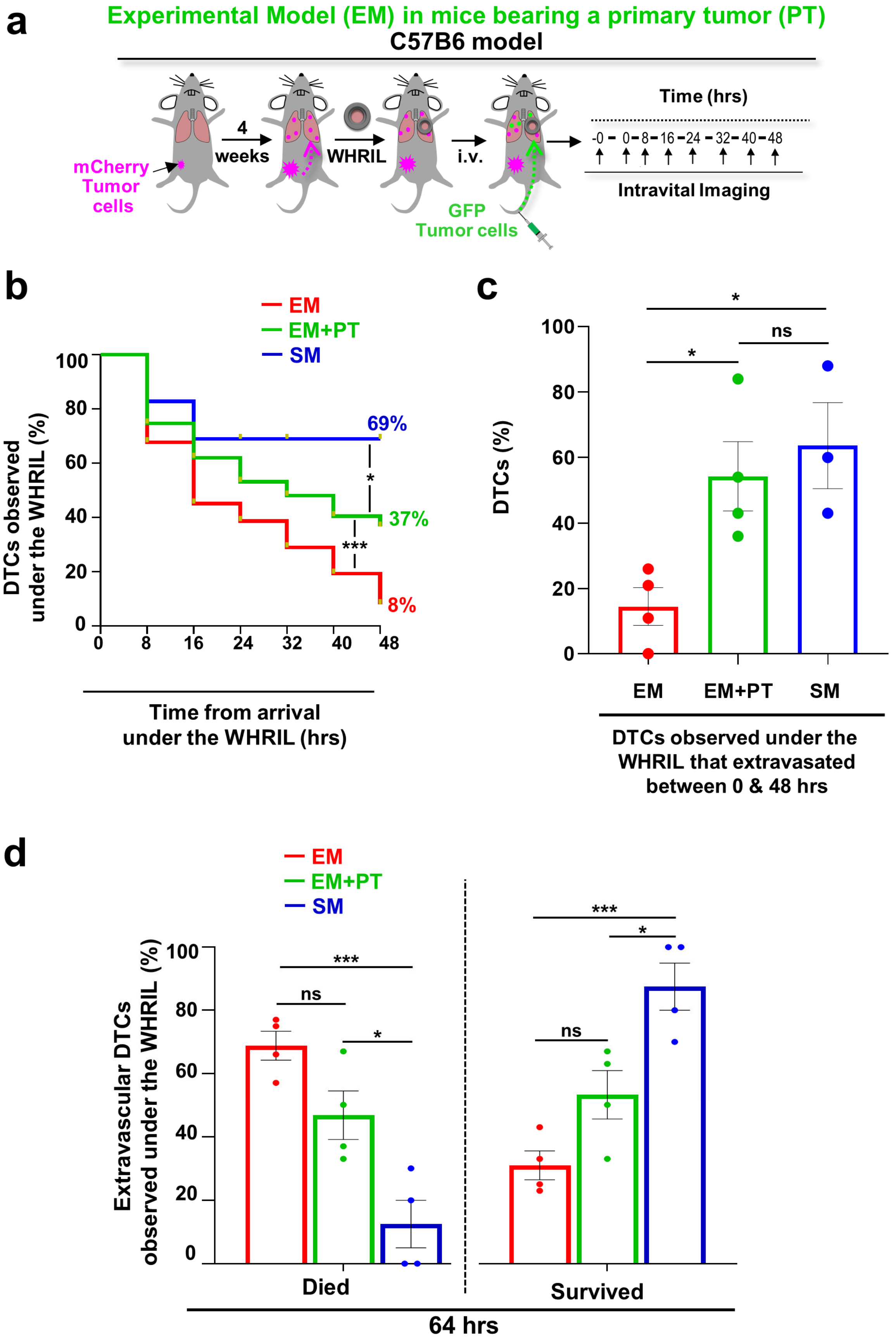

Supplementary Figure 15: Influence of the Primary Tumor on the Initial Steps of Lung Metastasis

a: Outline of the experimental design. mCherry labeled tumor cells (E0771-mCherry) were injected into the mammary gland and tumors allowed to develop for ~4 weeks after which the WHRIL was surgically implanted and the mouse allowed to recover for 24 hrs. After that, GFP-labelled tumor cells (E0771-GFP) were injected into the tail vein of the mouse, and their fate followed every 8 hrs for 48 hrs.

b: Kaplan-Meier survival curves showing the percentage of E0771-GFP tumor cells observed under the WHRIL at each 8 hr time point over a period of 48 hrs. EM and SM data are from Figure 1c, but truncated at 48hrs for comparison. EM: $n=62$ tumor cells analyzed in 3 mice. EM with primary tumor (EM+PT): $n=79$ tumor cells analyzed in 4 mice. SM: $n=29$ tumor cells analyzed in 3 mice. Log-rank (Mantel-Cox) tests. EM vs. EM + PT: $p=0.0006$ and EM + PT vs. SM: $p=0.025$. *= $p<0.005$. ***= $p<0.001$.

c: Percentage of E0771-GFP tumor cells in EM, EM+PT and SM groups, observed under the WHRIL that extravasated between 0 and 48 hrs after arrival. EM and SM data are from Figure 1d, but truncated at 48hrs, for comparison). EM: $n=89$ tumor cells analyzed in 4 mice. EM with PT: $n=79$ tumor cells in 4 mice. SM: $n=29$ tumor cells analyzed in 3 mice. Bar=mean. Error bars=±SEM. Two-tailed one-way ANOVA test with Tukey's multiple comparisons adjustment. EM vs. EM + PT: $p=0.040$, EM vs. SM: $p<0.021$, and EM + PT vs. SM: $p=0.79$. *= $p<0.01$. ns=not significant.

d: Percentage of extravascular E0771-GFP disseminated tumor cells that died or survived after extravasation in EM, EM+PT and SM models. EM and SM data are from Figure 3b, but truncated at 48 hrs, for comparison. EM: $n=26$ tumor cells analyzed in 4 mice. EM with PT: $n=44$ tumor cells in 4 mice. SM: $n=31$ tumor cells analyzed in 4 mice. Bar=mean. Error bars=±SEM. For all comparisons, two tailed one-way ANOVA tests with Sidak's multiple comparisons adjustments were used. For Died: EM vs. EM + PT: $p=0.13$, EM vs. SM: $p<0.0007$, and EM + PT vs. SM: $p=0.017$. For Survived: EM vs. EM + PT: $p=0.13$, EM vs. SM: $p<0.0007$, and EM + PT vs. SM: $p=0.17$. *= $p<0.05$. ***= $p<0.001$. ns=not significant.
Source data are provided as a Source Data file.

Supp. Figure 16

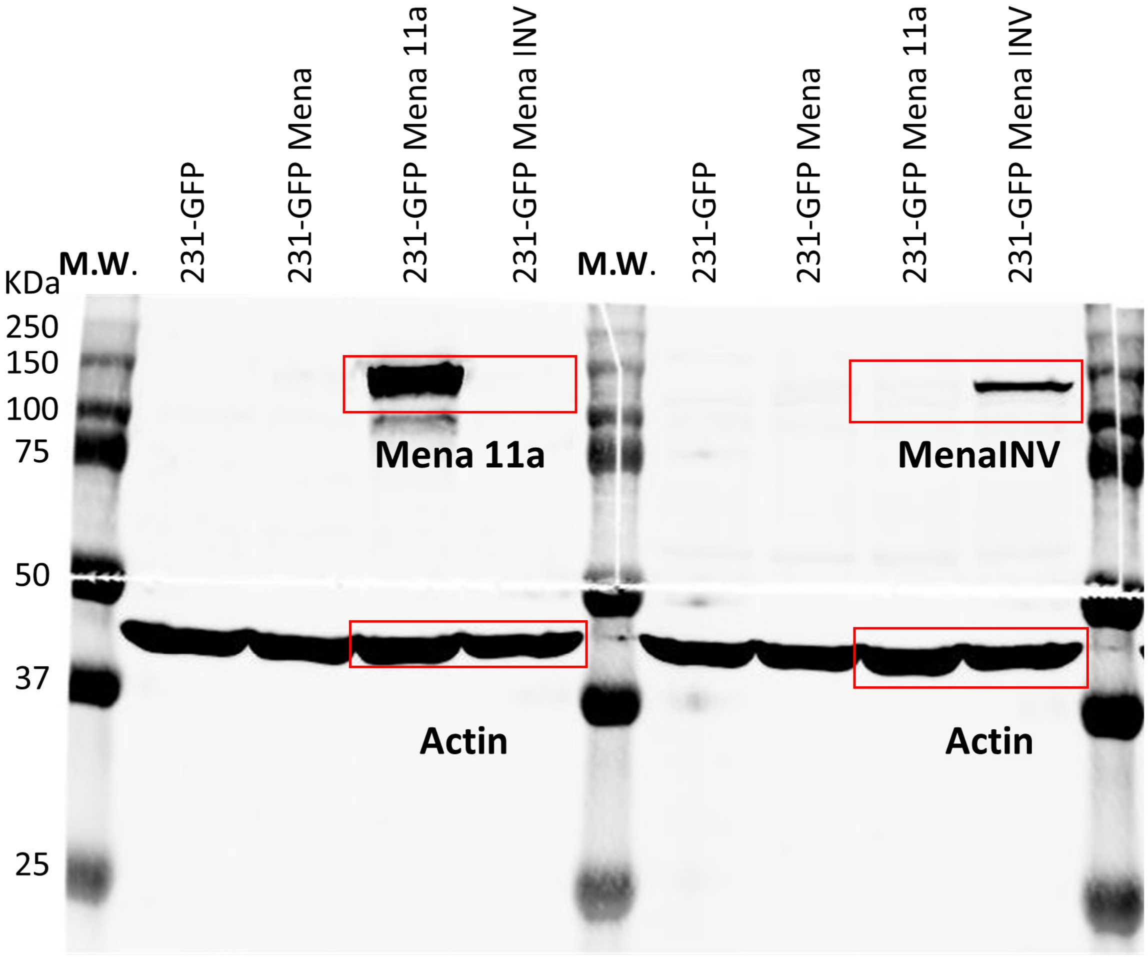

Supplementary Figure 16: Uncropped blot for Figure 2c. Red boxes indicate the bands which were cropped for Figure 2c. Source data are provided as a Source Data file.

Supp. Figure 17

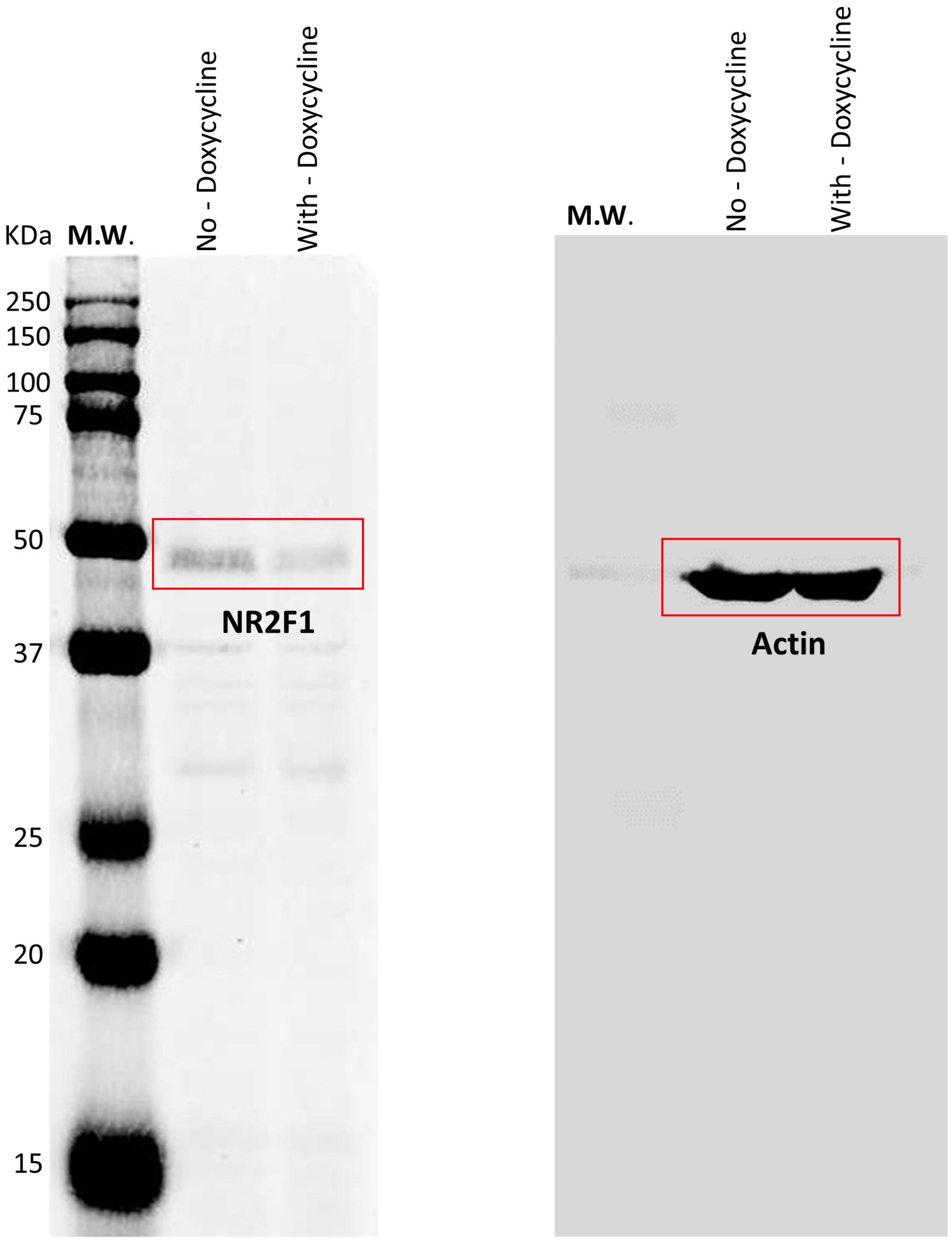

361

Supplementary Figure 17: Uncropped blot for Supplemental Figure 5b. Red boxes indicate the bands which were cropped for Supplemental Figure 5b. Source data are provided as a Source Data file.
