# Supplementary material for: Primary tumor associated macrophages activate programs of invasion and dormancy in disseminating tumor cells
Source: Nat Commun. 2022 Feb 2;13:626. doi: 10.1038/s41467-022-28076-3 (PMC8811052; doi:10.1038/s41467-022-28076-3)
Supplement: Supplementary file 5 — Description of Additional Supplementary Files [file 41467_2022_28076_MOESM5_ESM.pdf]

Supplementary Movie Legends

Title: Supplementary Movie 1:
Description: Time Lapse movie of a tumor cell. Left: Red (blood) channel was averaged to improve definition of the vascular boundaries. Right: Raw imaging data. Red = 155kD TMRdextran labeled vasculature. Green = GFP tumor cell.

Title: Supplementary Movie 2:
Description: Time Lapse movie corresponding to Supplemental Figure 1a showing the movement of an intravascular disseminated tumor cell. Left: Red (blood) channel was averaged to improve definition of the vascular boundaries. Right: Raw imaging data. Red = 155kD TMR-dextran labeled vasculature. Green = GFP tumor cell. Cyan = Macrophages.

Title: Supplementary Movie 3:
Description: Time Lapse movie corresponding to Supplemental Figure 1b showing the movement of an extravascular disseminated tumor cell. Left: Red (blood) channel was averaged to improve definition of the vascular boundaries. Right: Raw imaging data. Red = 155kD TMR-dextran labeled vasculature. Green = GFP tumor cell.
