# Supplementary material for: Primary tumor associated macrophages activate programs of invasion and dormancy in disseminating tumor cells
Source: Nat Commun. 2022 Feb 2;13:626. doi: 10.1038/s41467-022-28076-3 (PMC8811052; doi:10.1038/s41467-022-28076-3)
Supplement: Supplementary file 6 — Reporting Summary [file 41467_2022_28076_MOESM6_ESM.pdf]

nature portfolio

Corresponding author(s): David Entenberg, John Condeelis, and Julio Aguirre-Ghiso

Last updated by author(s): 12/09/2021

Reporting Summary

Nature Portfolio wishes to improve the reproducibility of the work that we publish. This form provides structure for consistency and transparency in reporting. For further information on Nature Portfolio policies, see our Editorial Policies and the Editorial Policy Checklist.

Statistics

For all statistical analyses, confirm that the following items are present in the figure legend, table legend, main text, or Methods section.

n/a	Confirmed	
☐	☒	The exact sample size (*n*) for each experimental group/condition, given as a discrete number and unit of measurement
☐	☒	A statement on whether measurements were taken from distinct samples or whether the same sample was measured repeatedly
☐	☒	The statistical test(s) used AND whether they are one- or two-sided<br>*Only common tests should be described solely by name; describe more complex techniques in the Methods section.*
☒	☐	A description of all covariates tested
☐	☒	A description of any assumptions or corrections, such as tests of normality and adjustment for multiple comparisons
☐	☒	A full description of the statistical parameters including central tendency (e.g. means) or other basic estimates (e.g. regression coefficient) AND variation (e.g. standard deviation) or associated estimates of uncertainty (e.g. confidence intervals)
☐	☒	For null hypothesis testing, the test statistic (e.g. *F*, *t*, *r*) with confidence intervals, effect sizes, degrees of freedom and *P* value noted<br>*Give P values as exact values whenever suitable.*
☒	☐	For Bayesian analysis, information on the choice of priors and Markov chain Monte Carlo settings
☒	☐	For hierarchical and complex designs, identification of the appropriate level for tests and full reporting of outcomes
☒	☐	Estimates of effect sizes (e.g. Cohen's *d*, Pearson's *r*), indicating how they were calculated

Our web collection on statistics for biologists contains articles on many of the points above.

Software and code

Policy information about availability of computer code

Data collection	Custom-built multiphoton microscope acquisition software, PMID: 21959234<br>Pannoramic 250 Flash II digital whole slide scanner<br>Epi-fluorescence microscope (GE, DeltaVision) with a 100x objective, and CoolSNAP HQ2 CCD camera<br>LI-COR Odyssey Western blot
Data analysis	GraphPad Prism v9<br>HyperStackReg plugin v5.6 (https://github.com/ved-sharma/HyperStackReg)<br>SPSS v24<br>ImageJ/FIJI v 1.53c<br>For NR2F1-TMEM distance analysis, a previously published custom ImageJ/FIJI macro was used (Sharma et al. Nature Comm., in press)<br>Excel 2016

For manuscripts utilizing custom algorithms or software that are central to the research but not yet described in published literature, software must be made available to editors and reviewers. We strongly encourage code deposition in a community repository (e.g. GitHub). See the Nature Portfolio guidelines for submitting code & software for further information.

Data

Policy information about availability of data

All manuscripts must include a data availability statement. This statement should provide the following information, where applicable:

- Accession codes, unique identifiers, or web links for publicly available datasets
- A description of any restrictions on data availability
- For clinical datasets or third party data, please ensure that the statement adheres to our policy

The authors declare that all data supporting the findings of this study are available within the paper. Source data are provided with this paper.

Field-specific reporting

Please select the one below that is the best fit for your research. If you are not sure, read the appropriate sections before making your selection.

[x] Life sciences [ ] Behavioural & social sciences [ ] Ecological, evolutionary & environmental sciences

For a reference copy of the document with all sections, see nature.com/documents/nr-reporting-summary-flat.pdf

Life sciences study design

All studies must disclose on these points even when the disclosure is negative.

Sample size	All in vivo and in vitro experiments were independently repeated and included biologically independent samples, as indicated in the figure legends. Sample size was chosen empirically. Unless otherwise specified in the figure legends, statistical significance between groups was determined using unpaired two-tailed Student's t-tests or ANOVA for normally distributed data (checked with the Shapiro-Wilk test) or with Mann-Whitney or Kruskal-Wallis tests for non-normally distributed data. Differences were considered significant for p<0.05.
Data exclusions	No data points were excluded, except:<br>1) For in vivo experiments, mice that died during intravital imaging sessions or the fate of disseminated tumor cells which could not followed for the entire experimental design, were removed from the study or marked as censored.<br>2) For quantification of the intravital imaging data, disseminated tumor cells in the lungs were excluded if their localization (intravascular vs. extravascular) could not be accurately determined.<br>3) For quantification of IF staining, fields of view that were necrotic or of quality too poor for proper analysis and image quantification were excluded.
Replication	Each graph represents individual sample or mice used.<br>The number of replicates is indicated in each figure legend.
Randomization	Female mice of the same age were used for experiments. For all in vivo experiments (which included experiments with clodronate or B/B homodimer), mice were randomly allocated into two experimental groups: control and treatment. No, other treatments were performed
Blinding	The investigators were not blinded to mouse treatments. Mice were assigned into the therapy groups based on the size of the tumor and treatment was then performed. Treatments were know to the investigators, making complete3 blinding difficult. However, all in vivo and experiments from i control and treatment groups were analyzed in concert, preventing bias.

Reporting for specific materials, systems and methods

We require information from authors about some types of materials, experimental systems and methods used in many studies. Here, indicate whether each material, system or method listed is relevant to your study. If you are not sure if a list item applies to your research, read the appropriate section before selecting a response.

Materials & experimental systems

n/a	Involved in the study	
[ ]	[x]	Antibodies
[ ]	[x]	Eukaryotic cell lines
[x]	[ ]	Palaeontology and archaeology
[ ]	[x]	Animals and other organisms
[x]	[ ]	Human research participants
[x]	[ ]	Clinical data
[x]	[ ]	Dual use research of concern

Methods

n/a	Involved in the study	
[x]	[ ]	ChIP-seq
[x]	[ ]	Flow cytometry
[x]	[ ]	MRI-based neuroimaging

Antibodies

Antibodies used	For Immunofluorescence staining:<br>GFP (Novus, cat #NB100-1614, concentration 100 μg/mL)<br>NR2F1 (Abcam, cat #ab181137, concentration 5 μg/mL)<br>SOX9 (Millipore, cat #AB5535, concentration 1 μg/mL)<br>SOX9 (RD, cat #AF3075, concentration 1 μg/mL)<br>MenaINV (generated in the Condeelis Laboratory, concentration 0.25 μg/mL),<br>Endomucin (Santa Cruz, cat #sc-65495, concentration 2 μg/mL).<br>IBA-1 (Wako, cat #019-19741, concentration 0.05 μg/mL)<br>CD11c (Invitrogen, cat #PA5-90208, concentration 1.5 μg/mL)<br>PyMT (Novus, cat #NB100-2749, concentration 0.01 μg/mL)<br>For secondary Abs, (Invitrogen, concentration 1 μg/mL)<br>Alexa Fluor 546 for NR2F1 or for SOX9 (Invitrogen, cat #A11034, concentration 1 μg/mL).<br><br>For TMEM Immunohistochemical staining<br>pan-Mena antibody (BD, cat. #610693, concertation 5 μg/mL)<br>IBA-1 antibody (Wako, cat. #019-19741, concentration 0.167 μg/mL)<br>endomucin (Santa Cruz, cat #sc-65495, concentration 0.67 μg/mL)<br><br>For western blotting:<br>Chicken anti-MenaINV (generated in the Condeelis Laboratory, concentration 0.25 μg/mL)<br>Rabbit anti-Mena11a (generated in the Condeelis Laboratory , concentration 1 mg/mL)<br>Rabbit anti-NR2F1 (Abcam, cat #ab181137, concentration 0.5 μg/mL) and mouse anti-actin (Sigma, cat #A5441, 1 μg/mL)<br><br>Secondary Antibodies:<br>Goat anti-Chicken, Secondary Antibody, Alexa Fluor 488 (ThermoFisher, cat#A-11039, concentration 1 μg/mL)<br>Goat anti-Chicken, Secondary Antibody, Alexa Fluor 488 (ThermoFisher, cat#A-11039, concentration 1 μg/mL)<br>Donkey anti-Rabbit, Secondary Antibody, Alexa Fluor 555 (ThermoFisher, cat#A-31572, concentration 1 μg/mL)<br>Donkey anti-chicken, Secondary Antibody, Alexa Fluor 568 (Jackson, cat#703-025-155, concentration 0.5 μg/mL)<br>Goat anti-Rat, Secondary Antibody, Alexa Fluor 647 (ThermoFisher, cat#A-21247, concentration 1 μg/mL)<br>Goat anti-Rat, Secondary Antibody, Alexa Fluor 568 (ThermoFisher, cat#A-11077, concentration 1 μg/mL)
Validation	Antibodies were validated in our previous studies or were chosen due to extensive use in prior publications and further validated in our laboratory according to manufacturer's instructions.

Eukaryotic cell lines

Policy information about cell lines

Cell line source(s)	E0771-GFP cells were obtained from Dr. Wakefield's lab at the NIH who obtained them directly from Dr. Fengzhi Li in Dr. Enrico Mihich's lab at Roswell Park Cancer Institute, Buffalo, NY<br><br>MDA-MB-231 cells were purchased from ATCC. Sublines stably expressing GFP were generated as previously described (PMID: 10892743)<br>MDA-MB-231-GFP cells over-expressing MenaINV or Mena11a were generated as previously described (PMID: 21670198)<br><br>shRNAmir-NR2F1 cells were generated by TURBO-RFP-shNR2F1-mir-encoding lentivirus infection of MDA-MB-231-GFP cells as previously described (PMID: 25636082). The construct was provided by donated by Dr. Julio Aguirre-Ghiso (Mt. Sinai).<br><br>BAC1.2F5 macrophages were provided by Dr. Richard Stanley, Einstein College of Medicine<br>Human Umbilical Vein Endothelial Cells (HUVECs) were obtained from Lonza<br><br>BAC1.2F5 macrophages were provided by Dr. Richard Stanley, Einstein College of Medicine
Authentication	E0771 cells were authenticated using exome gDNA sequencing. Other cell lines were authenticated by their commercial sources.
Mycoplasma contamination	All cell lines were routinely tested for mycoplasma and resulted negative (Sigma LookOut Mycoplasma PCR detection kit, cat #MO0035-1KT).
Commonly misidentified lines<br>(See ICLAC register)	None

Animals and other organisms

Policy information about studies involving animals; ARRIVE guidelines recommended for reporting animal research

Laboratory animals	Two different strains of mouse models were used: an immunocompetent C57BL/6J mouse model and an immunodeficient NUDE

	mouse model (Foxn1nu/Foxn1nu, Jackson Labs, cat #007850).<br><br>Three transgenic variants of the C57BL/6 strain of mice were used for intravital imaging: (i) a VeCad-tdTomato mouse expressing the fluorescent protein tdTomato on all endothelial cells, generated by crossing B6.FVB-Tg(Cdh5-cre)7Mlia/J (Jackson Labs, cat #006137) with B6.Cg-Gt(ROSA)26Sortm14(CAG-tdTomato)Hze/J (Jackson Labs, cat #007914) and (ii) a wild type C57BL6/J mouse (Jackson Labs, cat #000664); (iii) C57BL/6 MaFIA mice C57BL/6-Tg(CSF1R-EGFP-NGFR/FKBP1A/TNFRSF6)2Bck/J) (Jackson Labs, cat # 005070).<br><br>Mice were bred in house, except for MaFIA mice that were obtained from the Jackson Laboratory. Only female mice between 12 and 24 weeks of age were used for experiments.<br><br>Transgenic mice expressing the Polyoma Virus Middle-T (PyMT) antigen under the control of mammary tumor virus long terminal repeat (MMTV-LTR) were bred in-house. Generation of MMTV-PyMT MENA-/- mice by crossing PyMT mice with MENA heterozygotes has been described previously (PMID: 21108830).<br><br>Mice were maintained in a light, humidity and temperature controlled environment. Specifically the light-dark cycle was controlled with light from 7AM to 8PM, 45-65% humidity and 21-25C temperature.
Wild animals	This study did not involve wild animals.
Field-collected samples	This study did not involve samples collected from the field.
Ethics oversight	All procedures were conducted in accordance with the National Institutes of Health regulation concerning the care and use of experimental animals and with the approval of the with the approval of the Einstein College of Medicine Animal Care and Use Committee (IACUC).

Note that full information on the approval of the study protocol must also be provided in the manuscript.
