# Supplementary material for: Primary tumor associated macrophages activate programs of invasion and dormancy in disseminating tumor cells
Source: Nat Commun. 2022 Feb 2;13:626. doi: 10.1038/s41467-022-28076-3 (PMC8811052; doi:10.1038/s41467-022-28076-3)
Supplement: Supplementary file 7 — Source data file [file 41467_2022_28076_MOESM7_ESM.zip › Supp. Figure 16.pptx]

## Slide 1
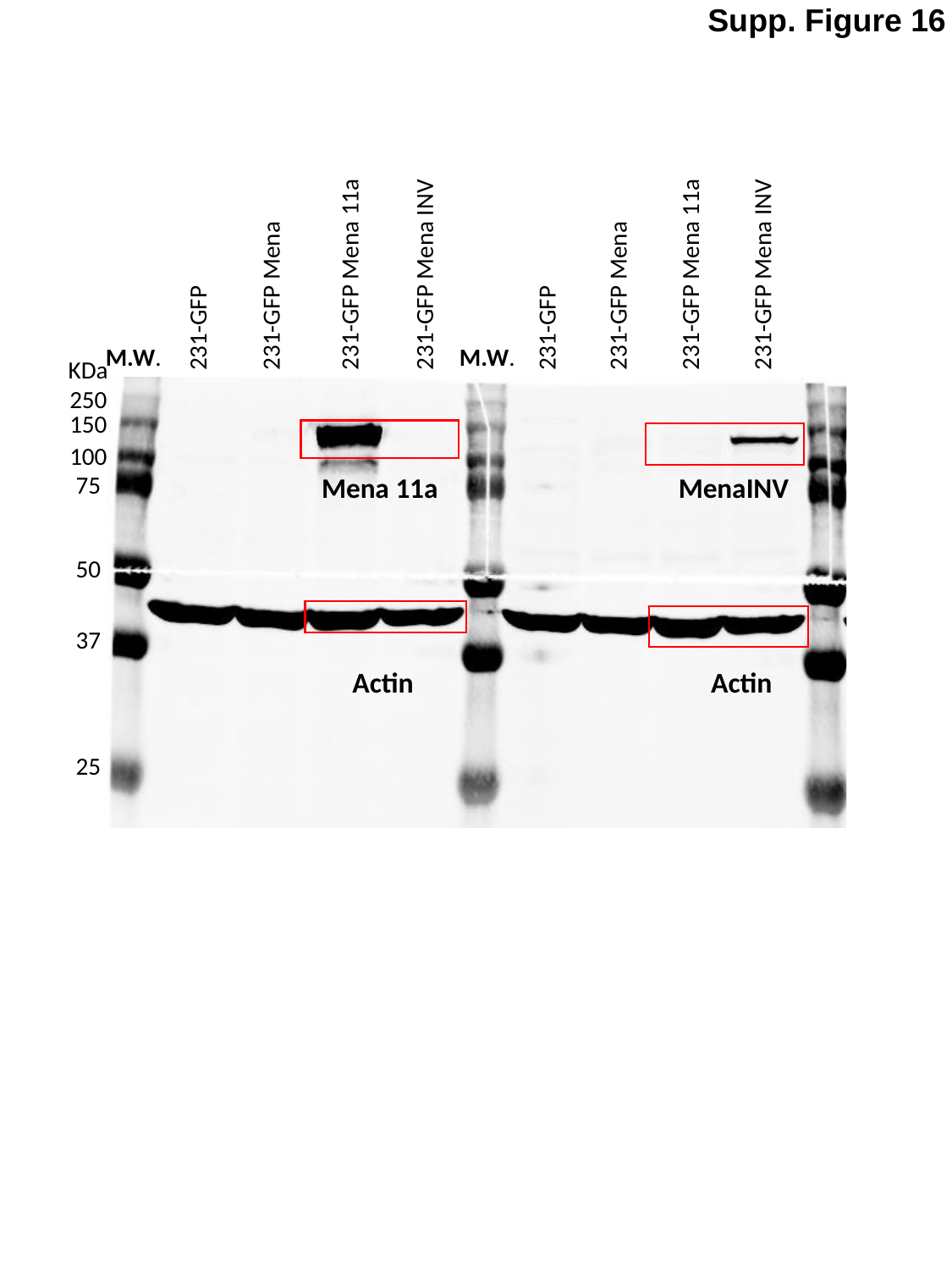

Supp. Figure 16
231-GFP Mena INV
231-GFP Mena INV
231-GFP Mena 11a
231-GFP Mena 11a
231-GFP Mena
231-GFP Mena
231-GFP
231-GFP
M.W.
 M.W.
KDa
250
150
100
75
Mena 11a
MenaINV
50
37
Actin
Actin
25
